# Supplementary material for: Molecular mechanism of Oxr1p mediated disassembly of yeast V-ATPase
Source: EMBO Rep. 2024 Apr 2;25(5):15. doi: 10.1038/s44319-024-00126-5 (PMC11094088; doi:10.1038/s44319-024-00126-5)
Supplement: Supplementary file 1 — Appendix [file 44319_2024_126_MOESM1_ESM.pdf]

Appendix

Molecular Mechanism of Oxr1p Mediated Disassembly of Yeast V-ATPase

Md. Murad Khan and Stephan Wilkens[1]

Department of Biochemistry and Molecular Biology, SUNY Upstate Medical University, Syracuse, NY 13210, USA

[1]Corresponding author: wilkenss@upstate.edu

Keywords: Vacuolar H^+-ATPase; Oxr1p; TLDc domain; Reversible disassembly

Table of Contents **Page**

Supplementary Figures

Appendix Figure S1: Disassembly of V_1 from inactive vacuoles. 2

Appendix Figure S2: Effect of different forms of Oxr1p on the activity of purified vacuoles. 4

Appendix Figure S3: Purified wild type V-ATPase. 5

Appendix Figure S4: Comparison of cryoEM structures of V_1(C)Oxr1p and autoinhibited V_1, and cryoEM maps of wild type V_1V_oND in rotary states 1 & 2. 6

Supplementary Tables

Appendix Table S1: Yeast strains used in this study. 8

Appendix Table S2. List of primers used in this study. 10

Supplementary References 12

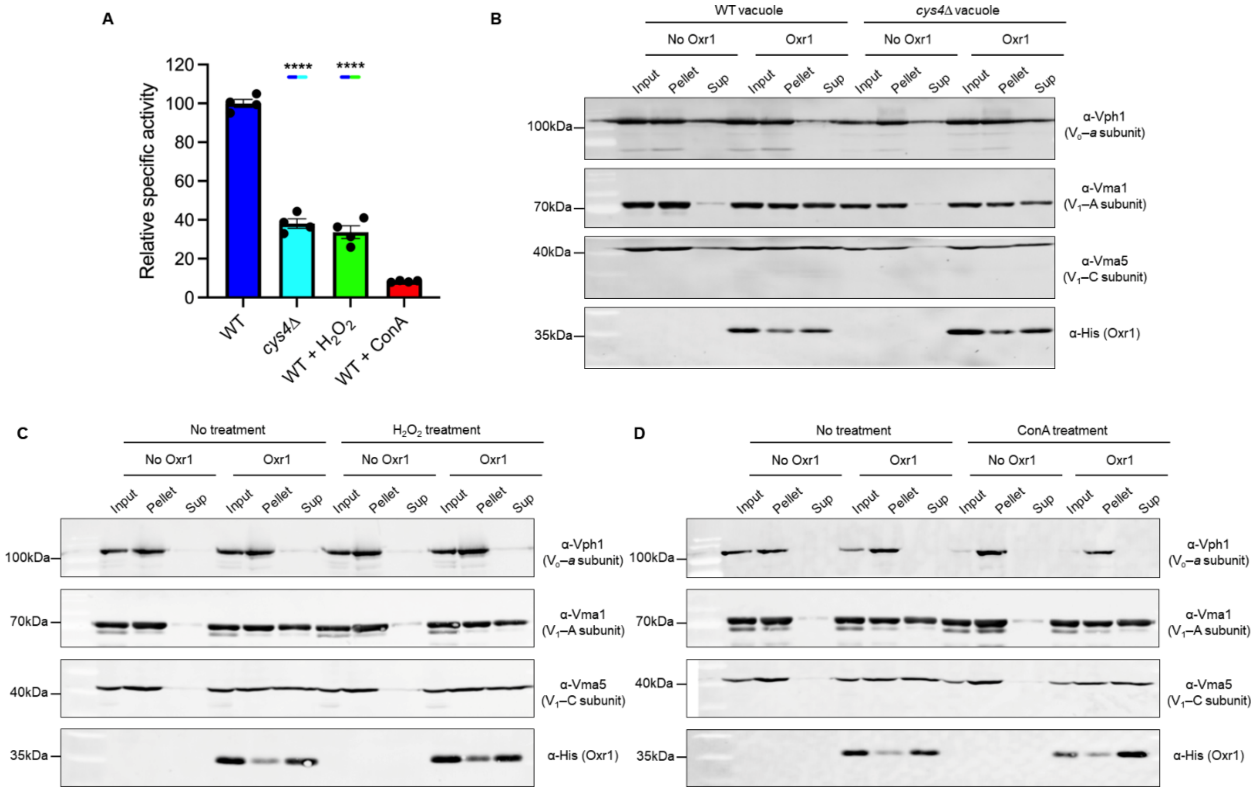

Appendix Fig S1. Disassembly of V_1 from inactive vacuoles.

A ATPase activities of vacuoles from wild type (blue), *cys4*Δ mutant (cyan) and wild type vacuoles treated with 10 mM H_2O_2 (green) or 1 µM ConA (red) for 10 min at room temperature. Relative activities were normalized against the starting activities of vacuoles from wild type (blue). Individual data points of four tests from two biological preparations are shown. Data are presented as mean ± SEM.

B Disassembly of V_1 from wild type and *cys4*Δ mutant vacuoles after incubating with or without a 1:10 ratio (w/w) of Oxr1p over vacuolar protein as determined by western blot analysis. Oxr1p is equally efficient in disassembling V_1 from both wild type and *cys4*Δ mutant vacuoles, see the equal amounts of Vma1p (V_1 subunit A) and Vma5p (V_1 subunit C) in the *sup* fraction of Oxr1p treated samples. **α**-Vph1p (V_o subunit *a*) and **α**-His (Oxr1p) blots were included as controls. A representative of three experiments from two biological preparations is shown.

C, D Disassembly of V_1 from untreated and H_2O_2 or ConA treated wild type vacuoles was determined by western blot analysis. Vacuoles were incubated with 10 mM H_2O_2 (**C**) or 1 µM ConA (**D**) for 10 min before incubating with or without a 1:10 ratio (w/w) of Oxr1p over vacuolar protein. Untreated vacuoles were included as a control. Oxr1p is equally efficient in disassembling V_1 from both active and inactive vacuoles, see the equal amounts of Vma1p (V_1 subunit A) and Vma5p (V_1 subunit C) in the *sup* fraction in the Oxr1p treatment conditions. **α-**

Vph1p (V_o subunit *a*) and **α**-His (Oxr1p) blots were included as controls. A representative of three experiments from two biological preparations is shown.

Data information: Statistical significance (A) was calculated in GraphPad Prism 9 using unpaired Student's t-test (**** indicates $P \leq 0.0001$).

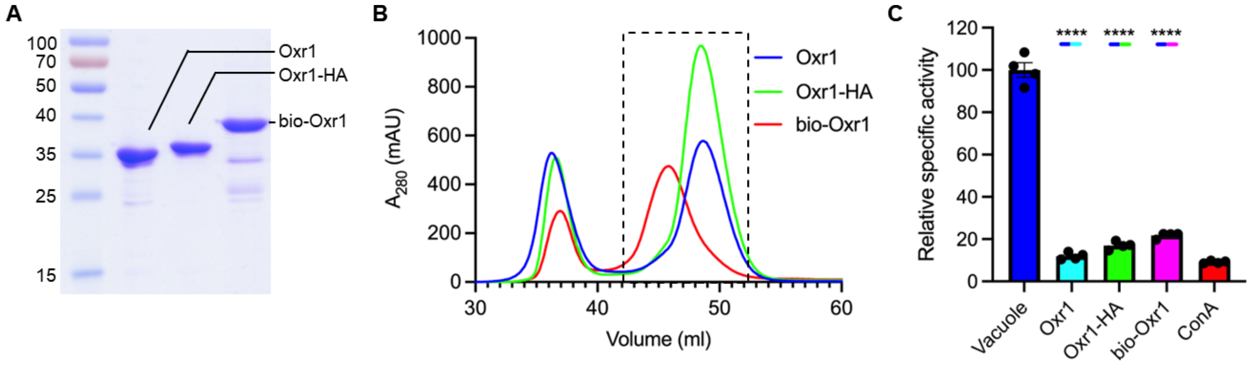

Appendix Figure S2: Effect of different forms of Oxr1p on purified vacuole activities.

A SDS-PAGE analysis of recombinant 7×His tagged Oxr1p, Oxr1p-HA, and biotinylated Oxr1p (bio-Oxr1p).

B Size exclusion chromatography (SEC) elution profile of Oxr1p (blue), Oxr1p-HA (green) and bio-Oxr1p (red) from a Superdex S75 SEC column (16 mm × 500 mm).

C V-ATPase activities of purified wild type vacuoles (blue) and of vacuoles pre-incubated with a 1:10 ratio (w/w) of Oxr1p (cyan), Oxr1p-HA (green) and bio-Oxr1p (magenta) over vacuolar protein. Relative activities were normalized against the starting activity of purified vacuoles (blue). ConA was used as an additional control. Individual data points of four tests from two biological preparations are shown.

Data information: Statistical significance (C) was calculated in GraphPad Prism 9 using unpaired Student’s t-test (**** indicates P ≤ 0.0001).

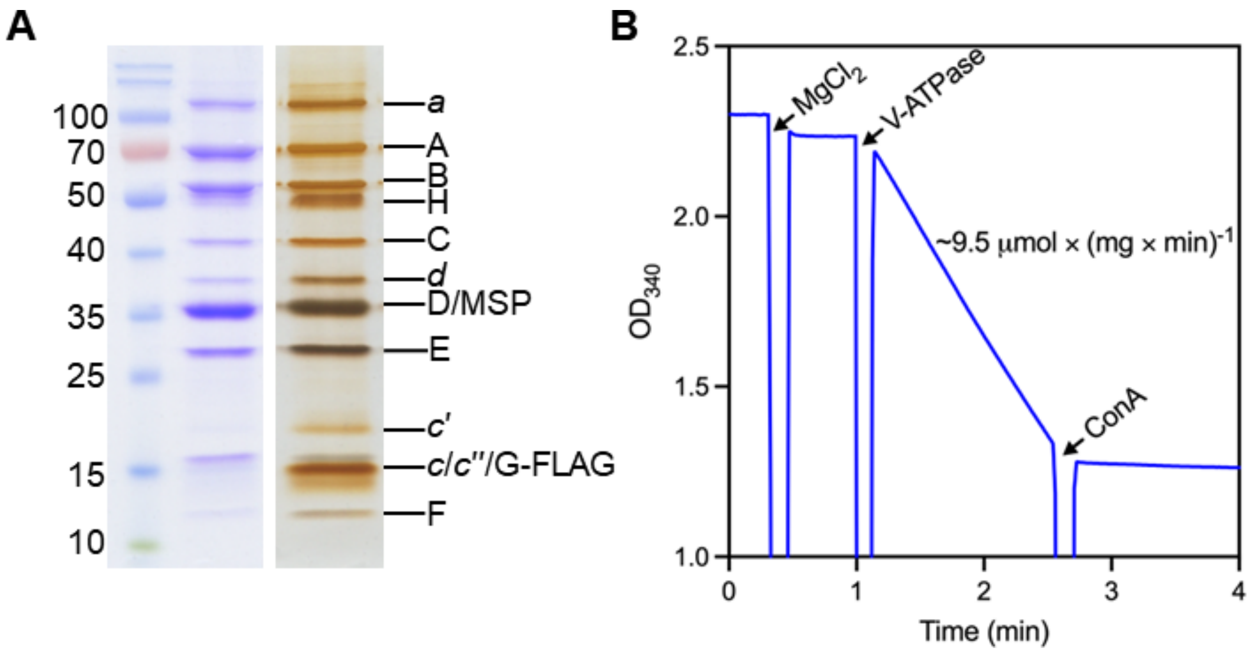

Appendix Figure S3: Purified wild type V-ATPase.

A Coomassie blue (left) and silver (right) stained SDS-PAGE gels of lipid nanodisc reconstituted wild type V-ATPase. V-ATPase was affinity captured via N-terminally FLAG tagged V_1 G subunit from detergent solubilized vacuoles and reconstituted into native lipid containing nanodiscs (V_1V_oND).

B ATPase activity of wild type V_1V_oND as measured using an ATP regenerating system showing a typical specific activity of ~9.5 μmol × (mg × min)$^{-1}$.

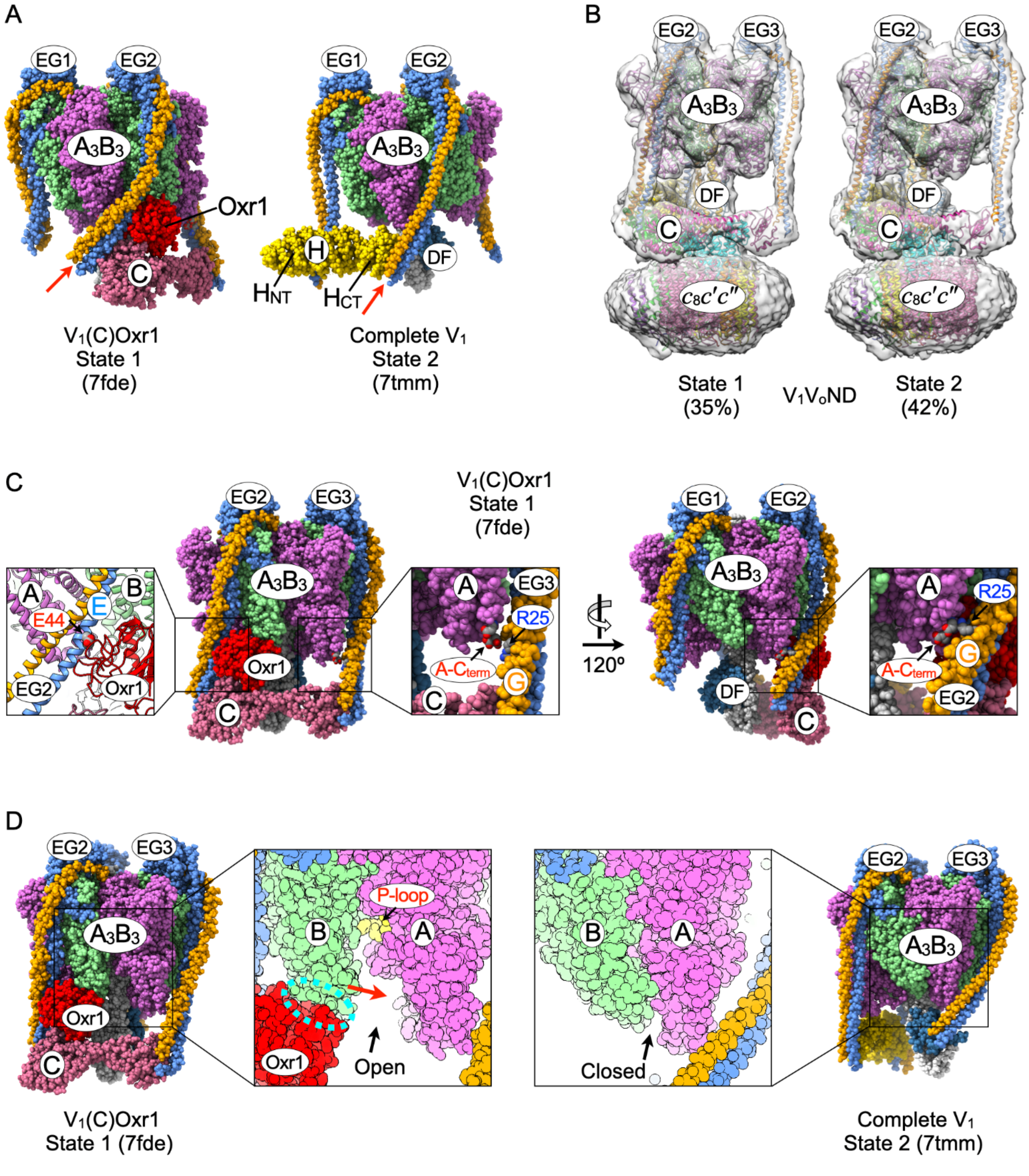
A
EG1
EG2
A_3B_3
Oxr1
C
H
H_{NT}
H_{CT}
DF
V_1(C)Oxr1
State 1
(7fde)
Complete V_1
State 2
(7tmm)
B
EG2
EG3
A_3B_3
DF
C
$c_8c'c''$
State 1
(35%)
V_1V_oND
State 2
(42%)
C
A
B
E
E44
EG2
Oxr1
EG3
R25
$A\text{-}C_{term}$
G
120º
EG1
D
P-loop
Open
Closed
V_1(C)Oxr1
State 1 (7fde)
Complete V_1
State 2 (7tmm)

Appendix Figure S4: Comparison of cryoEM structures of V_1(C)Oxr1p and autoinhibited V_1, and cryoEM maps of wild type V_1V_oND in rotary states 1 & 2.

A Comparison of cryoEM structures of V_1(C)Oxr1p (left panel, 7fde; (Khan et al, 2022)) and "Complete V_1" (right panel, 7tmm; (Vasanthakumar et al, 2022)), highlighting the bending of peripheral stator EG2 due to binding of Oxr1p (7fde) or H_{CT} (7tmm) (see red arrows). Subunit C is omitted from 7tmm for clarity.

B CryoEM analysis of V-ATPase derived from a sample without added Oxr1p resolved two major classes corresponding to rotary states 1 (35%, left panel) and 2 (42%, right panel). A dataset of 44,504 particles was analyzed.

C CryoEM structure of V_1(C)Oxr1p (7fde; (Khan et al, 2022)) in two orientations. The left panel highlights the binding site of Oxr1p near EG2, subunit C, and the C-terminal domain of the B subunit of the open catalytic site. Insets, mutations at position 44 in subunit E (Glu44; left) and 25 in G (R25; middle and right) have been shown to delay glucose withdrawal induced V-ATPase disassembly (Charsky et al, 2000; Okamoto-Terry et al, 2013).

D Closure of the open catalytic site that forms the Oxr1p binding site in rotary state 1. Left panels, cryoEM structure of V_1(C)Oxr1p (7fde), highlighting the open catalytic site, its P-loop (phosphate binding loop, yellow), and the interaction of the B subunit C-terminal domain with Oxr1p (cyan dashed oval). Right panels, the catalytic site is closed in rotary state 2 as seen for autoinhibited V_1 (7tmm).

Appendix Table S1: Yeast strains used in this study.

Strain	Genotype	Source
SF838-5Aα (wild type)	*MATα leu3-2,112 ura3-52 ade6 gal2*	Stevens et al, 1986
SF838-5Aα-*oxr1Δ* (*oxr1Δ*)	*MATα leu3-2,112 ura3-52 ade6 gal2 oxr1Δ::hyg^R*	This study
SF838-5Aα-*oxr1Δ*; pRS316-*OXR1*-HA (*oxr1*^{NP})	*MATα leu3-2,112 ura3-52 ade6 gal2 oxr1Δ::hyg^R* [pRS316]	This study
SF838-5Aα-*oxr1Δ*; YEp352-*OXR1*-HA (*oxr1*^{OE})	*MATα leu3-2,112 ura3-52 ade6 gal2 oxr1Δ::hyg^R* [YEp352]	This study
SF838-5Aα-*vma10Δ*; pRS315-FLAG-*VMA10* (V_1-FLAG)	*MATα leu3-2,112 ura3-52 ade6 gal2 vma10Δ::kan^R* [pRS315]	Sharma & Wilkens, 2017
BY4742-*vma10Δ*-*vma13Δ*; pRS315-FLAG-*VMA10* (V_1ΔH-FLAG)	*MATα his3Δ1 leu2Δ0 lys2Δ0 ura3Δ0 vma10Δ::nat^R vma13Δ::kan^R* [pRS315]	Diab et al, 2009
BY4742-*vma10Δ*-*vma13Δ*-*oxr1Δ*; pRS315-FLAG-*VMA10* (V_1ΔH$^{(ΔOxr1)}$-FLAG)	*MATα his3Δ1 leu2Δ0 lys2Δ0 ura3Δ0vma10Δ::nat^R vma13Δ::kan^R oxr1Δ::hyg^R* [pRS315]	This study
BY4742-*vma10Δ*-*vma13Δ*- *oxr1Δ*; pRS315-FLAG-*VMA10* & pRS316-*OXR1*-HA (*oxr1*^{NP}*vma*13Δ)	*MATα his3Δ1 leu2Δ0 lys2Δ0 ura3Δ0 vma10Δ::nat^R vma13Δ::kan^R oxr1Δ::hyg^R* [pRS315] [pRS316]	This study
SF838-5Aα-*OXR1*-mNeonGreen (Oxr1p-mNG)	*MATα leu3-2,112 ura3-52 ade6 gal2 OXR1::mNeonGreen-kan^R*	This study
SF838-5Aα-*oxr1Δ*; pRS316-mNeonGreen-*OXR1* (mNG-Oxr1p)	*MATα leu3-2,112 ura3-52 ade6 gal2 oxr1Δ::hyg^R* [pRS316]	This study
SF838-5Aα-*VMA5*-mNeonGreen (C-mNG)	*MATα leu3-2,112 ura3-52 ade6 gal2 VMA5::mNeonGreen-kan^R*	This study
SF838-5Aα-*oxr1Δ*-*VMA5*-mNG (*oxr1Δ*.C-mNG)	*MATα leu3-2,112 ura3-52 ade6 gal2 oxr1Δ::hyg^R VMA5::mNeonGreen-kan^R*	This study

SF838-5Aα-*oxr1*Δ-*VMA5*-mNG; pRS316-*OXR1*-HA ($Oxr1^{NP}$.C-mNG)	*MATα leu3-2,112 ura3-52 ade6 gal2 oxr1Δ::hyg*^{R} *VMA5::mNeonGreen-kan*^{R} [pRS316]	This study
SF838-5Aα-*vma1*Δ (*vma1*Δ)	*MATα leu2-3,112 ura3-52 ade6 vma1Δ::LEU2*	Liu & Kane, 1996
SF838-5Aα-*cys4*Δ (*cys4*Δ)	*MATα leu2-3,112 ura3-52 ade6 his4–419 cys4Δ::LEU2*	Oluwatosin & Kane, 1997

Appendix Table S2. List of primers used in this study.

Primer name	Oligonucleotide sequence (5′ to 3′)
Oxr1del FWD	GAC AAC TGA TTT CCA GCC ATT CAT TAT TCA GTG AGG AGC GAC AAG CTT GCC TCG TCC CCG C
Oxr1del REV	CTC ATT CAT AAA TAC ATA GTA TGA TAT TTA AAT AGA TGA CAT TAA AGC CTT CGA GCG TCC
Gen-Oxr1CT-mNG F	CAA AAA ATT TTC TAT AGT AGC TTT GGA AGT ATG GCG TGT AGG AGG AGG CGC AGC CAT GGT G
Gen-Oxr1CT-mNG R	CAT TCA TAA ATA CAT AGT ATG ATA TTT AAA TAG ATG ACA CTA CAG TAT AGC GAC CAG CAT
Scarletvma5CTF	CTC TTG TCG ACA CAG AGT ATG AAC CAT TTG TGA TGT ATA TAA TCA ATT TAG GAG GCG CAG CCA TGG TGA GCA AGG
mNeonvma5CTR	CTA AAA AAA AAA CAG AAA TAT ATA TTA ATC TAA GTT AGT ATT ATA AAT CGA CAG TAT AGC GAC CAG CAT TCA CAT ACG
Oxr1UTR_pMECS_FWD	CAA TTT CAC AAG CTT AAG GAG ACA GTA CAT CCG TGT GGC AAT AAT CTC AAT G
Oxr1_HA_pMECS_REV	ACG GGT ATG CGG CCG CTG AGG ATC CTA CAC GCC ATA CTT CCA
Oxr1_3UTR F	CGA CGT TCC GGA CTA CGG TTC CTA GTG TCA TCT ATT TAA ATA TCA TA
Oxr1_3UTR R	GAG GTT TTG CTA AAC AAC TTT CAA CAG TGT GGC AAC TGC CTT TCC C
Oxr1UTR_pRS316 F	GCG GTG GCG GCC GCT CTA GAC CGT GTG GCA ATA ATC TCA ATG
Oxr1UTR_pRS316 R	CCC CTC GAG GTC GAC GGT ATC GGT GGC AAC TGC CTT TCC C
Oxr1_HA_YEp352_F	GCT ATG ACC ATG ATT ACG AAT TCG AGC TCG ATG TTT GGA GTC AAG GAT GCT

Oxr1_HA_YEp352_R	AGC TTG CAT GCC TGC AGG TCG ACT AGG AAC CGT AGT CCG GAA CGT C
mCher_OxUTR_F	GCC ATT CAT TAT TCA GTG AGG AGC GAC AAT GGT GAG CAA GGG CGA G
mCher_OxUTR_R	ACG GGT ATG CGG CCG CTG AGG ACT TGT ACA GCT CGT CCA TGC
Oxr1_mCherry_F	GTA CAA GTC CTC AGC GGC CGC AAT GTT TGG AGT CAA GGA TGC T
Oxr1_mCherry_R	TAC ATA GTA TGA TAT TTA AAT AGA TGA CAC TAT CCT ACA CGC CAT ACT TCC A
mNG_Oxr1_R	CAA ACA TTG CGG CCG CTG AGG ACT TGT ACA GCT CGT CCA TGC
Oxr1_ETF	GAA GGA GAT ATA CCA TGG GTC ATC ATC ATC ATC ATC ATC ACT TTG GAG TCA AGG ATG CTA TAT TCA AG
Oxr1_HA_ETR	GTG CTC GAG TGC GGC CGC AAG CCT AGG AAC CGT AGT CCG GA
Oxr1_pET28a_F	AGT TCT GTT CCA GGG GCC CAA GAT GTT TGG AGT CAA GGA TGC T
Oxr1_pET28a_R	GTG CTC GAG TGC GGC CGC AAG CCT ATC CTA CAC GCC ATA CTT CC

References

Charsky CM, Schumann NJ, Kane PM (2000) Mutational analysis of subunit G (Vma10p) of the yeast vacuolar H+-ATPase. *J Biol Chem* 275: 37232-37239

Diab H, Ohira M, Liu M, Cobb E, Kane PM (2009) Subunit interactions and requirements for inhibition of the yeast V1-ATPase. *J Biol Chem* 284: 13316-13325

Khan MM, Lee S, Couoh-Cardel S, Oot RA, Kim H, Wilkens S, Roh SH (2022) Oxidative stress protein Oxr1 promotes V-ATPase holoenzyme disassembly in catalytic activity-independent manner. *EMBO J* 41: e109360

Liu J, Kane PM (1996) Mutational analysis of the catalytic subunit of the yeast vacuolar proton-translocating ATPase. *Biochemistry* 35: 10938-10948

Okamoto-Terry H, Umeki K, Nakanishi-Matsui M, Futai M (2013) Glu-44 in the amino-terminal alpha-helix of yeast vacuolar ATPase E subunit (Vma4p) has a role for VoV1 assembly. *J Biol Chem* 288: 36236-36243

Oluwatosin YE, Kane PM (1997) Mutations in the CYS4 gene provide evidence for regulation of the yeast vacuolar H+-ATPase by oxidation and reduction in vivo. *J Biol Chem* 272: 28149-28157

Sharma S, Wilkens S (2017) Biolayer interferometry of lipid nanodisc-reconstituted yeast vacuolar H+ -ATPase. *Protein Sci* 26: 1070-1079

Stevens TH, Rothman JH, Payne GS, Schekman R (1986) Gene dosage-dependent secretion of yeast vacuolar carboxypeptidase Y. *J Cell Biol* 102: 1551-1557

Vasanthakumar T, Keon KA, Bueler SA, Jaskolka MC, Rubinstein JL (2022) Coordinated conformational changes in the V(1) complex during V-ATPase reversible dissociation. *Nat Struct Mol Biol* 29: 430-439
