# Supplementary figures and images for: Molecular mechanism of Oxr1p mediated disassembly of yeast V-ATPase
Source: EMBO Rep. 2024 Apr 2;25(5):15. doi: 10.1038/s44319-024-00126-5 (PMC11094088; doi:10.1038/s44319-024-00126-5)

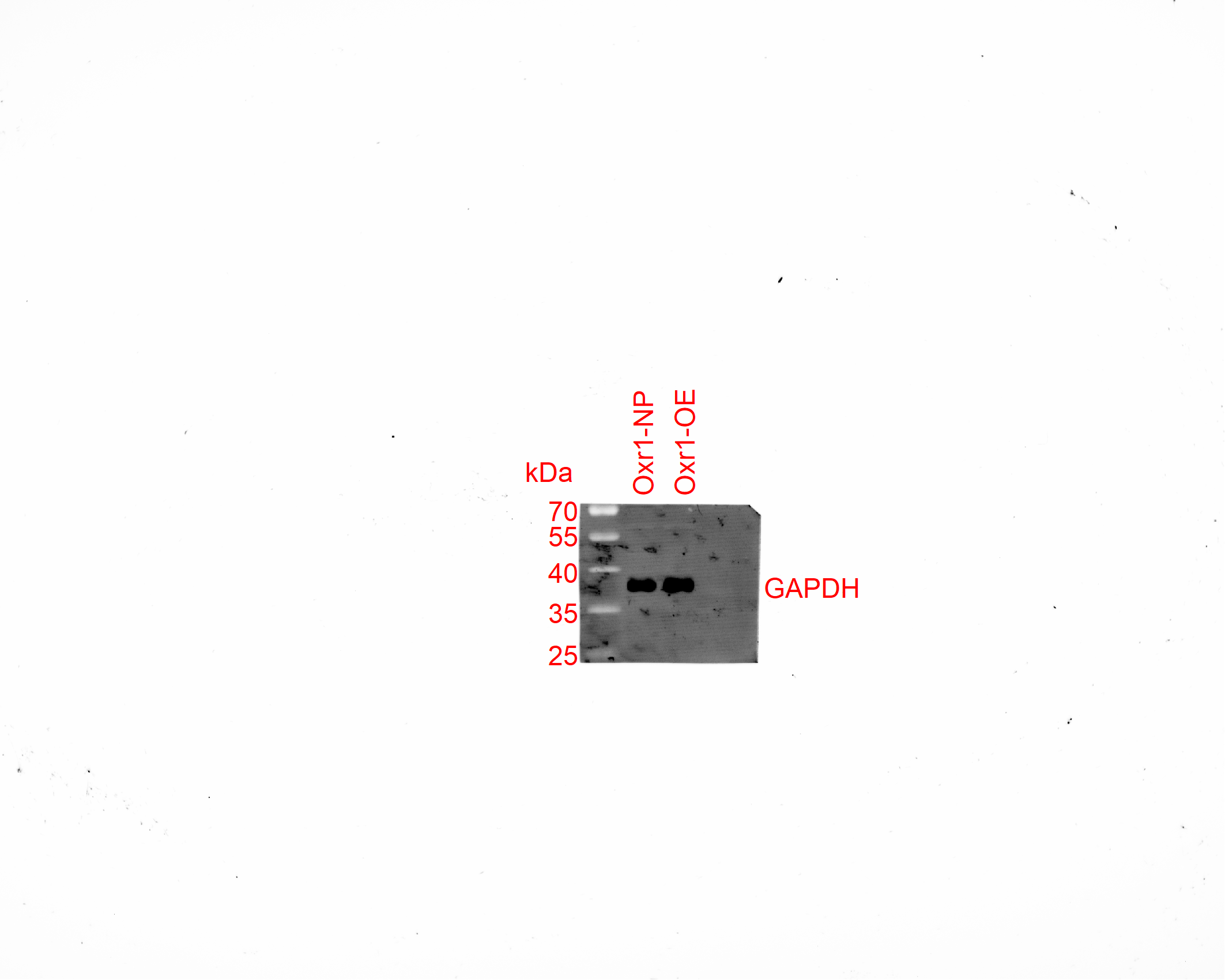

Supplement: Supplementary file 3 — Source data Fig. 2 [file 44319_2024_126_MOESM3_ESM.zip › Figure 2/2A/2A_WB_GAPDH.tif]

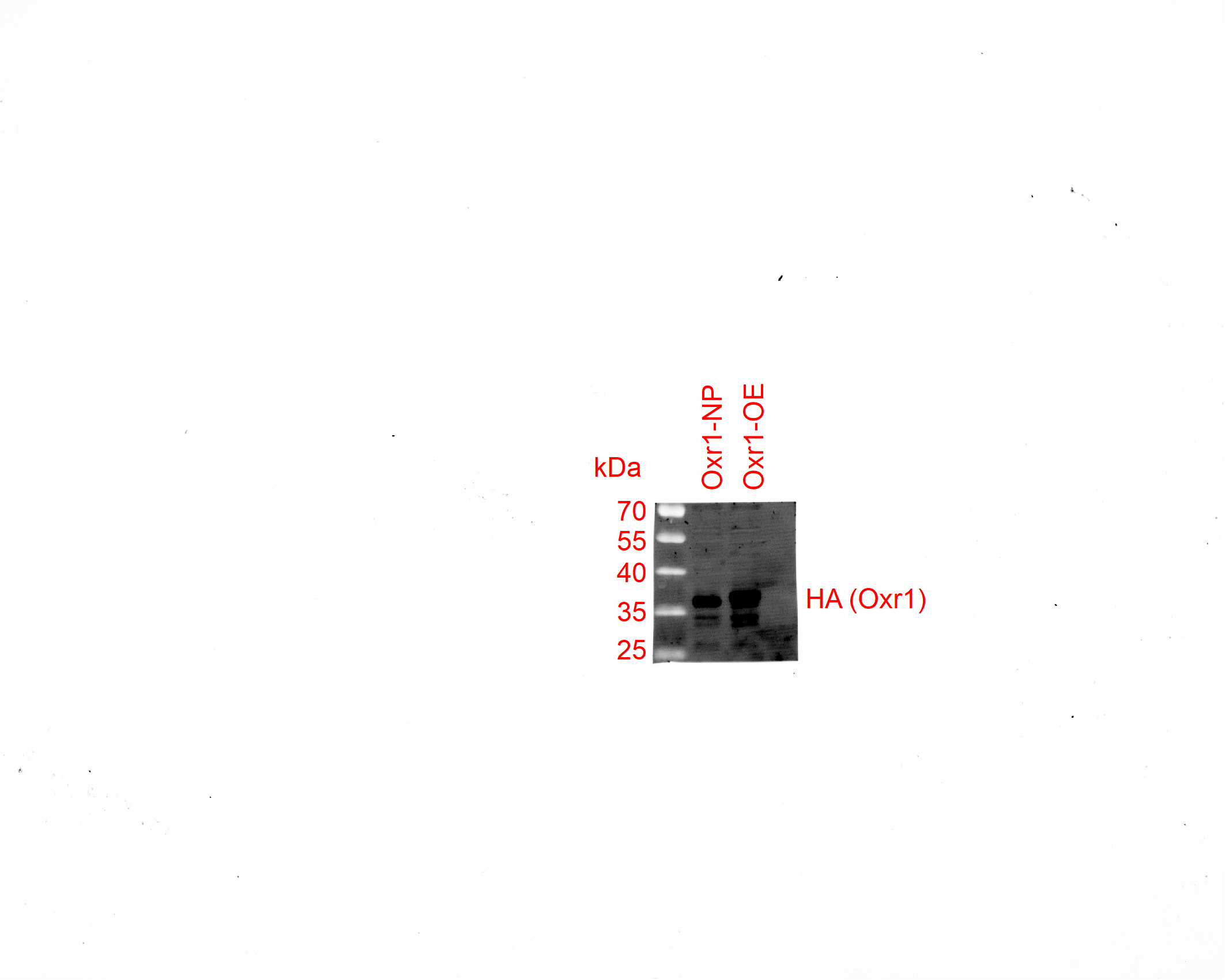

Supplement: Supplementary file 3 — Source data Fig. 2 [file 44319_2024_126_MOESM3_ESM.zip › Figure 2/2A/2A_WB_HA (Oxr1).tif]

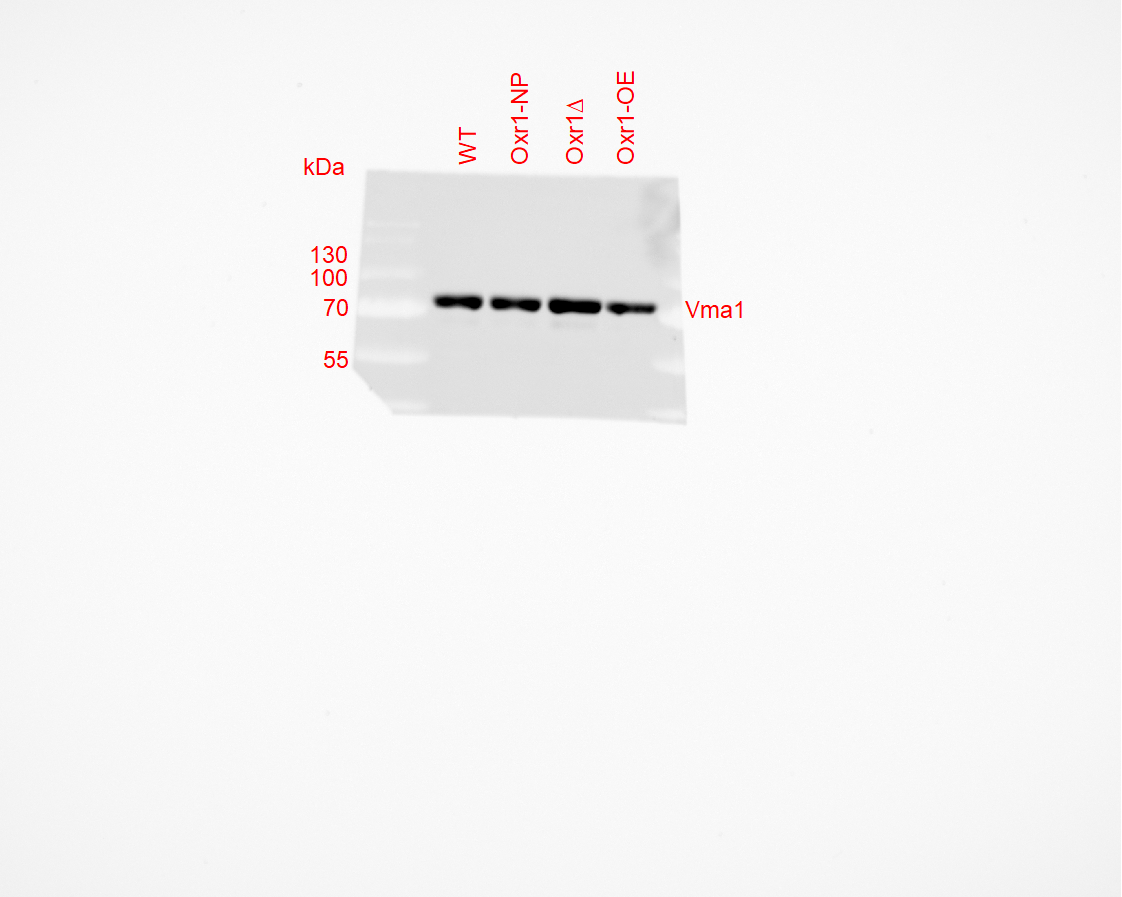

Supplement: Supplementary file 3 — Source data Fig. 2 [file 44319_2024_126_MOESM3_ESM.zip › Figure 2/2D/2D_WB_Vma1.tif]

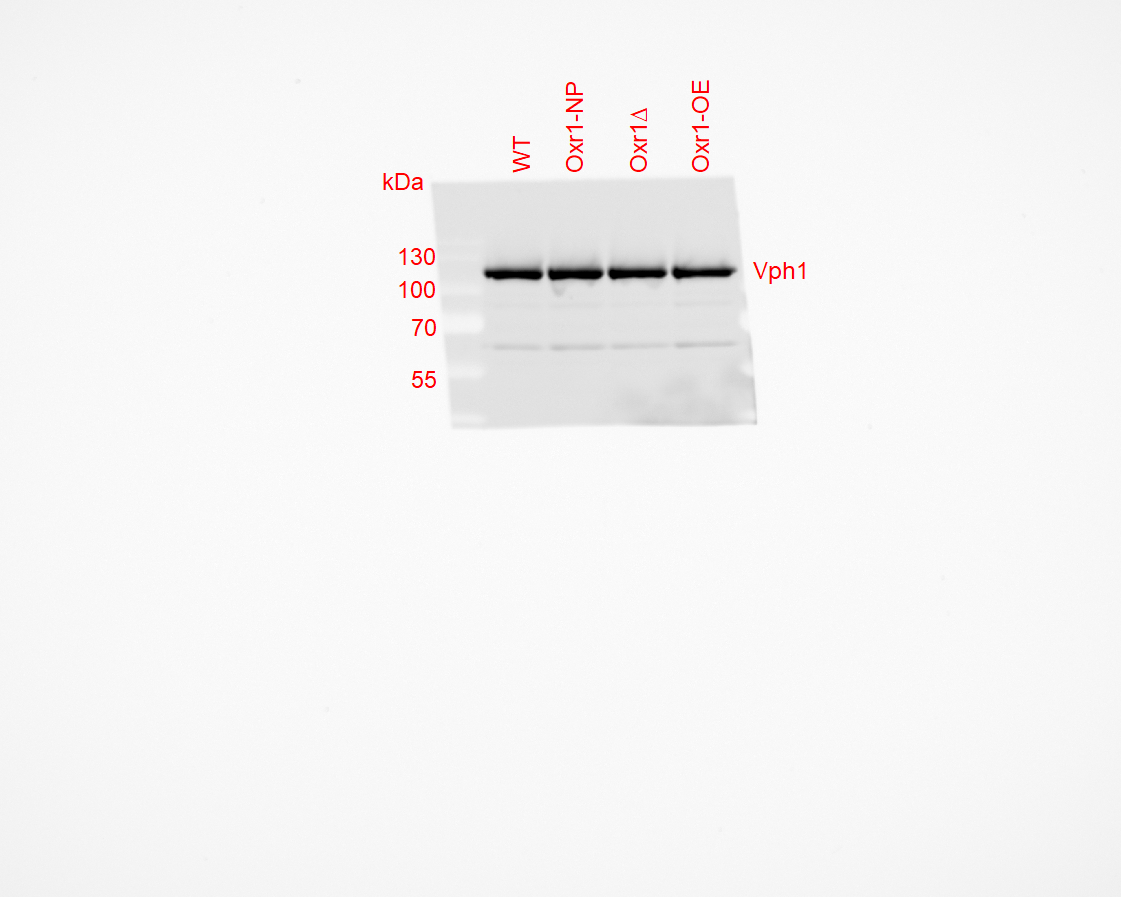

Supplement: Supplementary file 3 — Source data Fig. 2 [file 44319_2024_126_MOESM3_ESM.zip › Figure 2/2D/2D_WB_Vph1.tif]

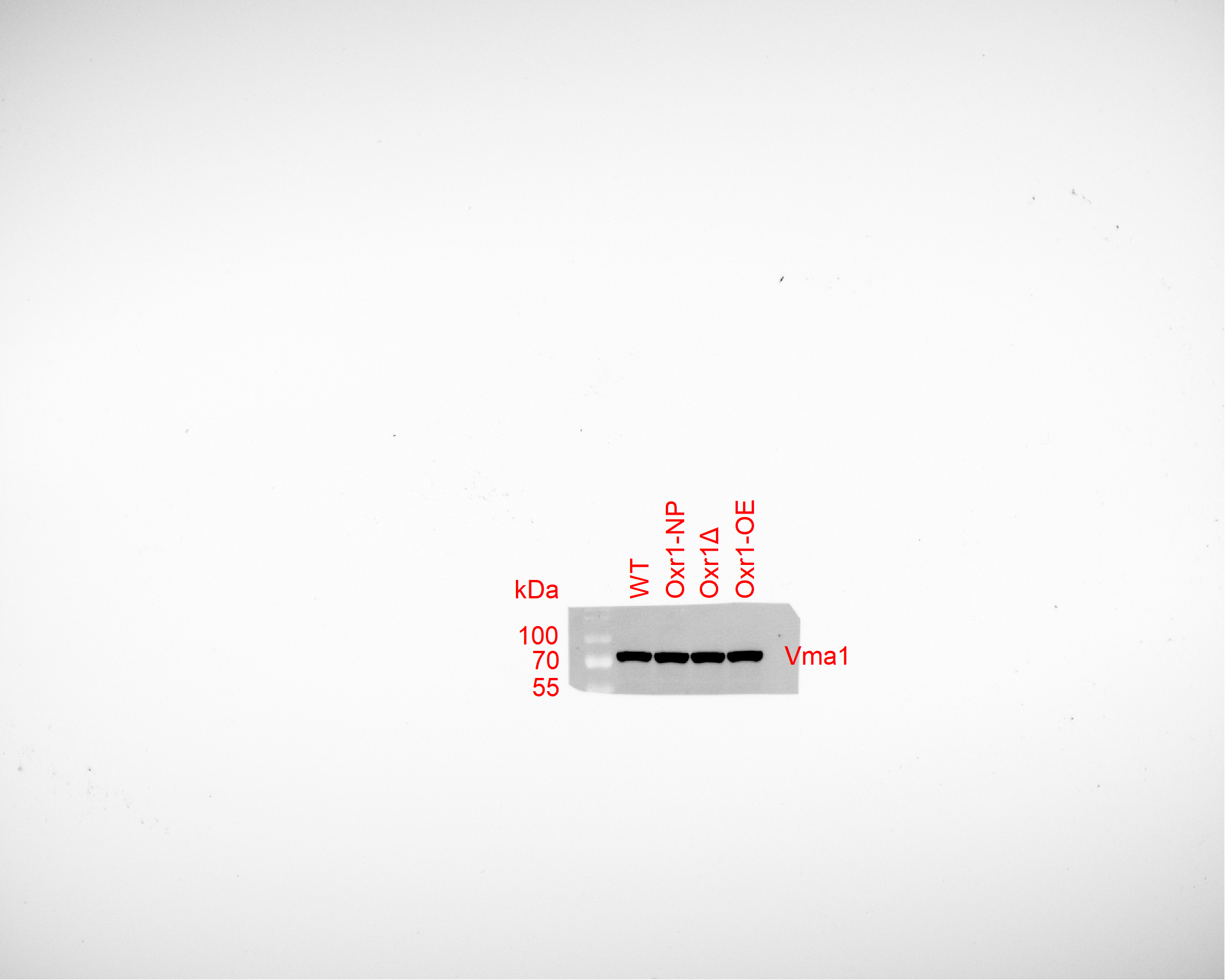

Supplement: Supplementary file 3 — Source data Fig. 2 [file 44319_2024_126_MOESM3_ESM.zip › Figure 2/2E/2E_WB_Vma1.tif]

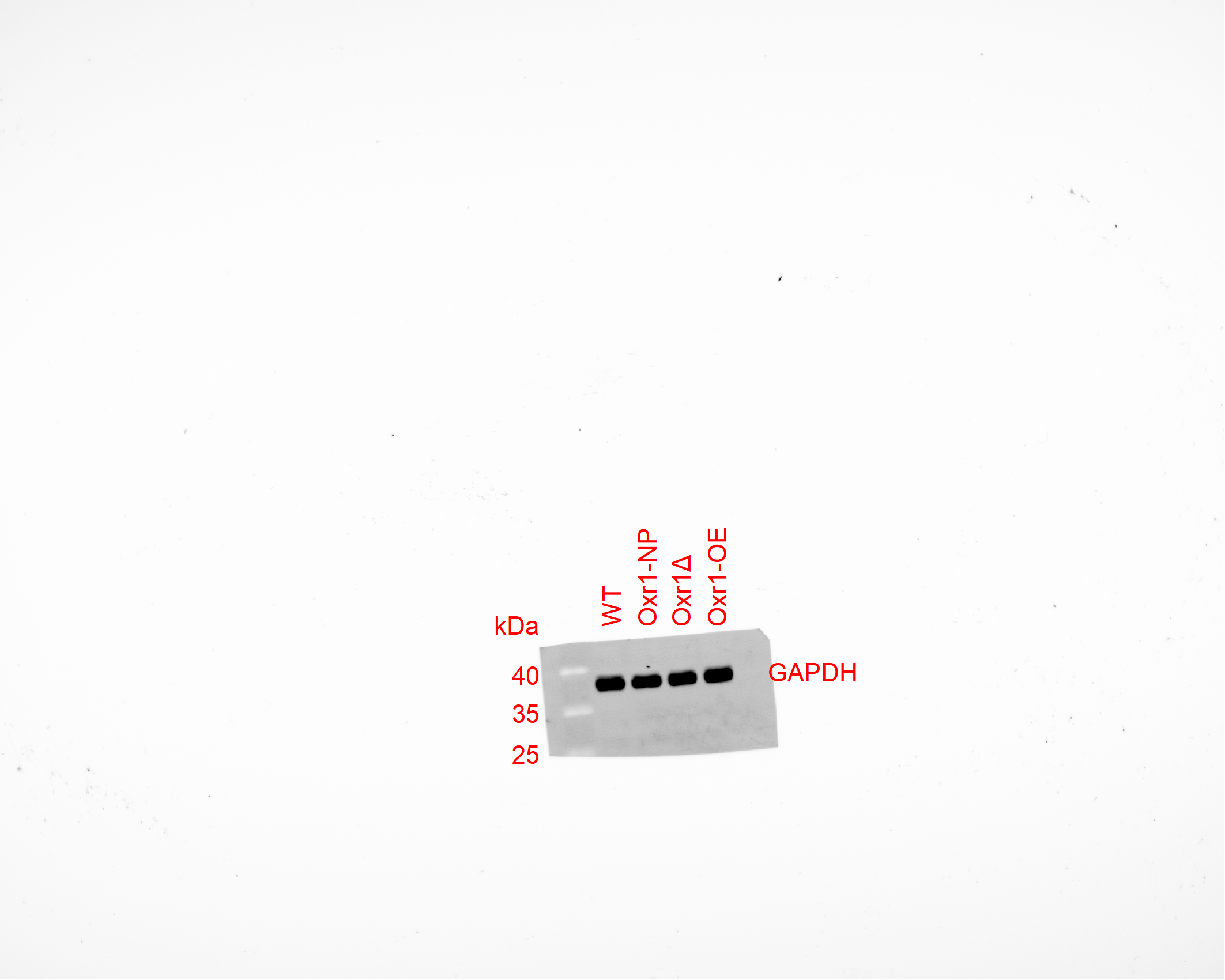

Supplement: Supplementary file 3 — Source data Fig. 2 [file 44319_2024_126_MOESM3_ESM.zip › Figure 2/2E/2E_WB_GAPDH.tif]

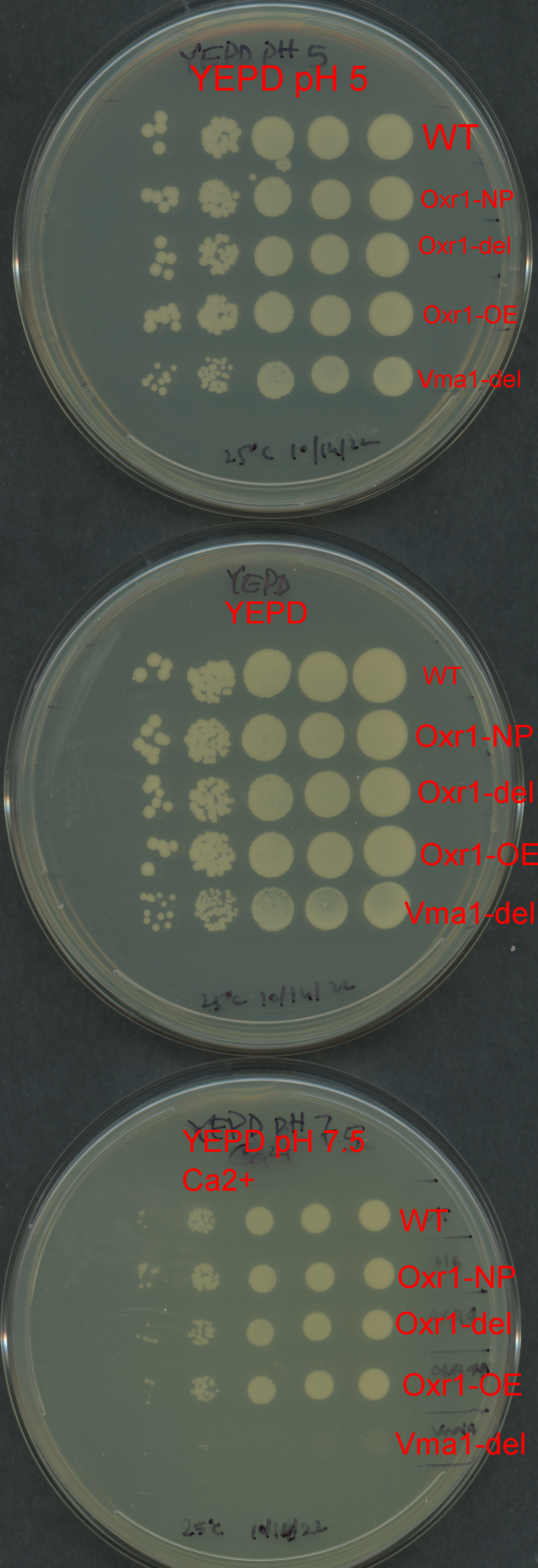

Supplement: Supplementary file 3 — Source data Fig. 2 [file 44319_2024_126_MOESM3_ESM.zip › Figure 2/2B/2B_Image_Top panel (25oC).tiff]

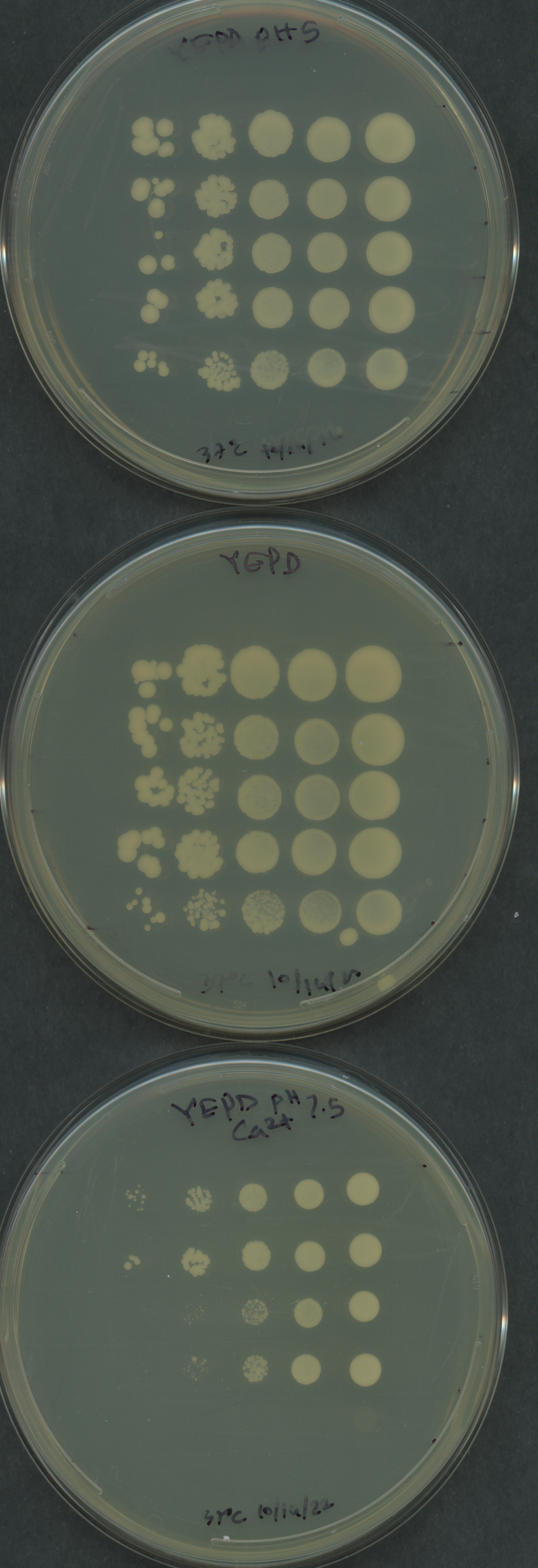

Supplement: Supplementary file 3 — Source data Fig. 2 [file 44319_2024_126_MOESM3_ESM.zip › Figure 2/2B/2B_Image_Bottom panel (37oC).tiff]

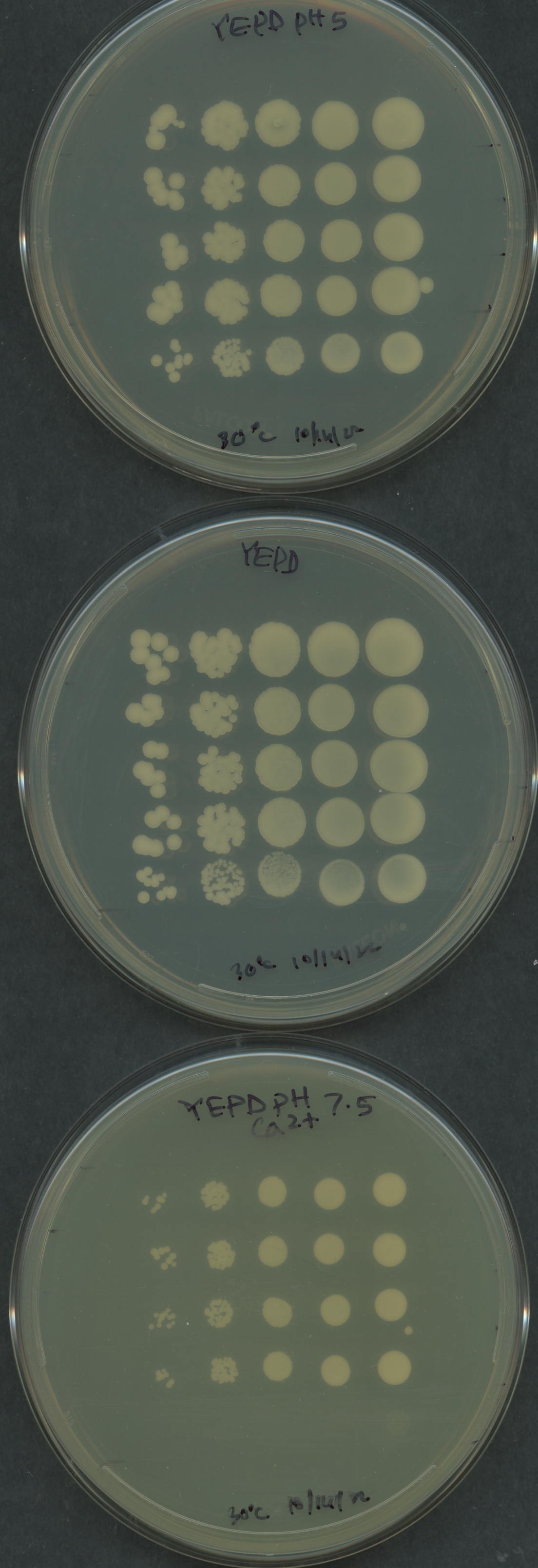

Supplement: Supplementary file 3 — Source data Fig. 2 [file 44319_2024_126_MOESM3_ESM.zip › Figure 2/2B/2B_Image_Middle panel (30oC).tiff]

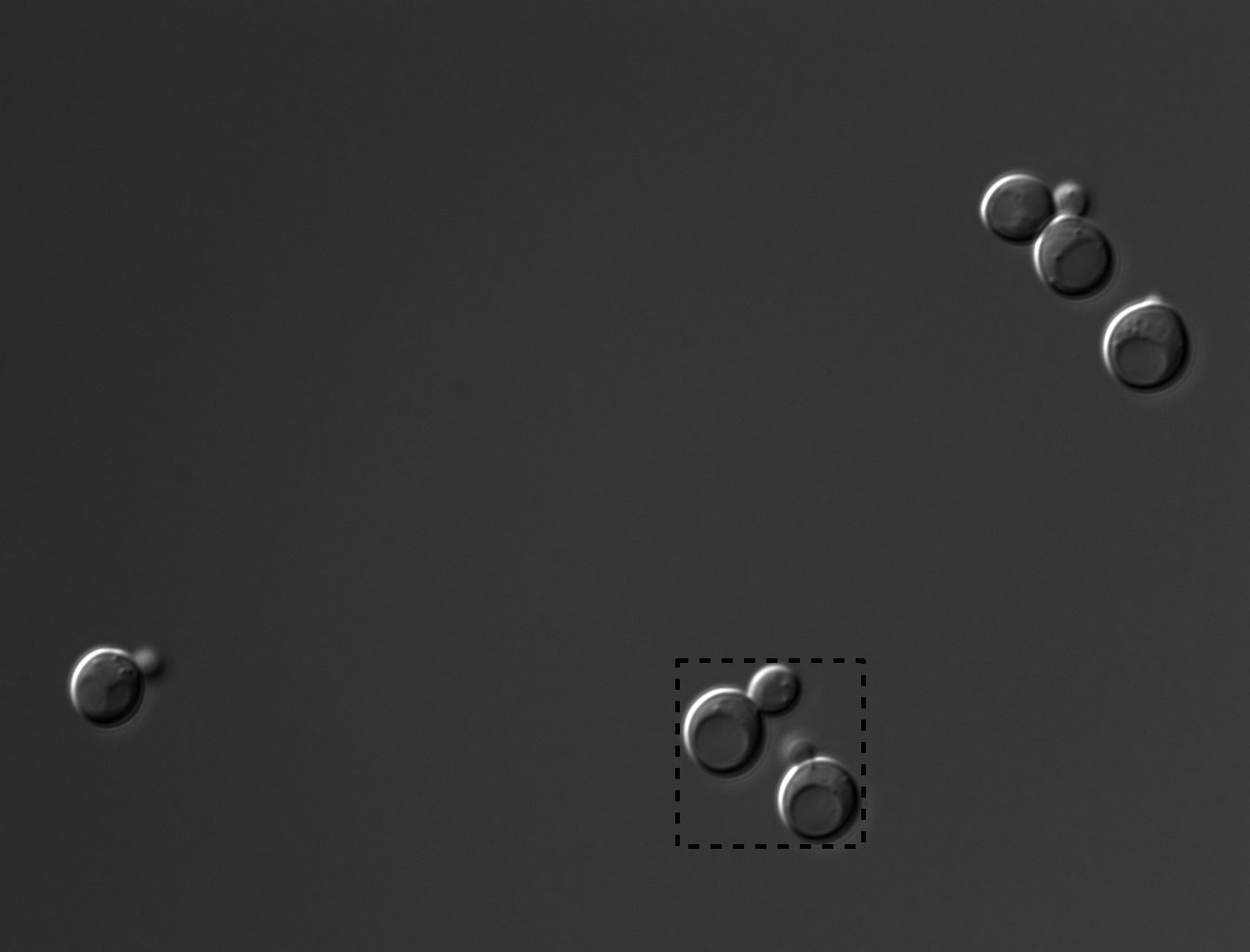

Supplement: Supplementary file 4 — Source data Fig. 3 [file 44319_2024_126_MOESM4_ESM.zip › Figure 3/3A/3A_Image_Glucose deprived_DIC.jpg]

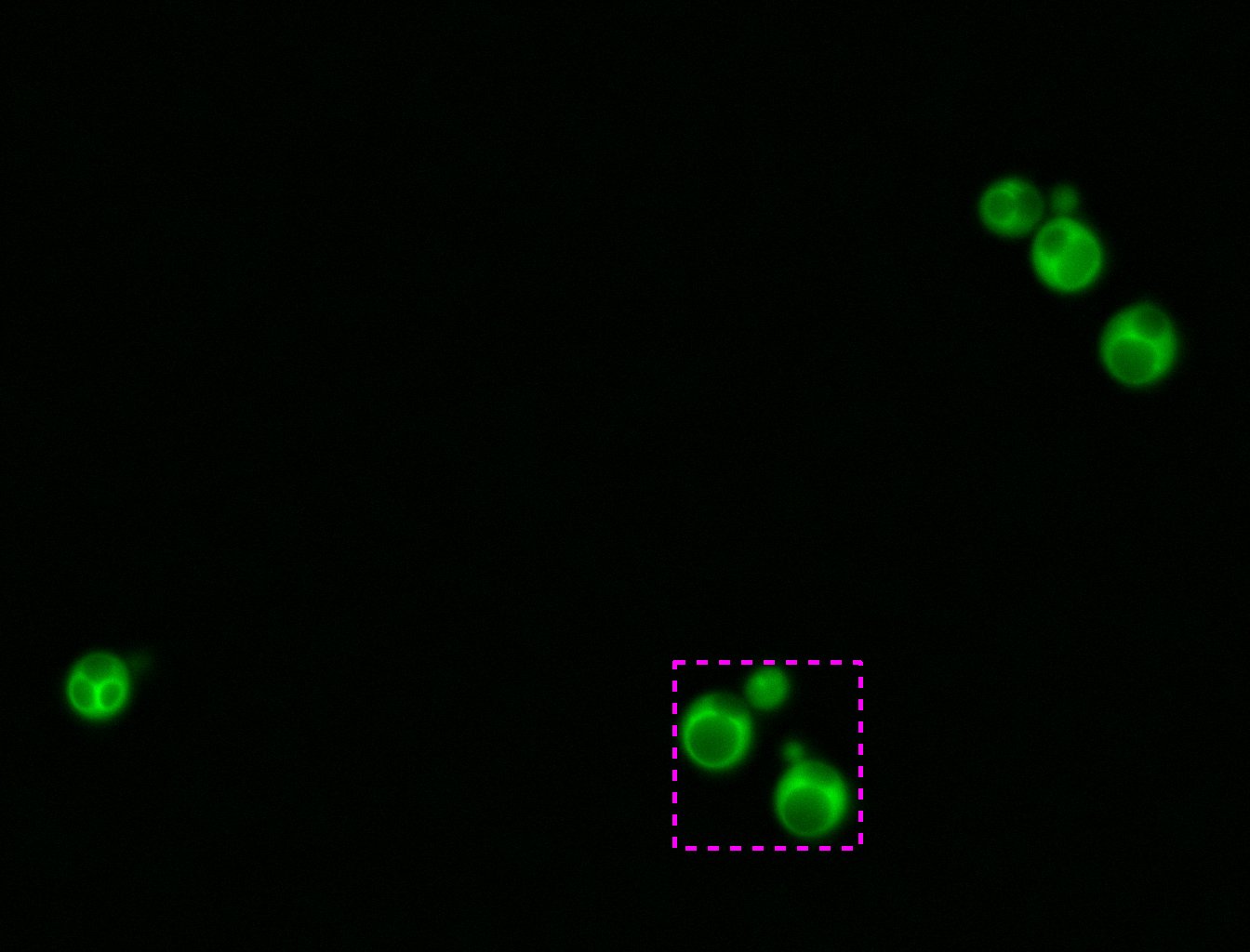

Supplement: Supplementary file 4 — Source data Fig. 3 [file 44319_2024_126_MOESM4_ESM.zip › Figure 3/3A/3A_Image_Glucose deprived_Green.jpg]

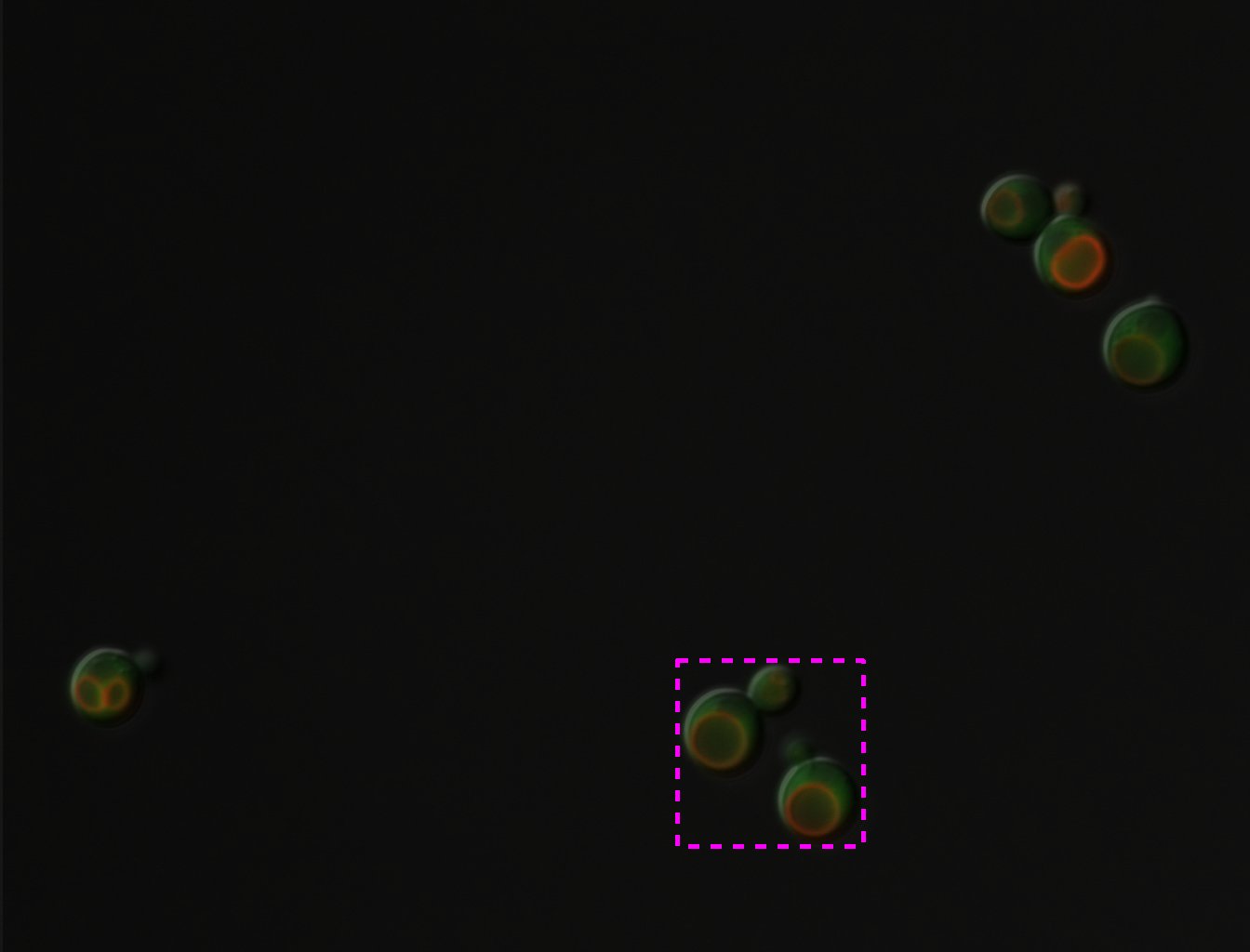

Supplement: Supplementary file 4 — Source data Fig. 3 [file 44319_2024_126_MOESM4_ESM.zip › Figure 3/3A/3A_Image_Glucose deprived_overlay.jpg]

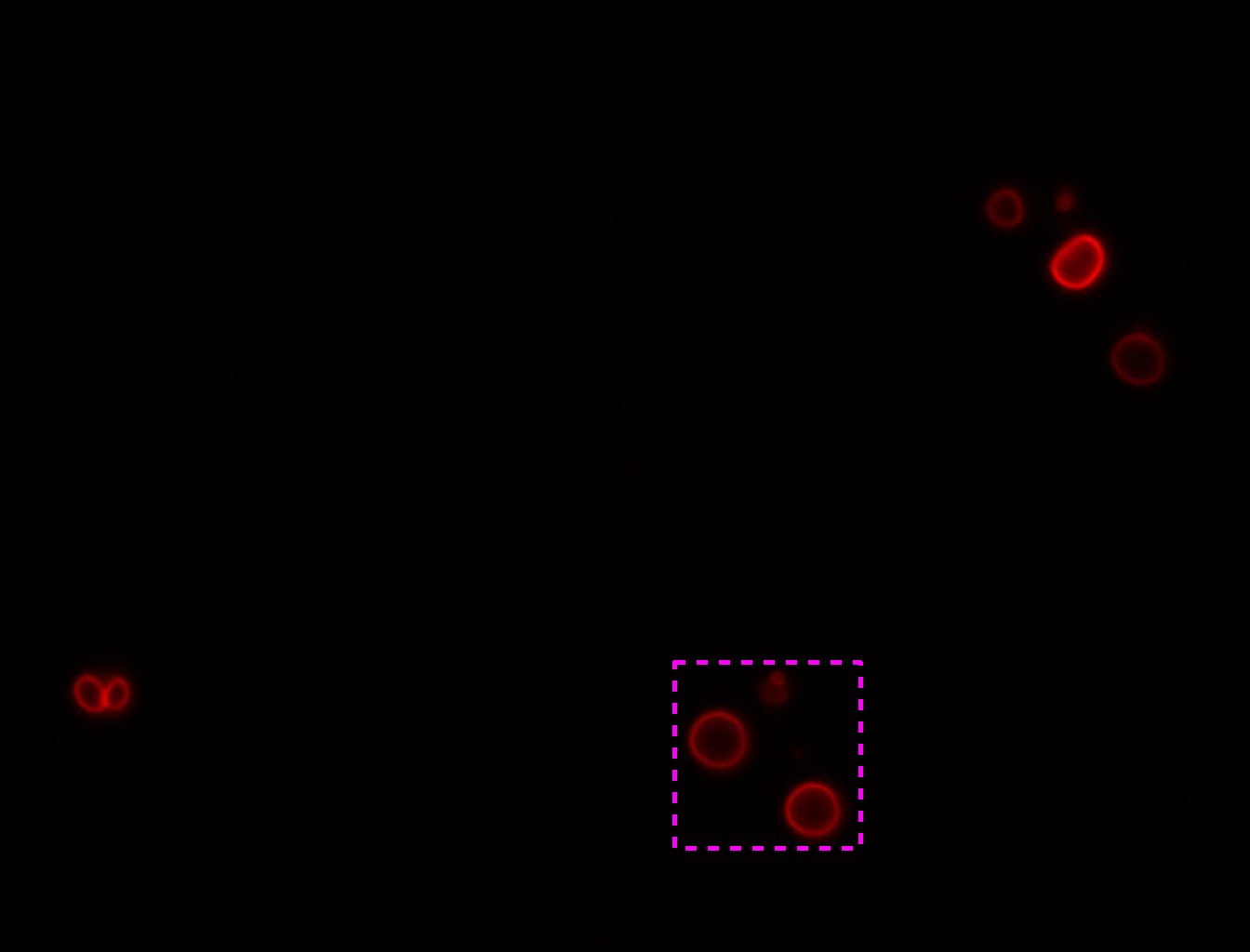

Supplement: Supplementary file 4 — Source data Fig. 3 [file 44319_2024_126_MOESM4_ESM.zip › Figure 3/3A/3A_Image_Glucose deprived_Red.jpg]

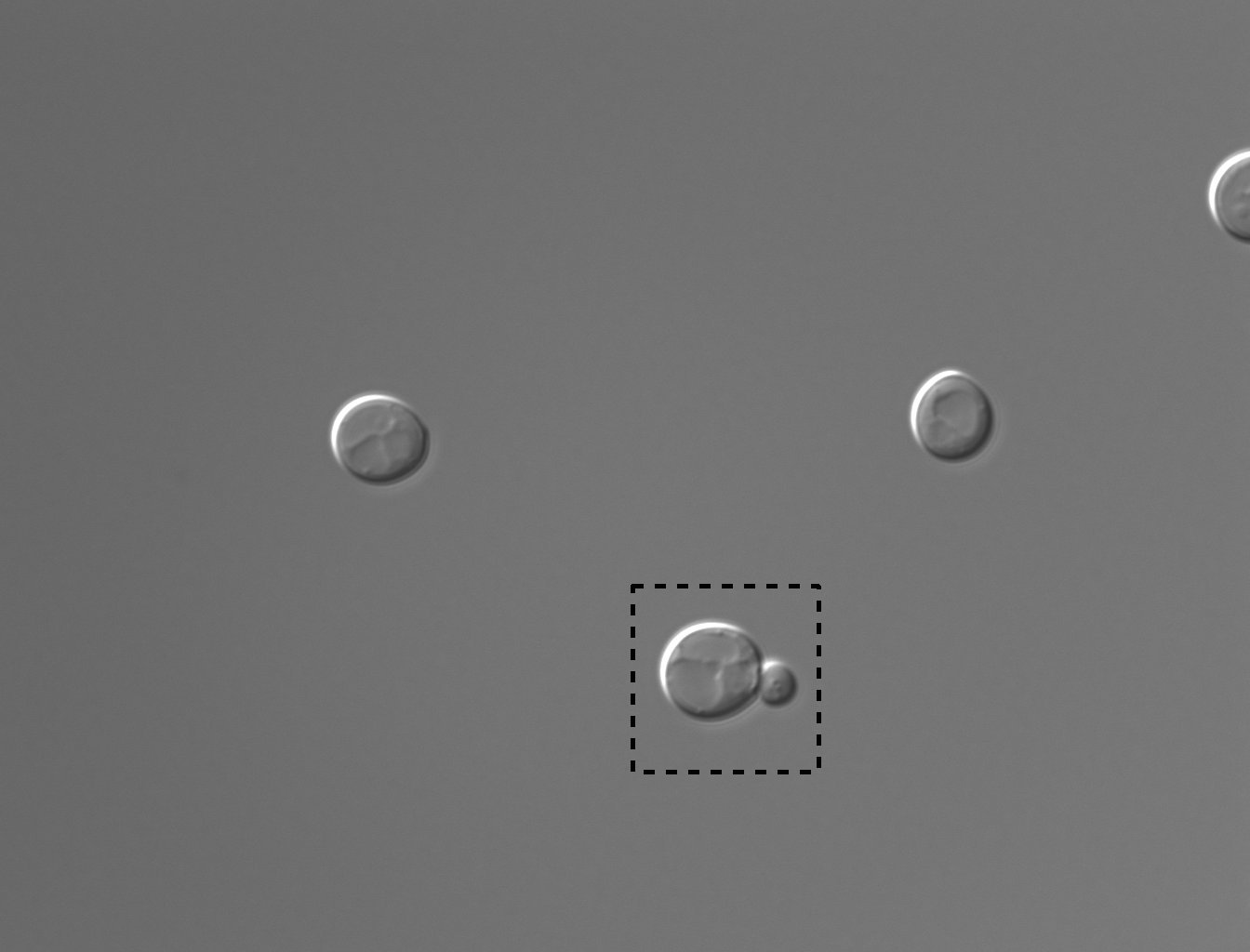

Supplement: Supplementary file 4 — Source data Fig. 3 [file 44319_2024_126_MOESM4_ESM.zip › Figure 3/3A/3A_Image_Glucose readd_DIC.jpg]

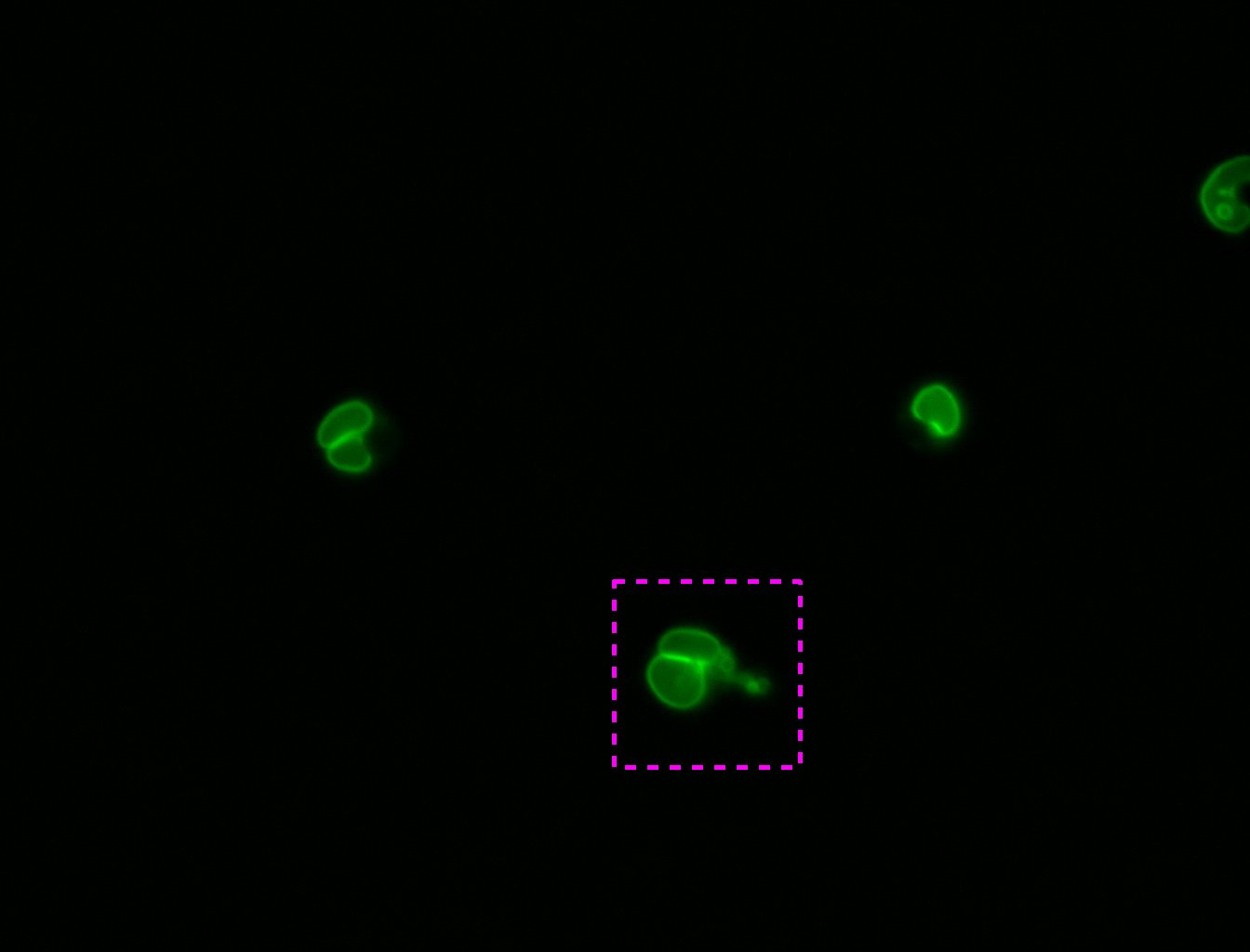

Supplement: Supplementary file 4 — Source data Fig. 3 [file 44319_2024_126_MOESM4_ESM.zip › Figure 3/3A/3A_Image_Glucose readd_Green.jpg]

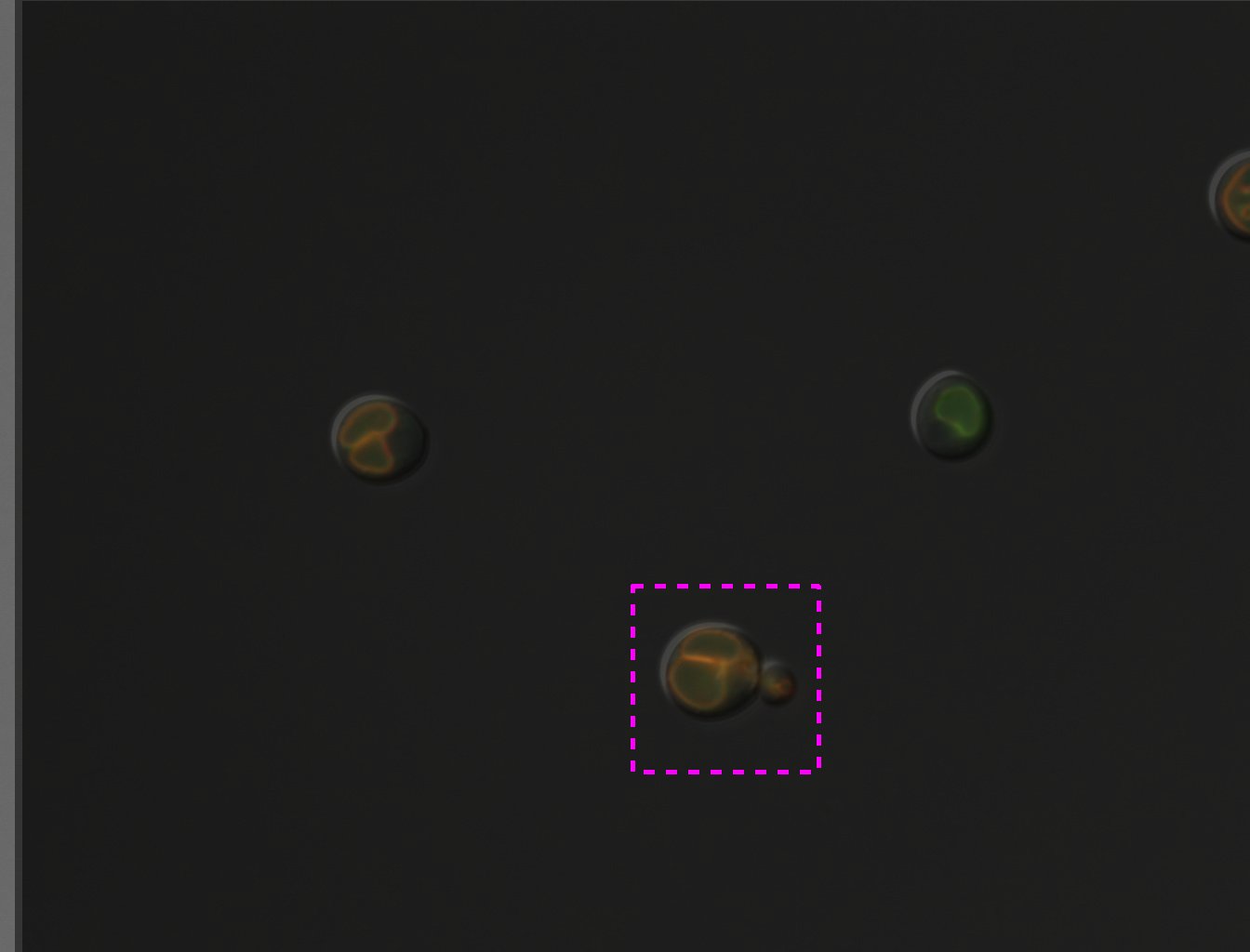

Supplement: Supplementary file 4 — Source data Fig. 3 [file 44319_2024_126_MOESM4_ESM.zip › Figure 3/3A/3A_Image_Glucose readd_overlay.jpg]

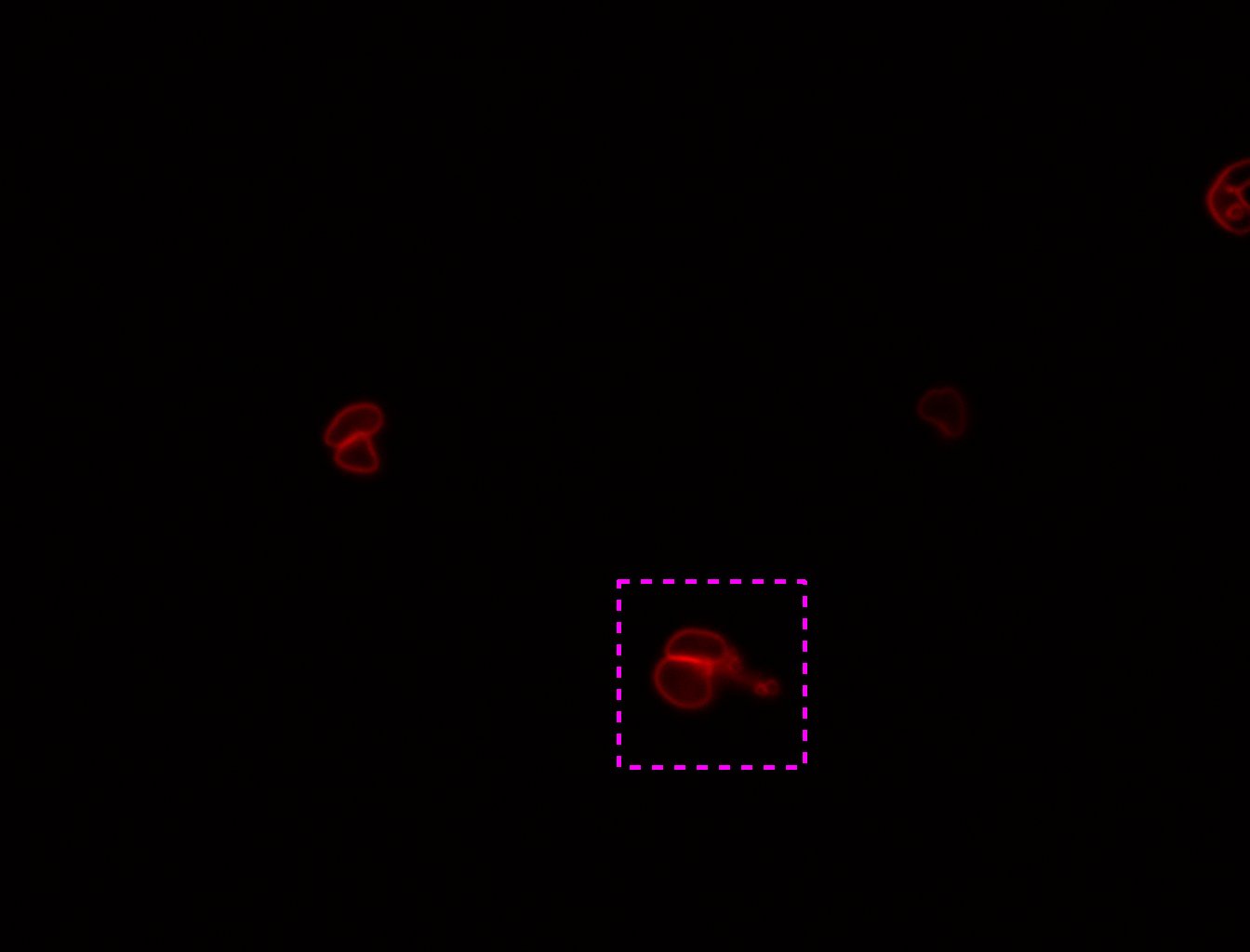

Supplement: Supplementary file 4 — Source data Fig. 3 [file 44319_2024_126_MOESM4_ESM.zip › Figure 3/3A/3A_Image_Glucose readd_Red.jpg]

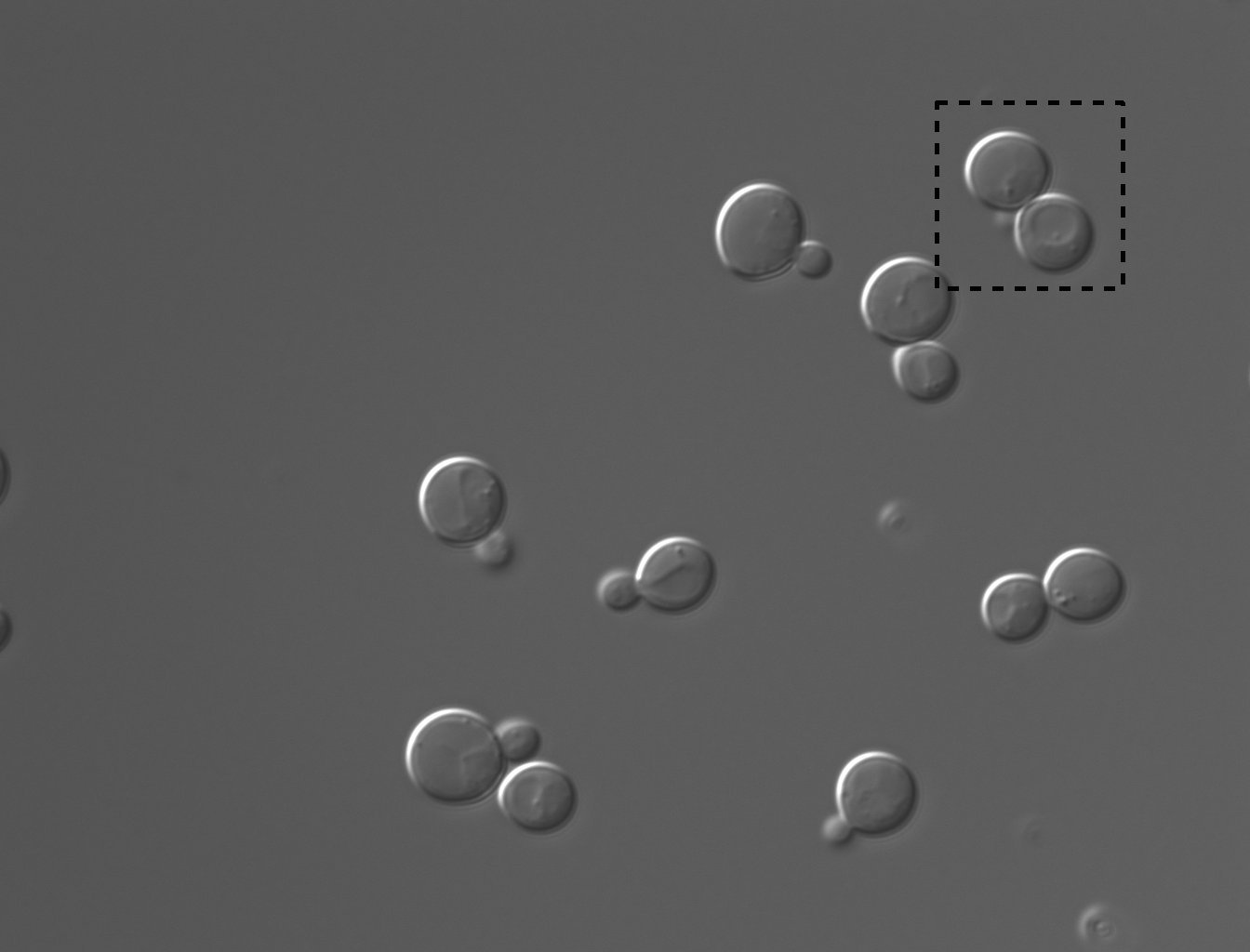

Supplement: Supplementary file 4 — Source data Fig. 3 [file 44319_2024_126_MOESM4_ESM.zip › Figure 3/3A/3A_Image_Glucose_DIC.jpg]

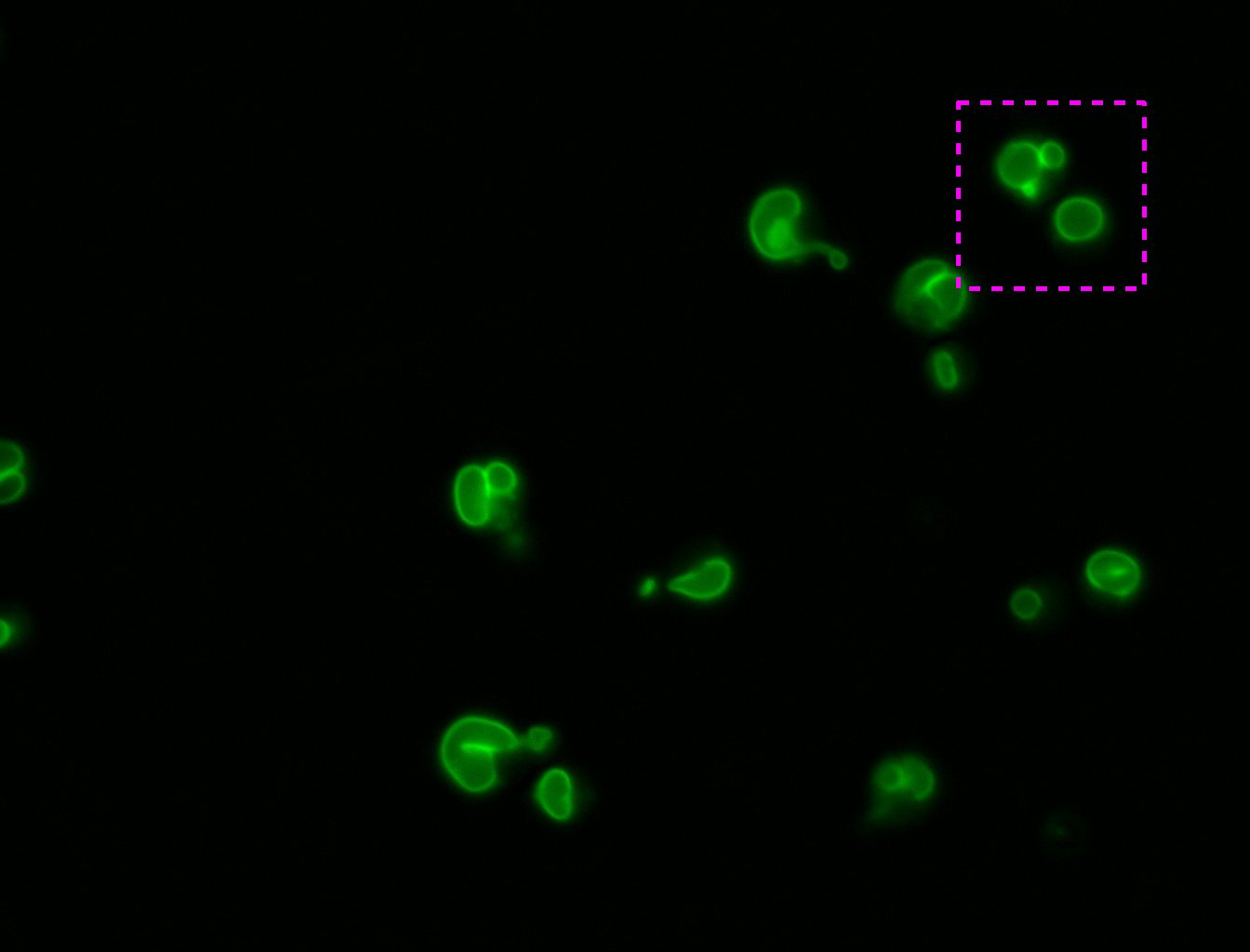

Supplement: Supplementary file 4 — Source data Fig. 3 [file 44319_2024_126_MOESM4_ESM.zip › Figure 3/3A/3A_Image_Glucose_Green.jpg]

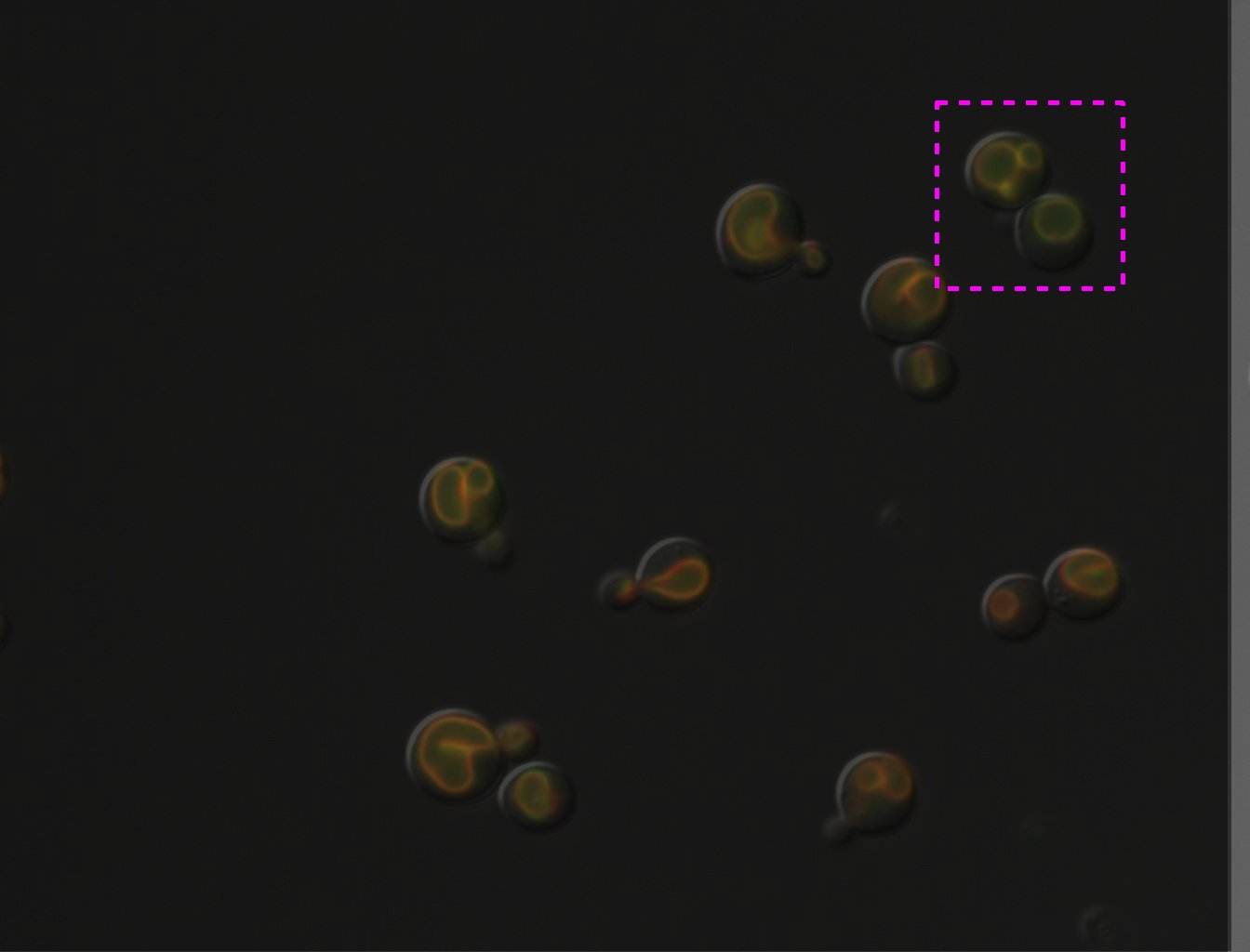

Supplement: Supplementary file 4 — Source data Fig. 3 [file 44319_2024_126_MOESM4_ESM.zip › Figure 3/3A/3A_Image_Glucose_overlay.jpg]

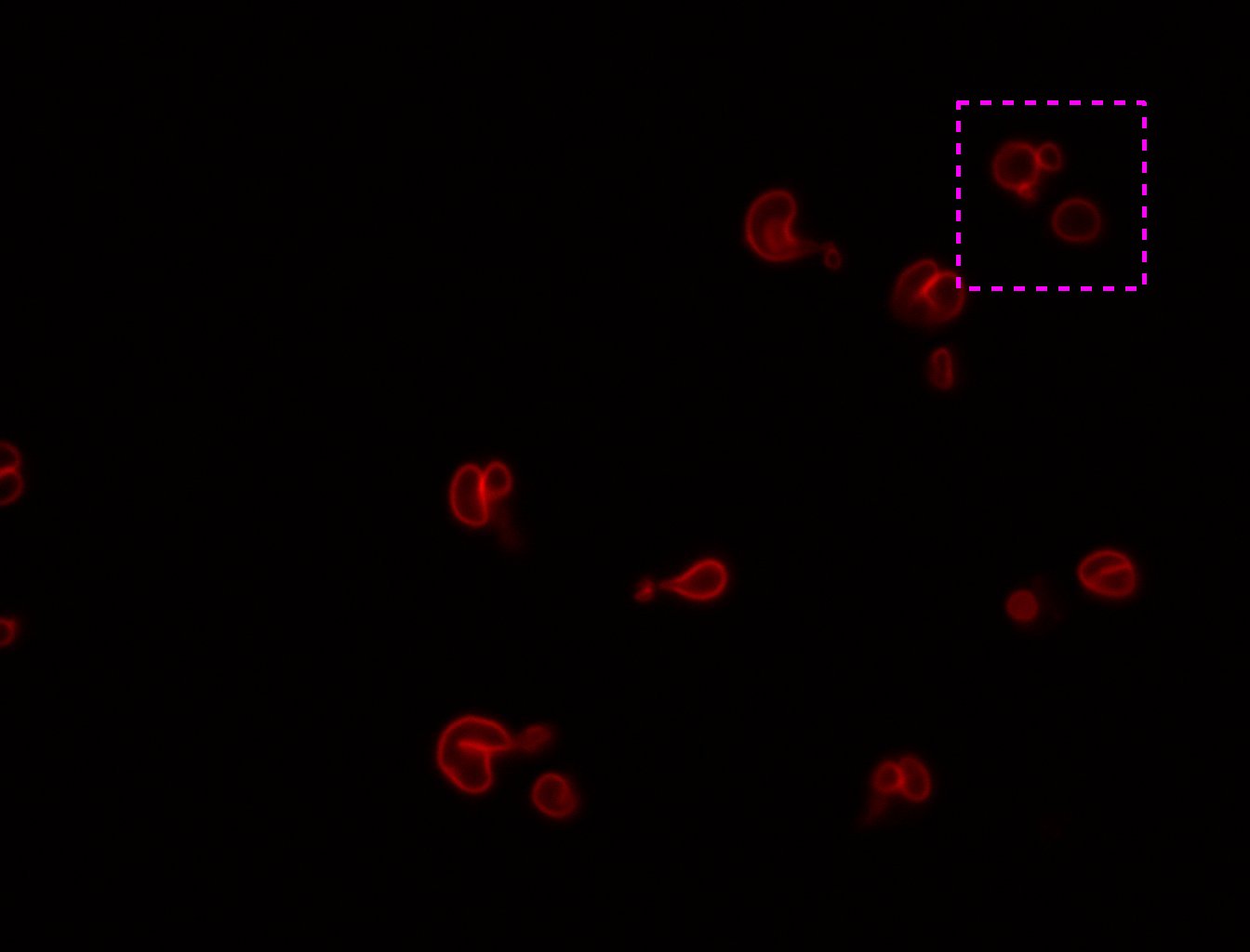

Supplement: Supplementary file 4 — Source data Fig. 3 [file 44319_2024_126_MOESM4_ESM.zip › Figure 3/3A/3A_Image_Glucose_Red.jpg]

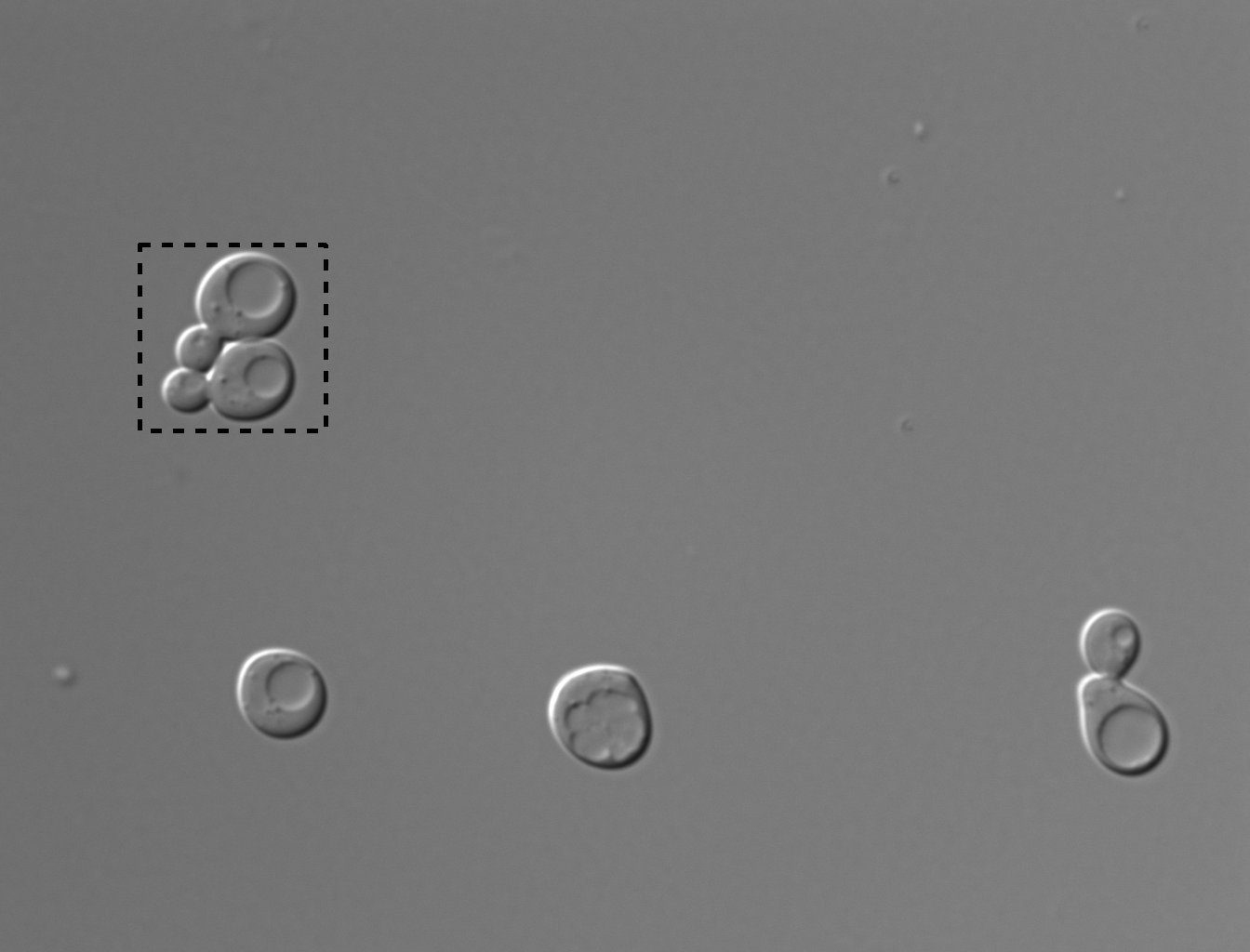

Supplement: Supplementary file 4 — Source data Fig. 3 [file 44319_2024_126_MOESM4_ESM.zip › Figure 3/3B/3B_Image_Glucose deprived_DIC.jpg]

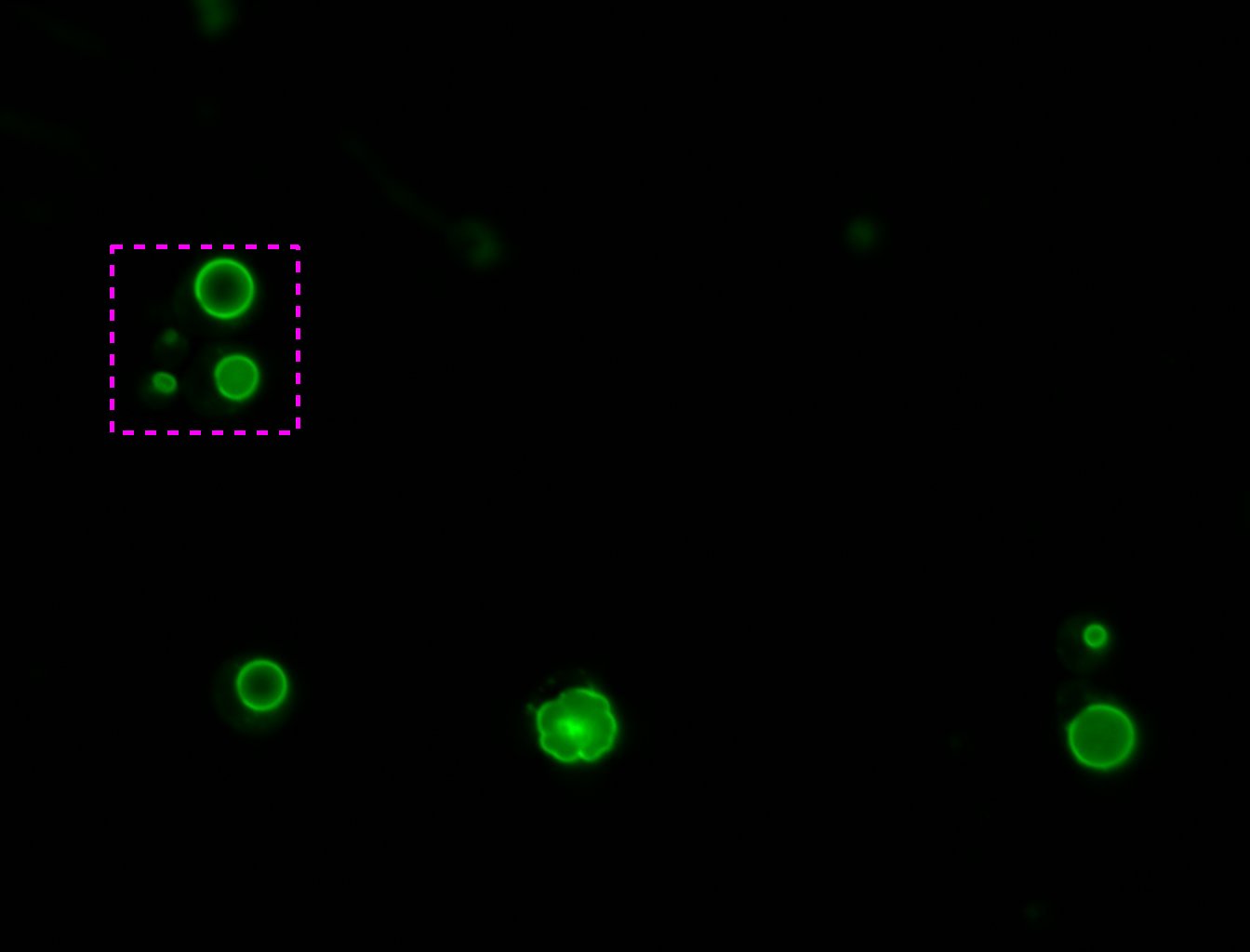

Supplement: Supplementary file 4 — Source data Fig. 3 [file 44319_2024_126_MOESM4_ESM.zip › Figure 3/3B/3B_Image_Glucose deprived_Green.jpg]

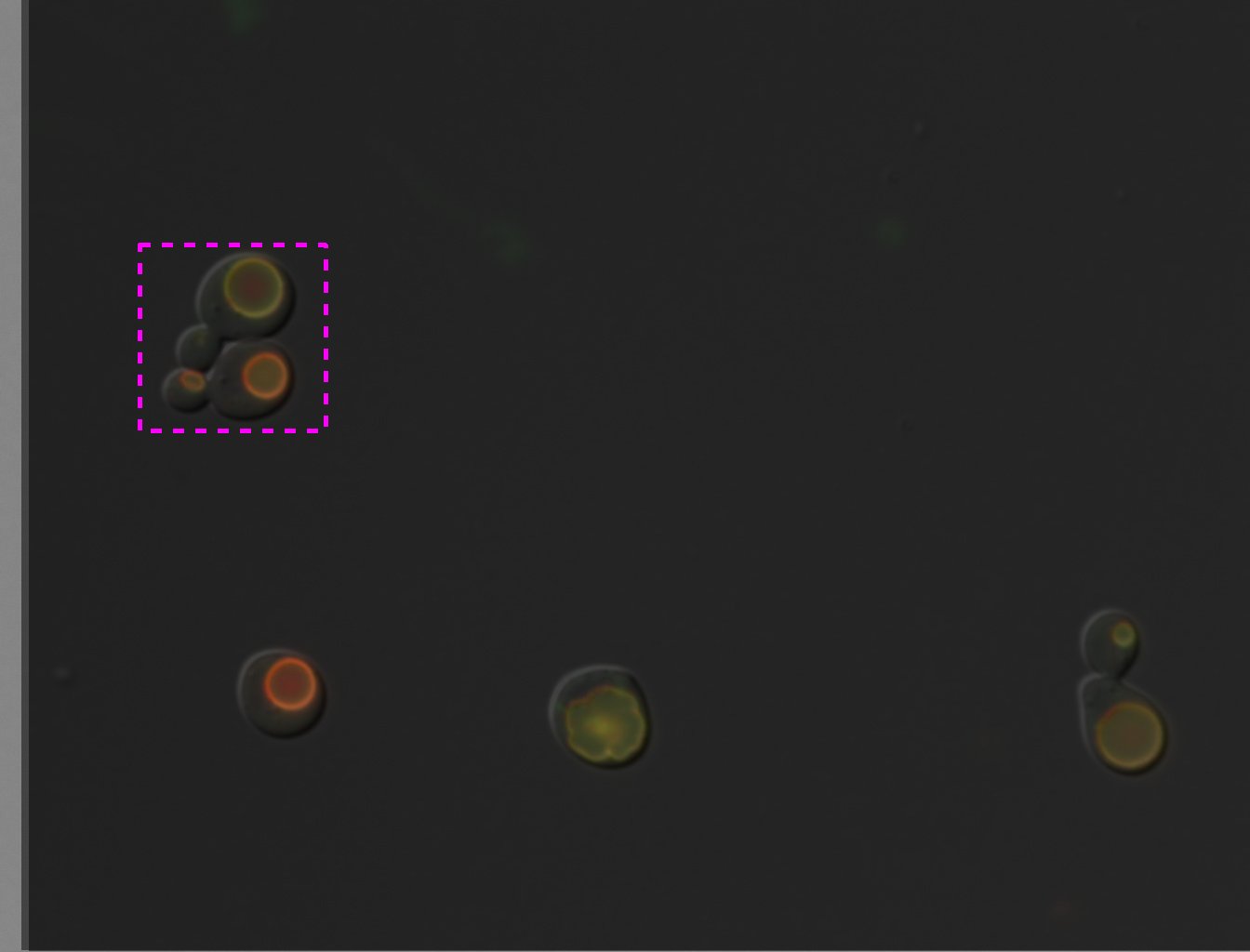

Supplement: Supplementary file 4 — Source data Fig. 3 [file 44319_2024_126_MOESM4_ESM.zip › Figure 3/3B/3B_Image_Glucose deprived_overlay.jpg]

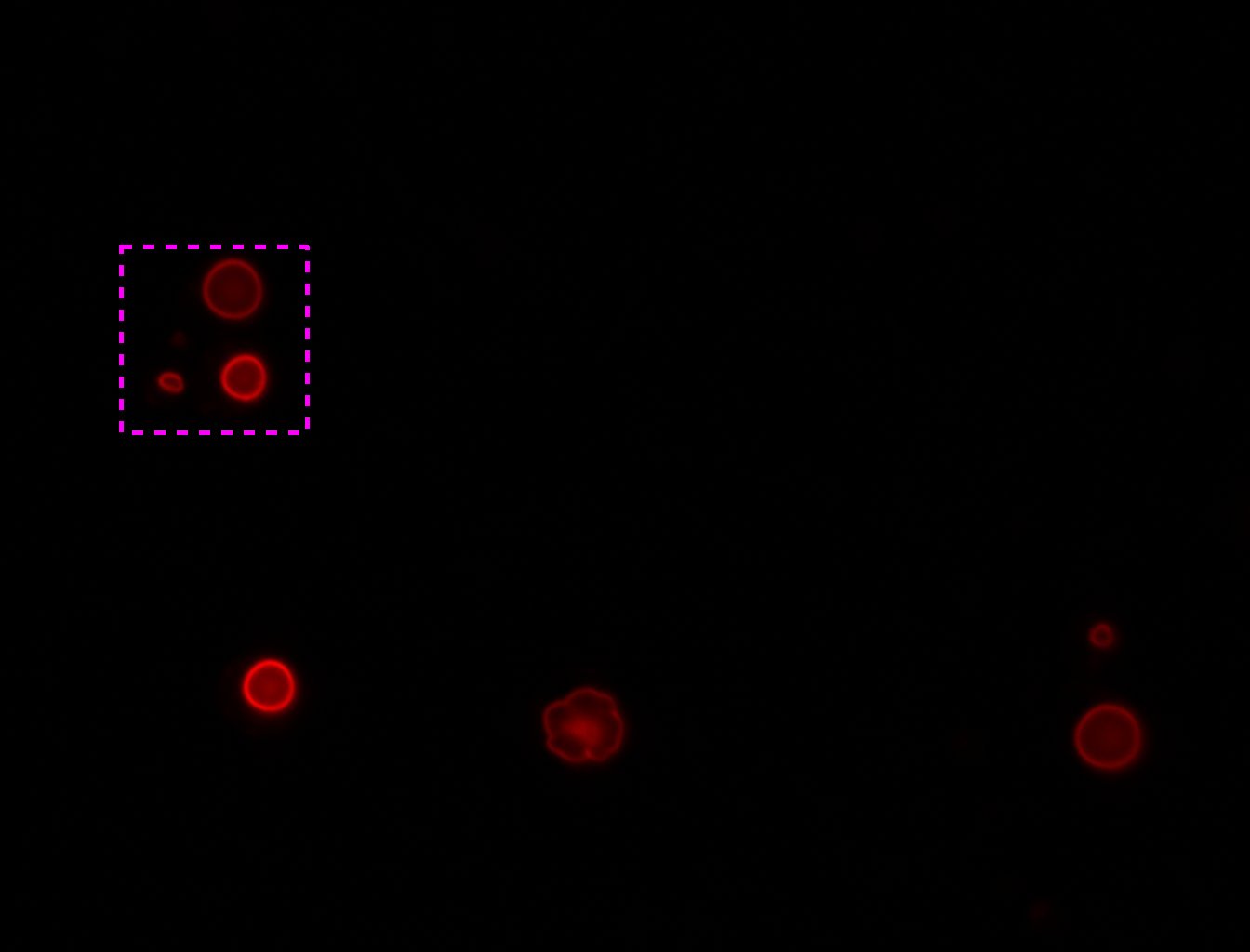

Supplement: Supplementary file 4 — Source data Fig. 3 [file 44319_2024_126_MOESM4_ESM.zip › Figure 3/3B/3B_Image_Glucose deprived_Red.jpg]

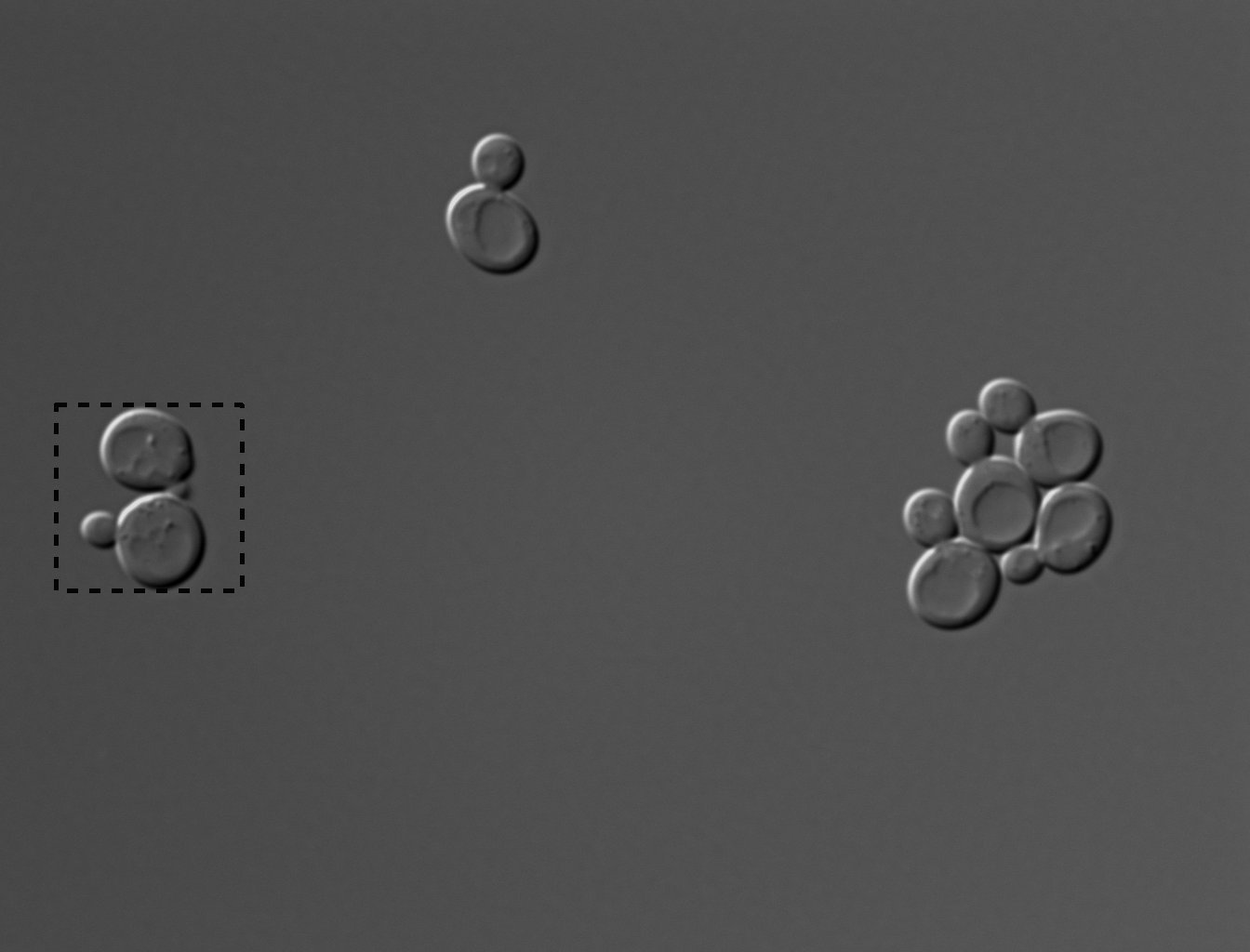

Supplement: Supplementary file 4 — Source data Fig. 3 [file 44319_2024_126_MOESM4_ESM.zip › Figure 3/3B/3B_Image_Glucose readd_DIC.jpg]

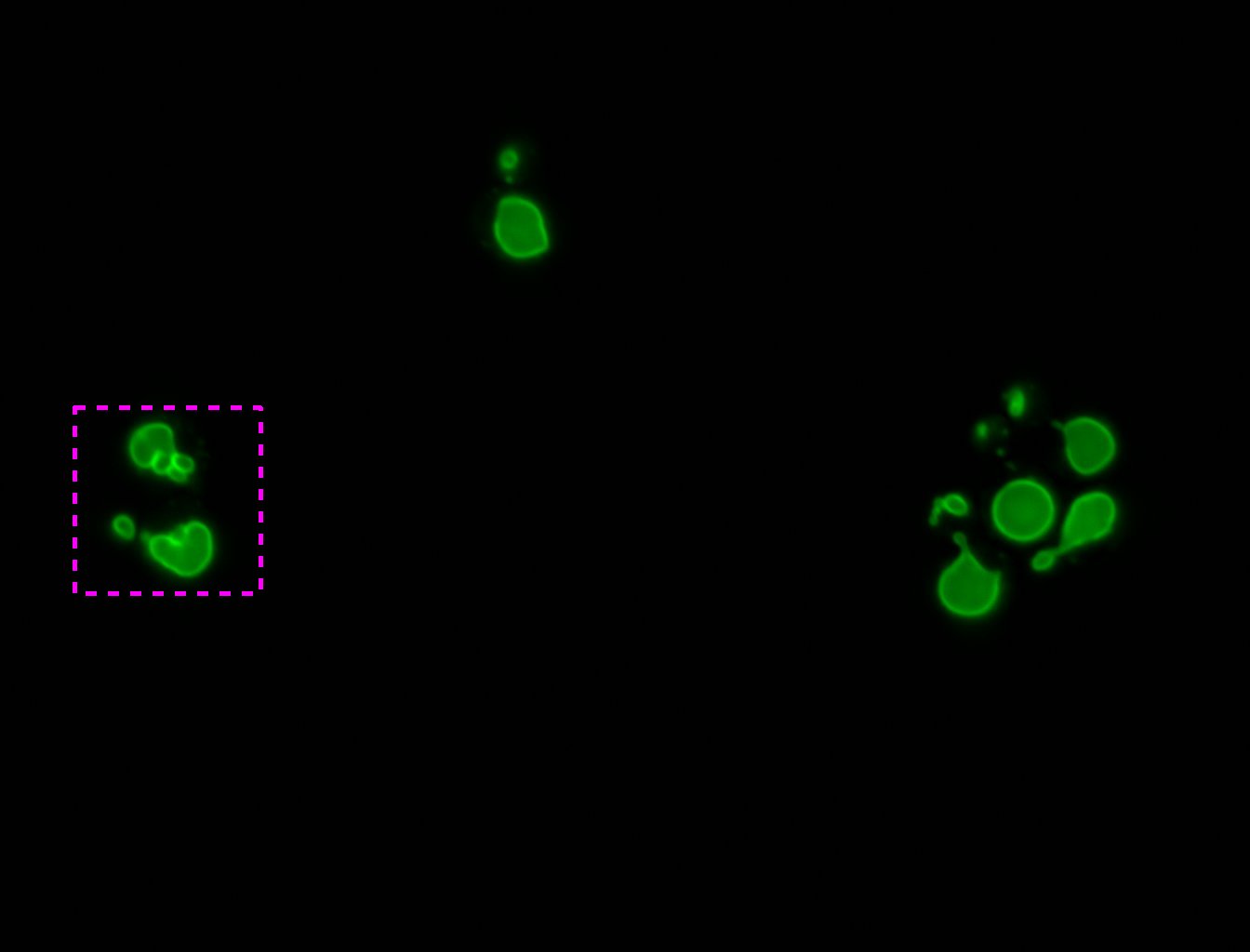

Supplement: Supplementary file 4 — Source data Fig. 3 [file 44319_2024_126_MOESM4_ESM.zip › Figure 3/3B/3B_Image_Glucose readd_Green.jpg]

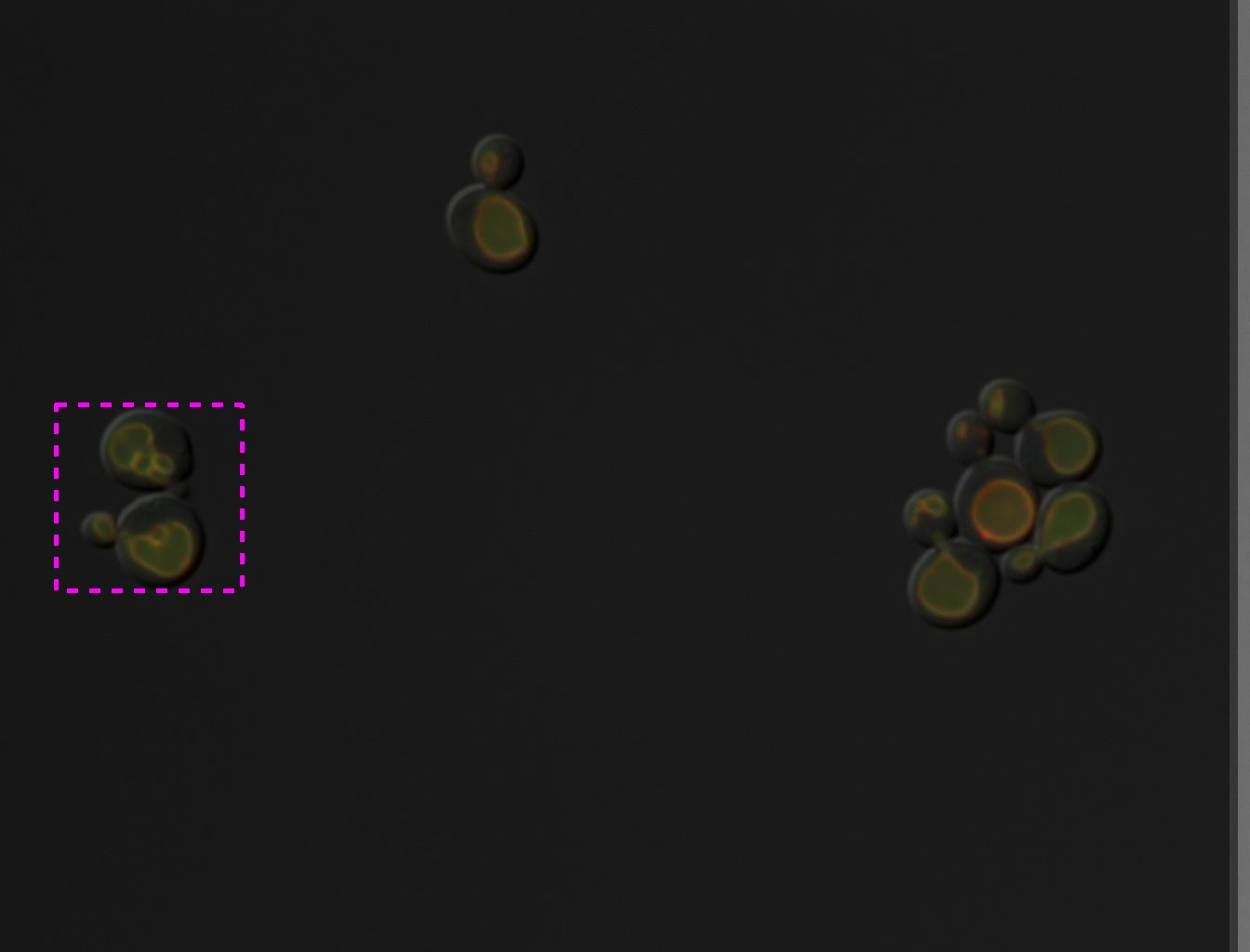

Supplement: Supplementary file 4 — Source data Fig. 3 [file 44319_2024_126_MOESM4_ESM.zip › Figure 3/3B/3B_Image_Glucose readd_overlay.jpg]

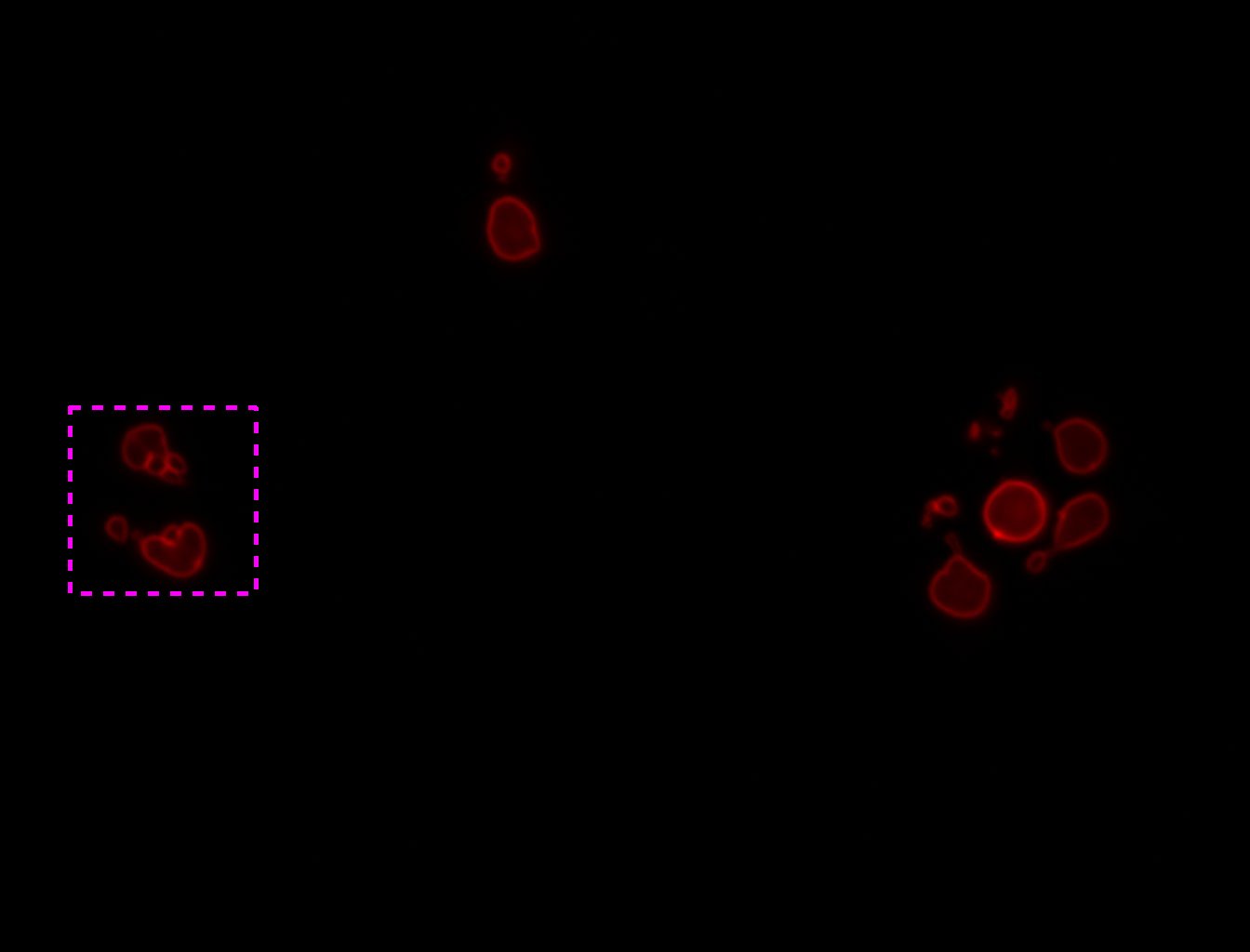

Supplement: Supplementary file 4 — Source data Fig. 3 [file 44319_2024_126_MOESM4_ESM.zip › Figure 3/3B/3B_Image_Glucose readd_Red.jpg]

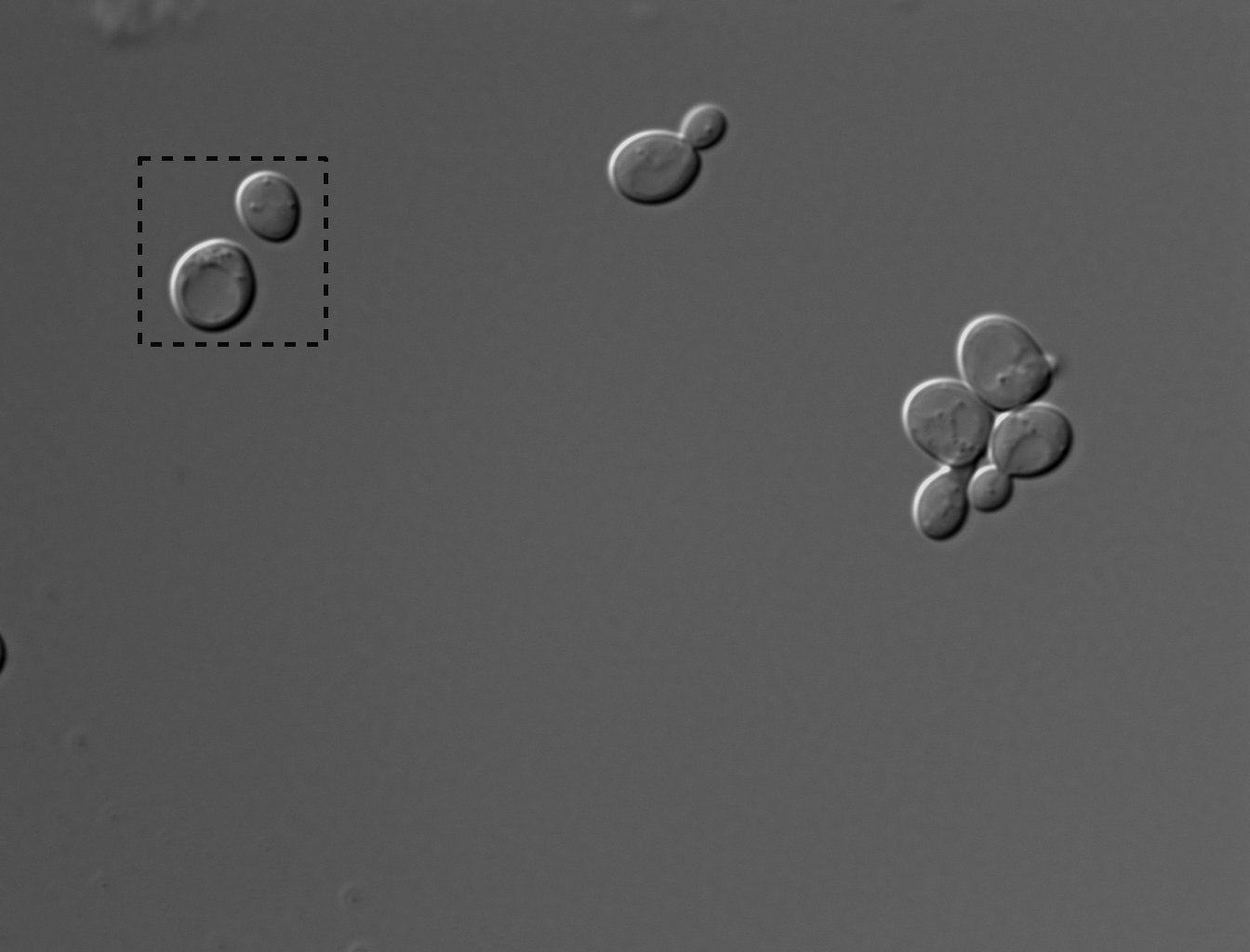

Supplement: Supplementary file 4 — Source data Fig. 3 [file 44319_2024_126_MOESM4_ESM.zip › Figure 3/3B/3B_Image_Glucose_DIC.jpg]

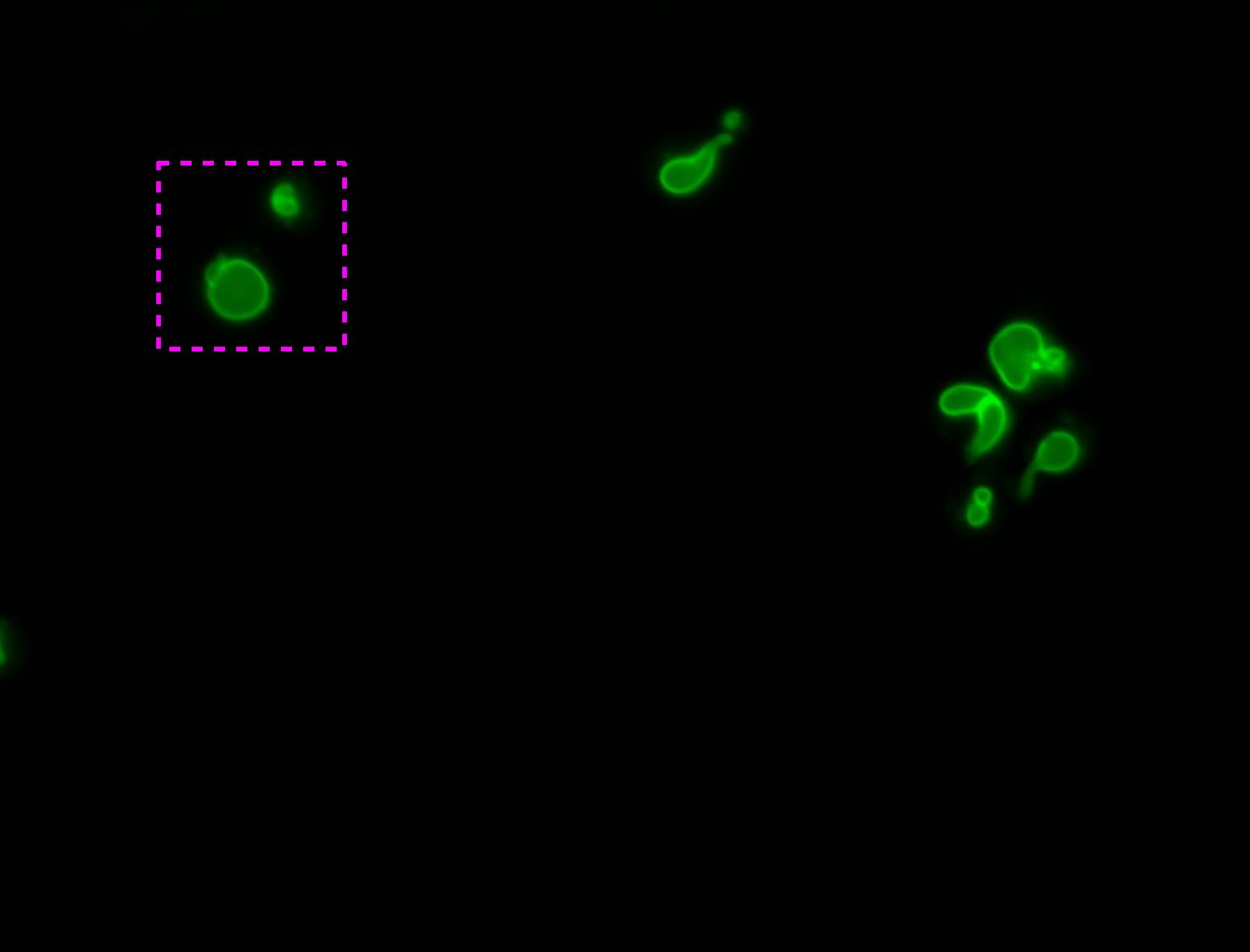

Supplement: Supplementary file 4 — Source data Fig. 3 [file 44319_2024_126_MOESM4_ESM.zip › Figure 3/3B/3B_Image_Glucose_Green.jpg]

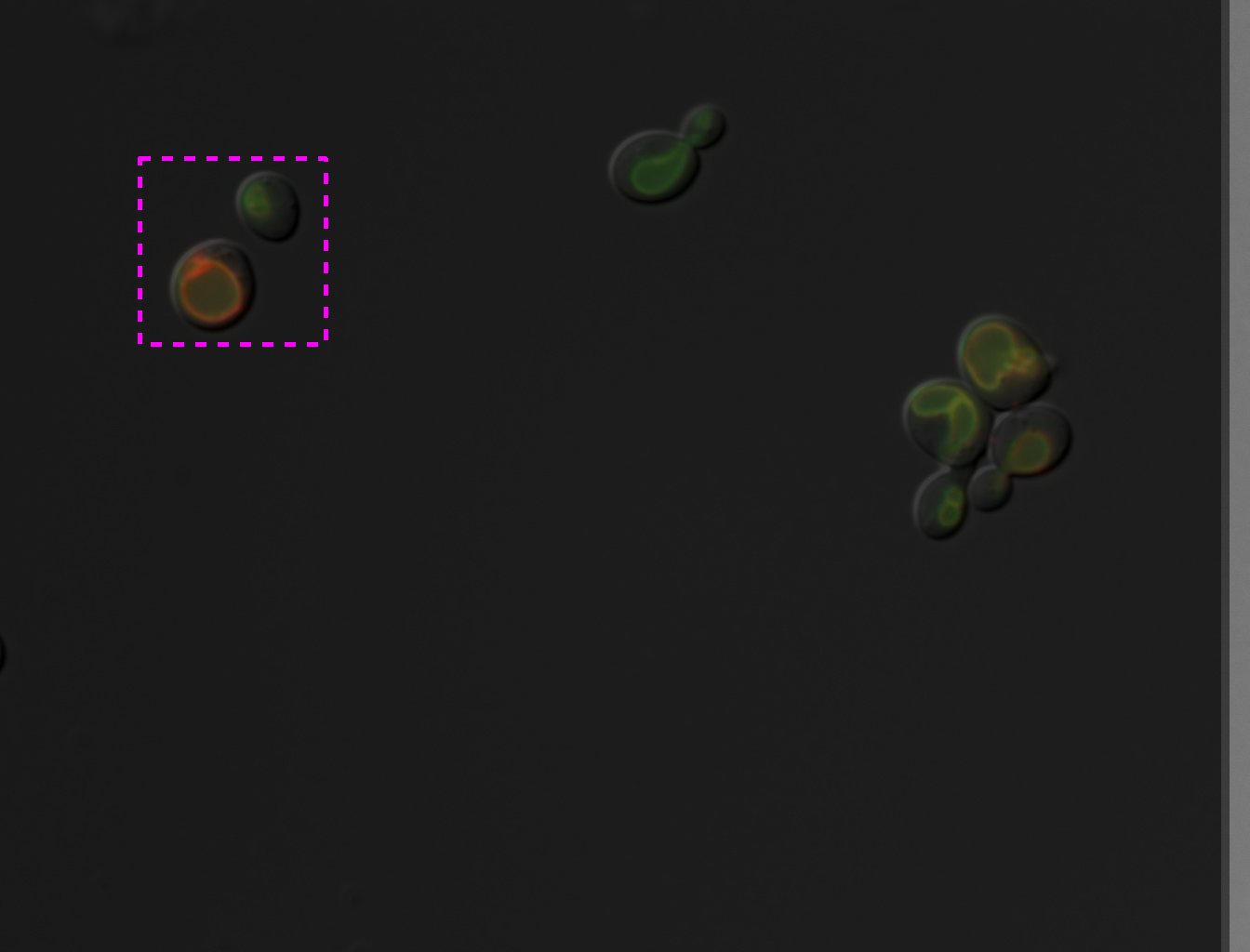

Supplement: Supplementary file 4 — Source data Fig. 3 [file 44319_2024_126_MOESM4_ESM.zip › Figure 3/3B/3B_Image_Glucose_overlay.jpg]

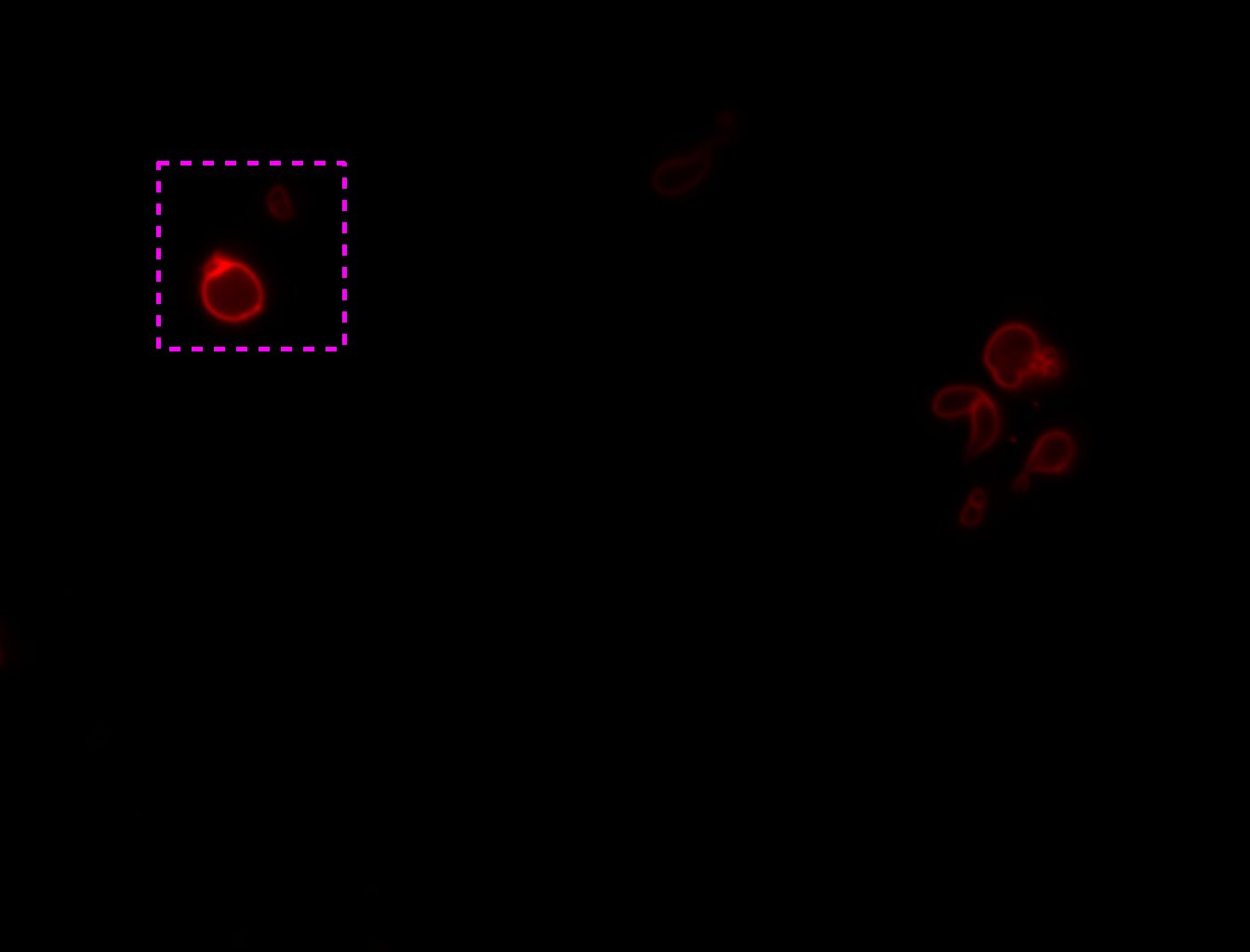

Supplement: Supplementary file 4 — Source data Fig. 3 [file 44319_2024_126_MOESM4_ESM.zip › Figure 3/3B/3B_Image_Glucose_Red.jpg]

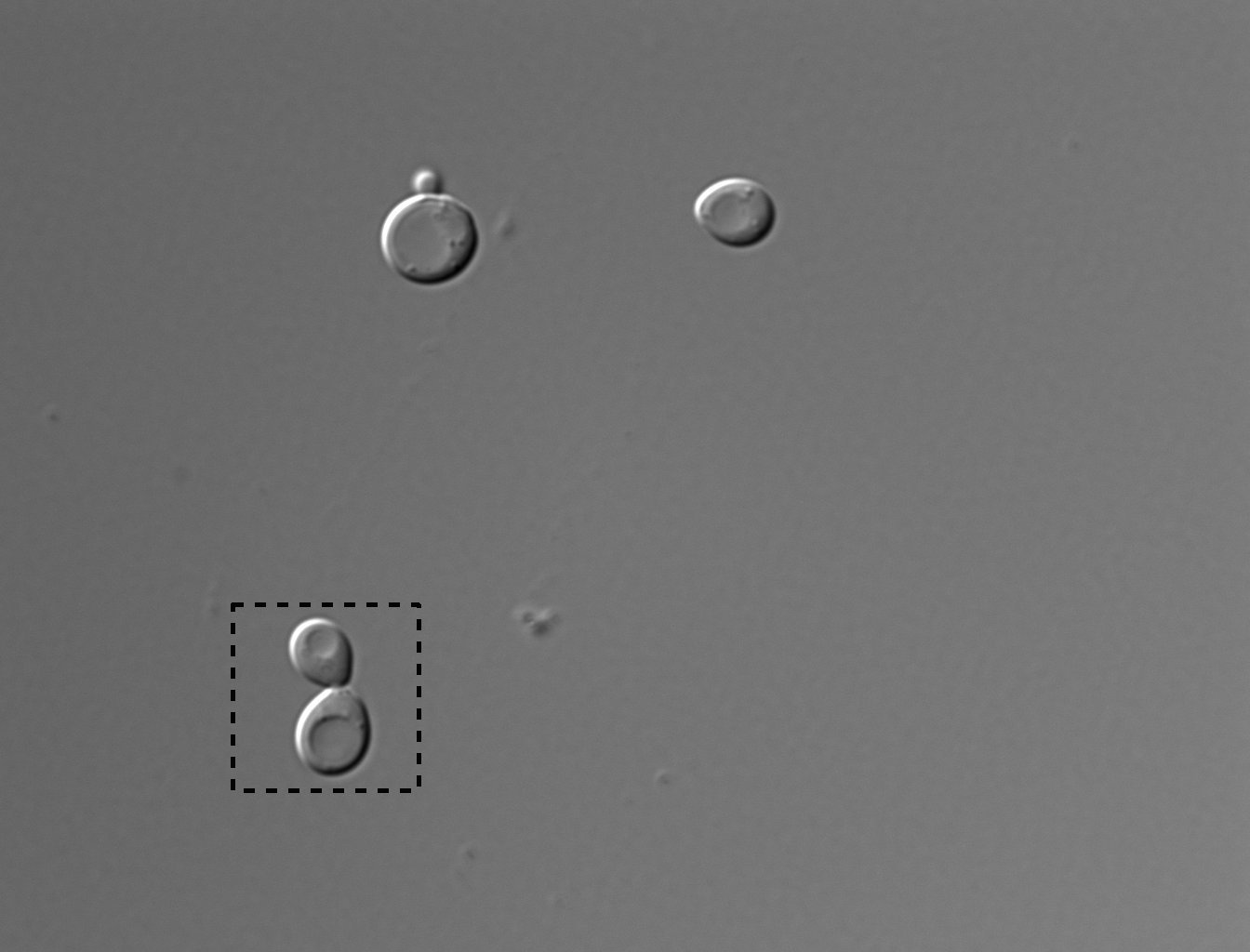

Supplement: Supplementary file 4 — Source data Fig. 3 [file 44319_2024_126_MOESM4_ESM.zip › Figure 3/3C/3C_Image_Glucose deprived_DIC.jpg]

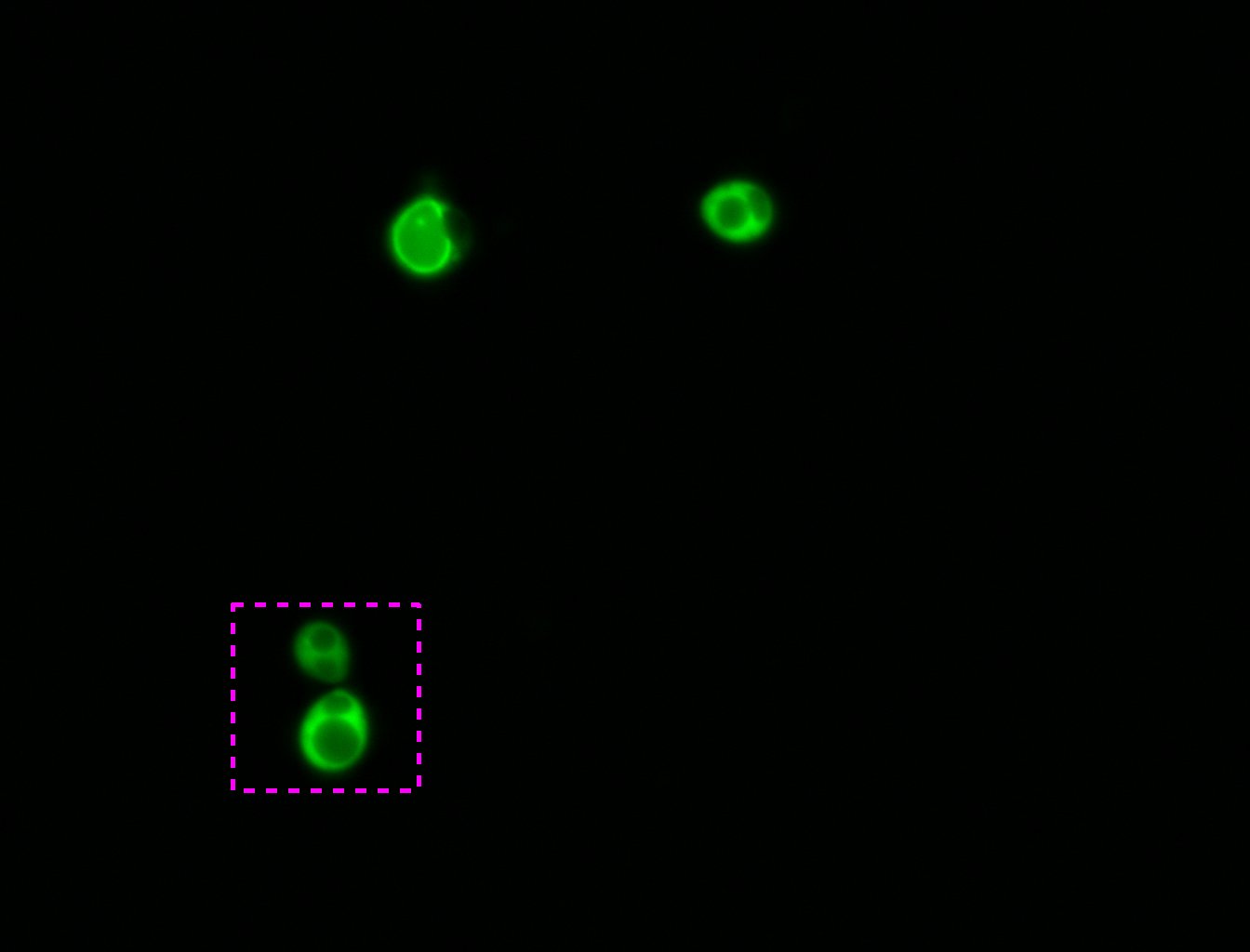

Supplement: Supplementary file 4 — Source data Fig. 3 [file 44319_2024_126_MOESM4_ESM.zip › Figure 3/3C/3C_Image_Glucose deprived_Green.jpg]

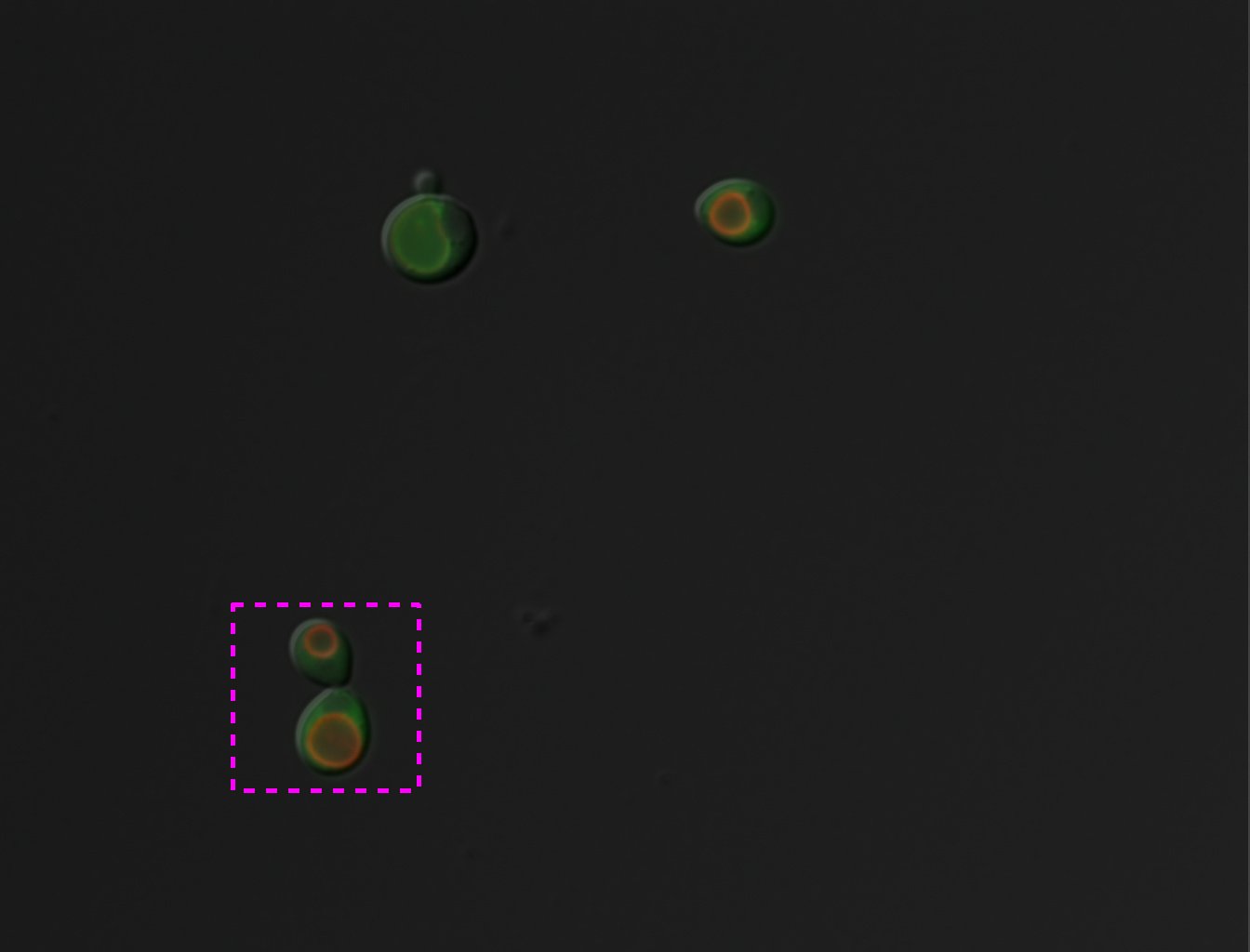

Supplement: Supplementary file 4 — Source data Fig. 3 [file 44319_2024_126_MOESM4_ESM.zip › Figure 3/3C/3C_Image_Glucose deprived_overlay.jpg]

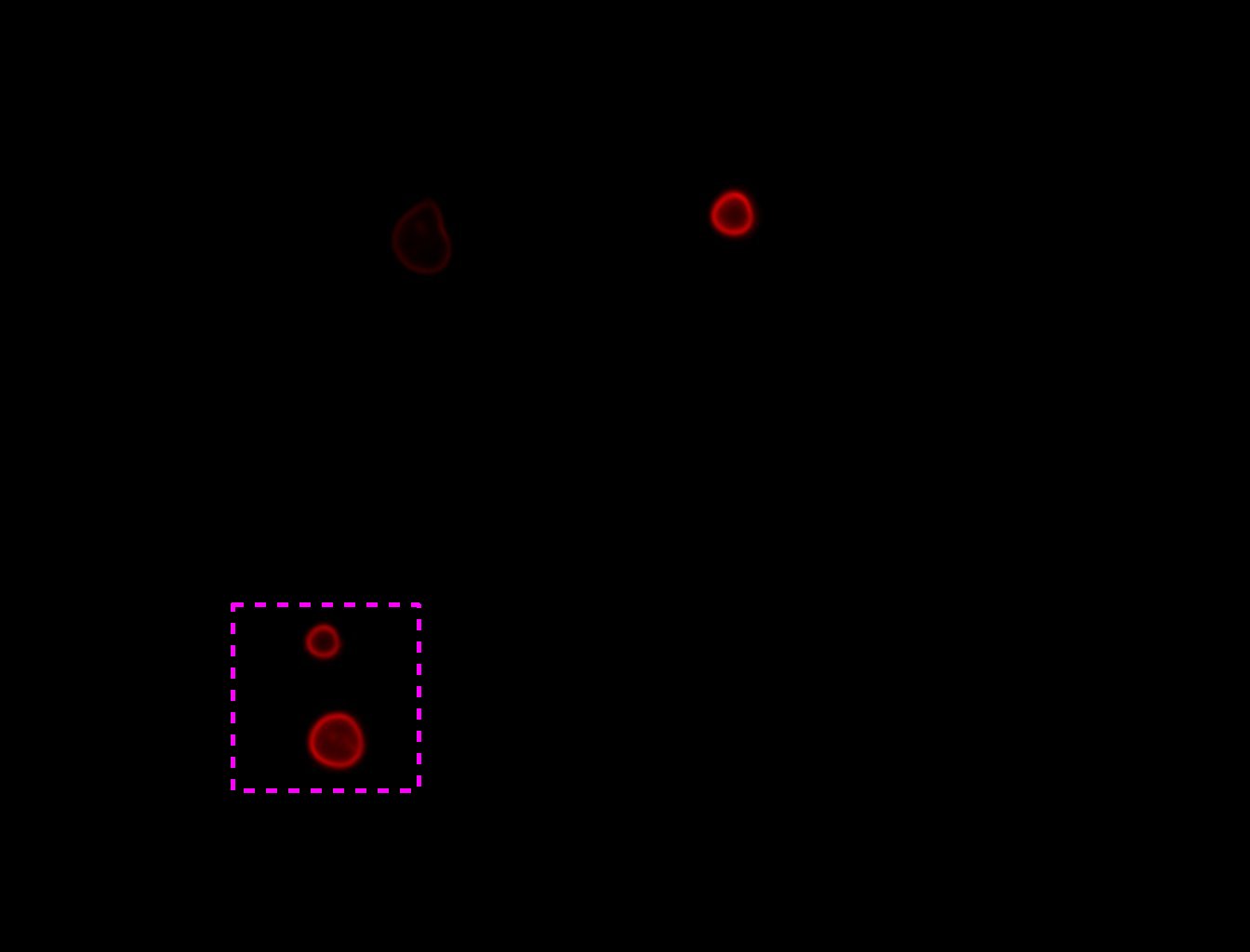

Supplement: Supplementary file 4 — Source data Fig. 3 [file 44319_2024_126_MOESM4_ESM.zip › Figure 3/3C/3C_Image_Glucose deprived_Red.jpg]

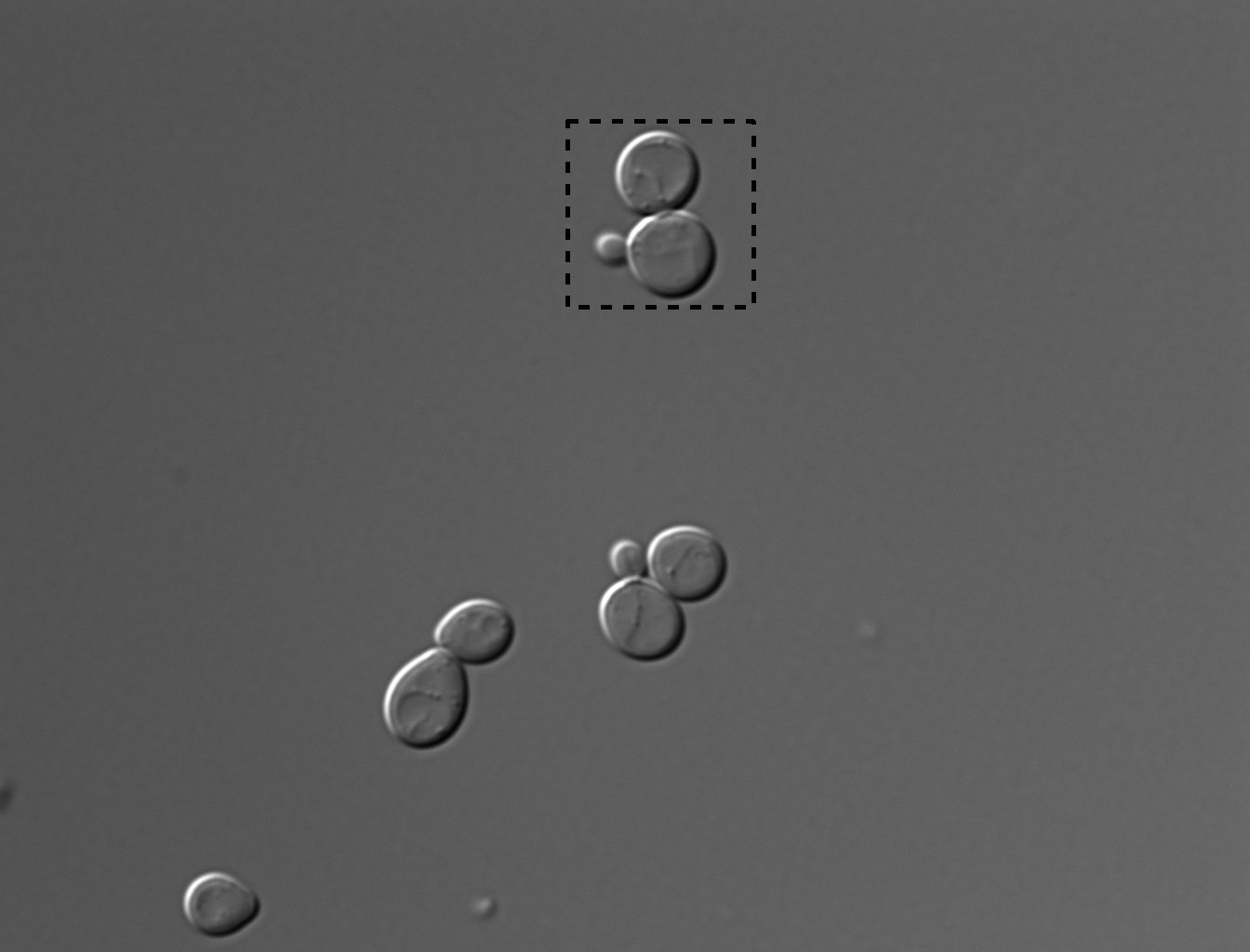

Supplement: Supplementary file 4 — Source data Fig. 3 [file 44319_2024_126_MOESM4_ESM.zip › Figure 3/3C/3C_Image_Glucose readd_DIC.jpg]

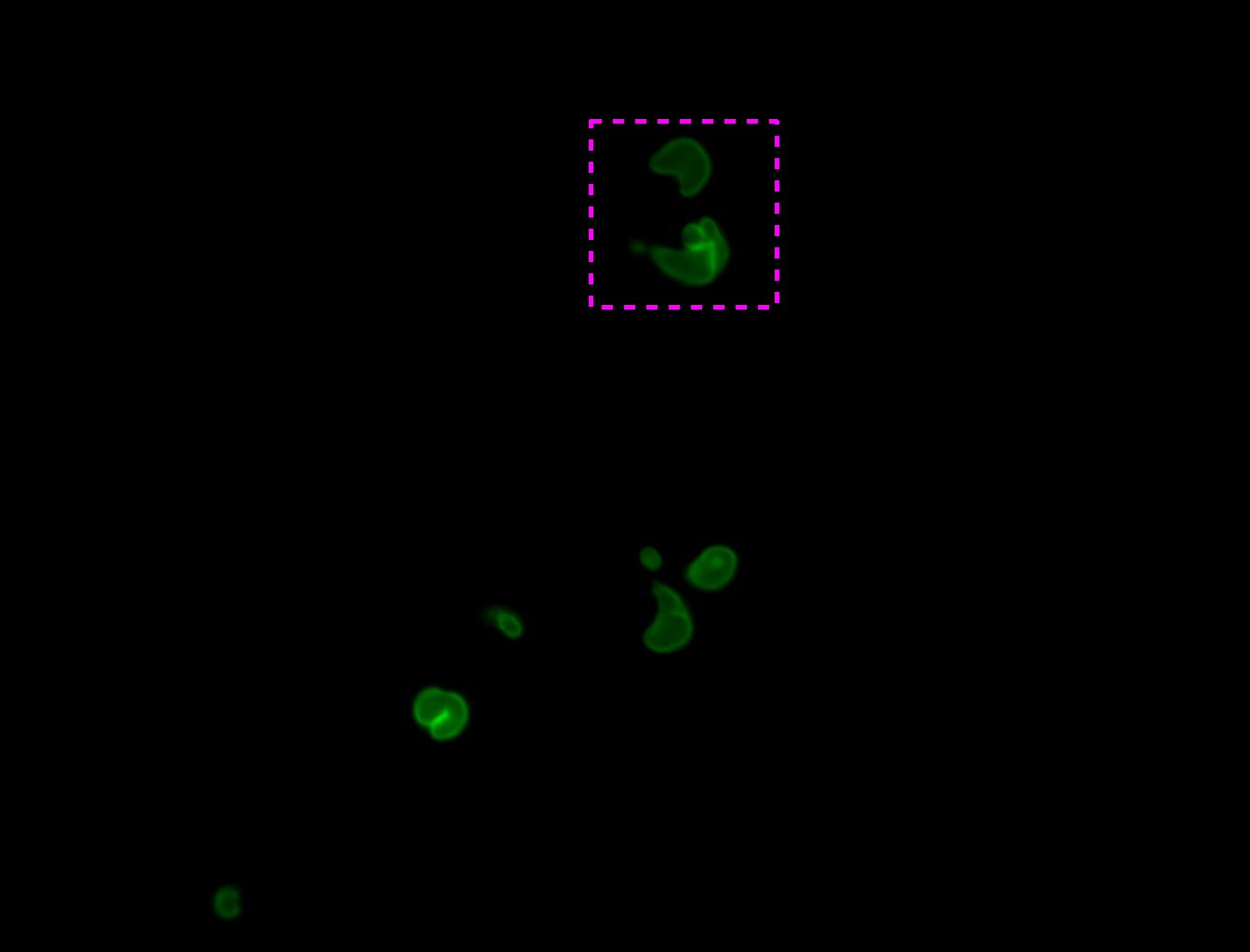

Supplement: Supplementary file 4 — Source data Fig. 3 [file 44319_2024_126_MOESM4_ESM.zip › Figure 3/3C/3C_Image_Glucose readd_Green.jpg]

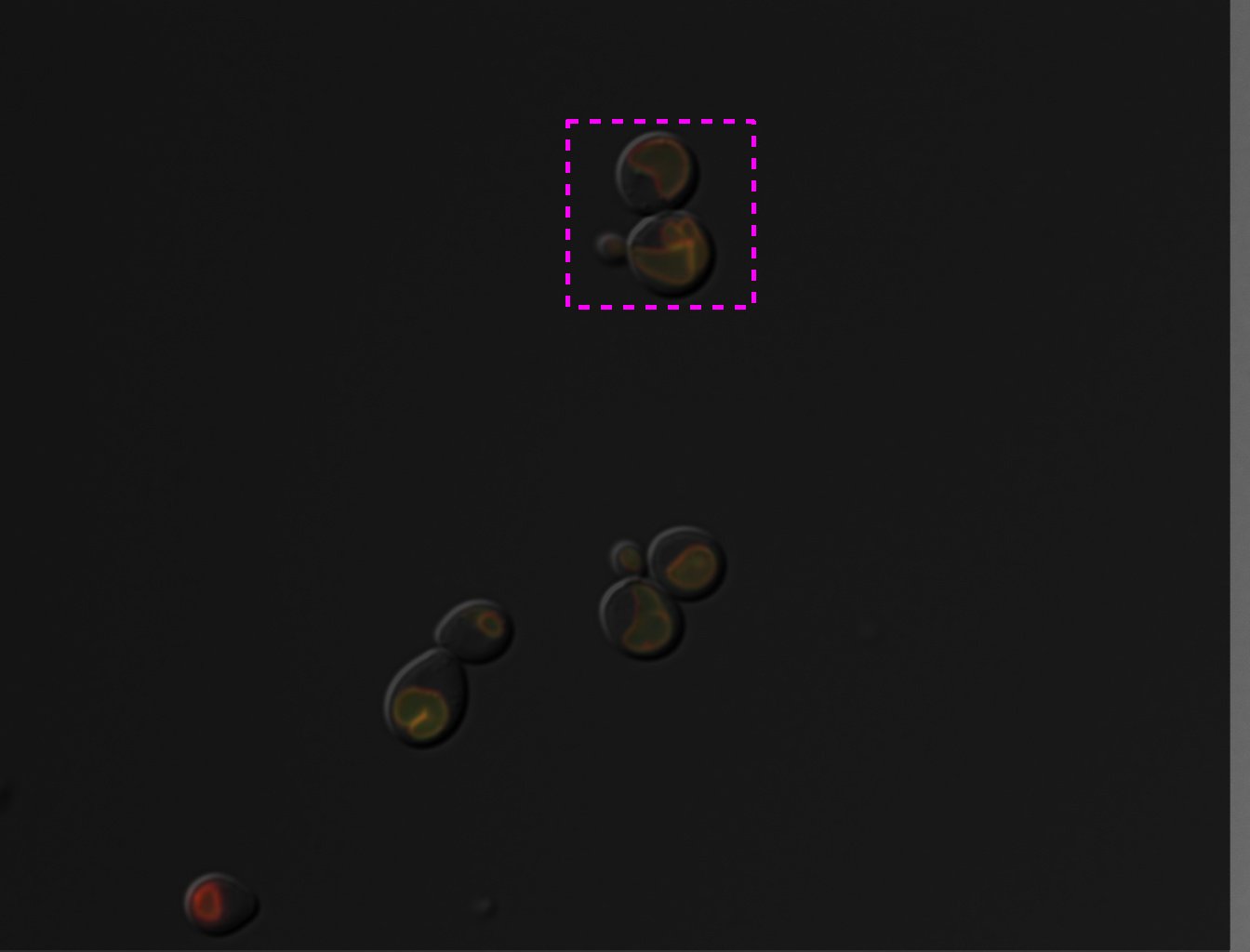

Supplement: Supplementary file 4 — Source data Fig. 3 [file 44319_2024_126_MOESM4_ESM.zip › Figure 3/3C/3C_Image_Glucose readd_overlay.jpg]

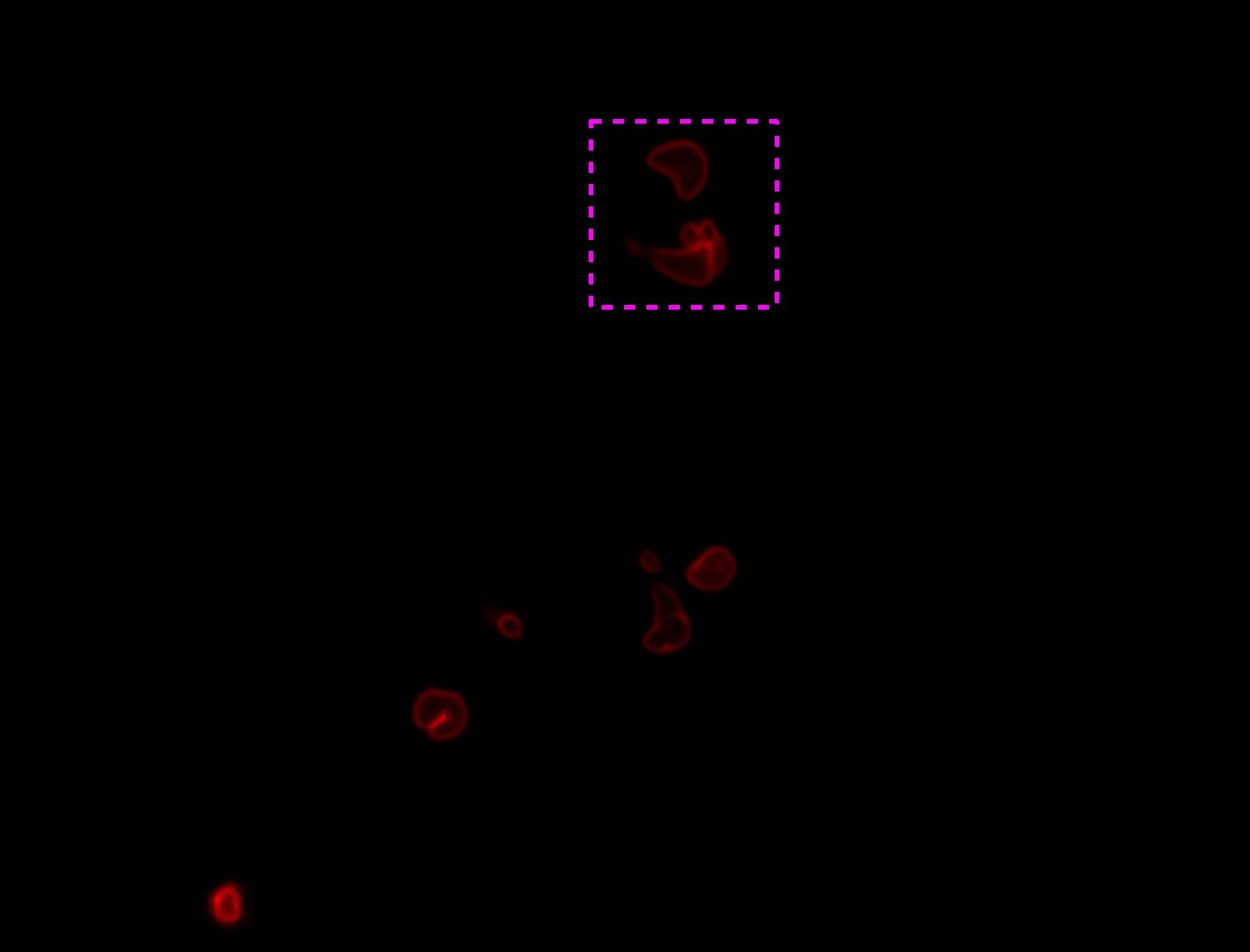

Supplement: Supplementary file 4 — Source data Fig. 3 [file 44319_2024_126_MOESM4_ESM.zip › Figure 3/3C/3C_Image_Glucose readd_Red.jpg]

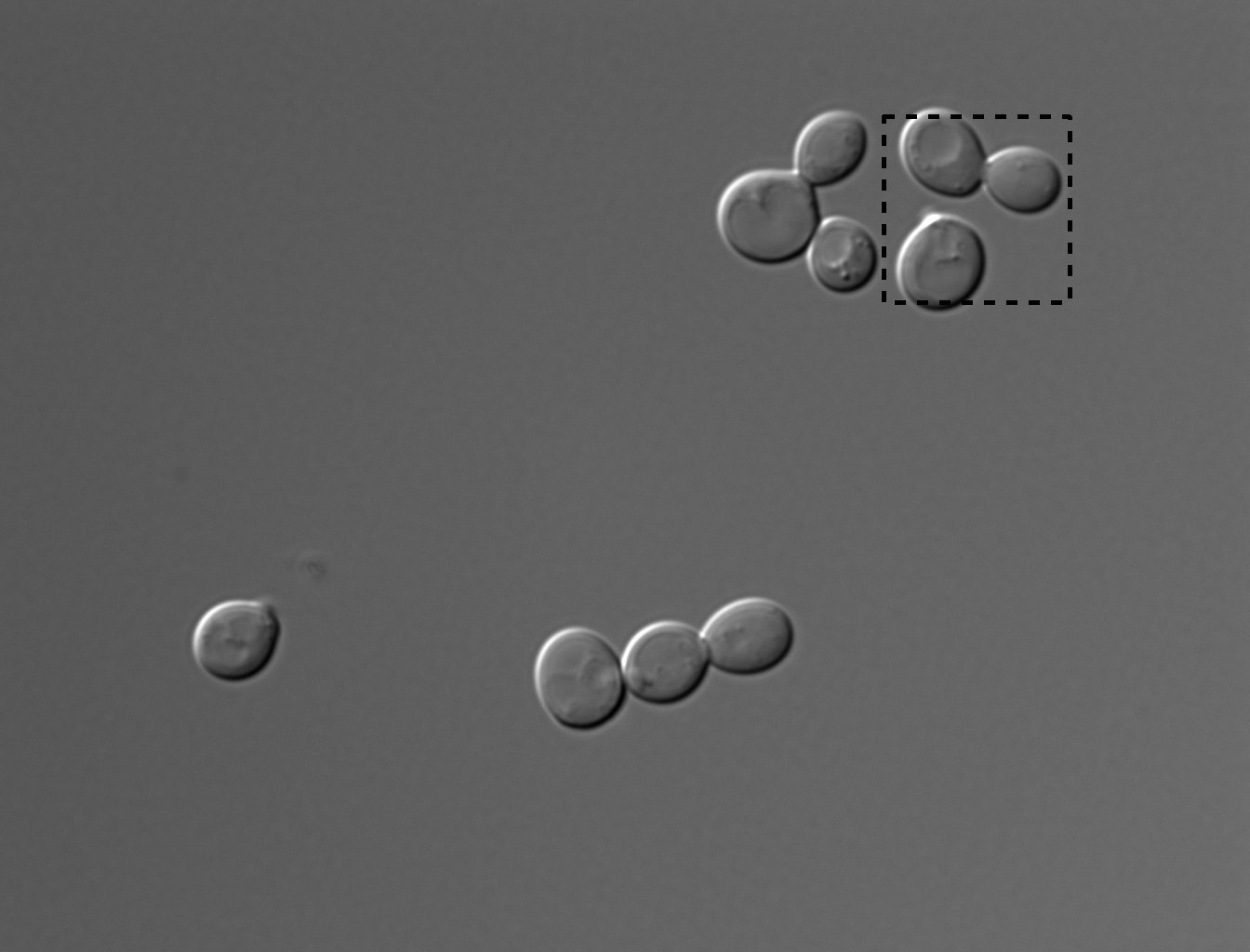

Supplement: Supplementary file 4 — Source data Fig. 3 [file 44319_2024_126_MOESM4_ESM.zip › Figure 3/3C/3C_Image_Glucose_DIC.jpg]

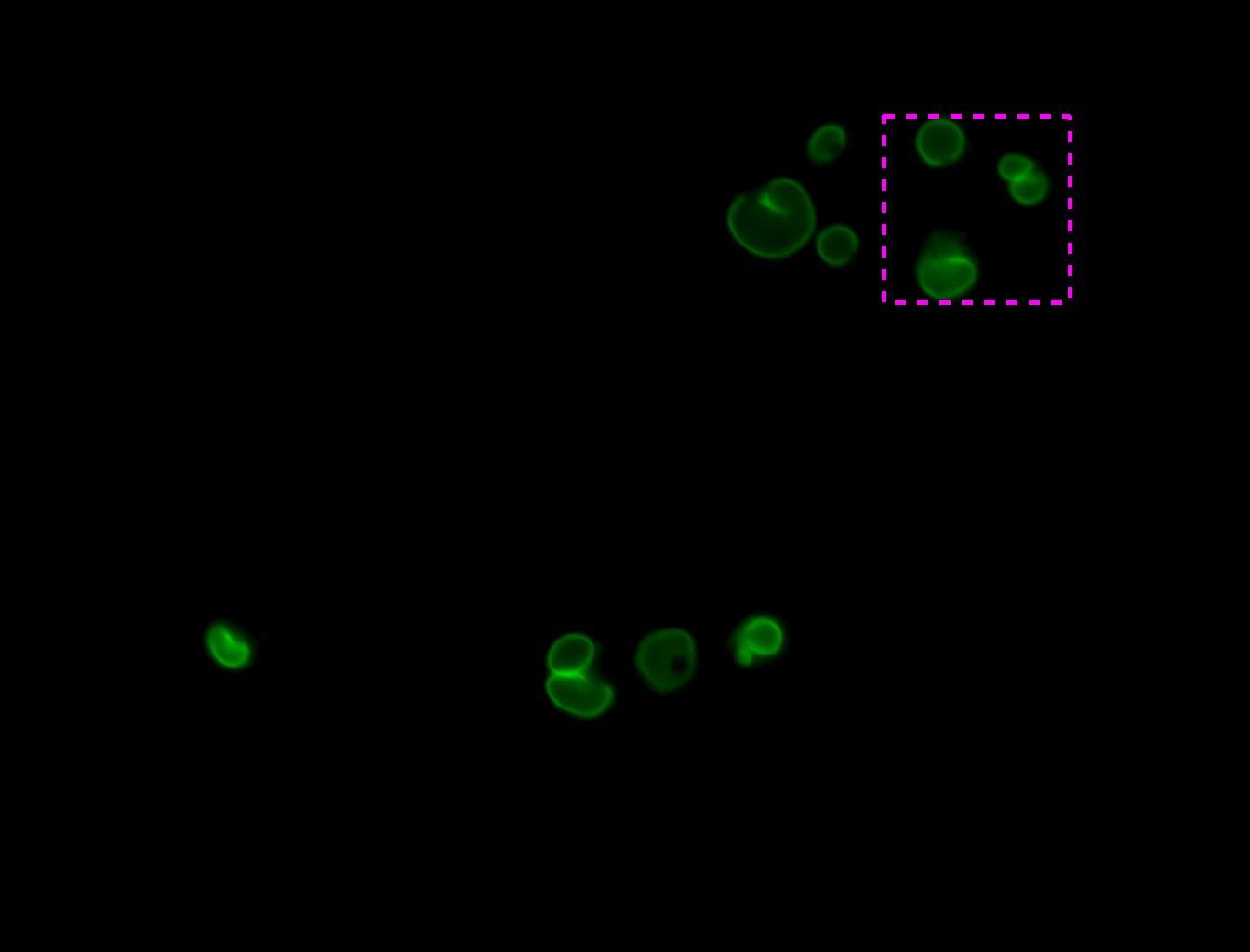

Supplement: Supplementary file 4 — Source data Fig. 3 [file 44319_2024_126_MOESM4_ESM.zip › Figure 3/3C/3C_Image_Glucose_Green.jpg]

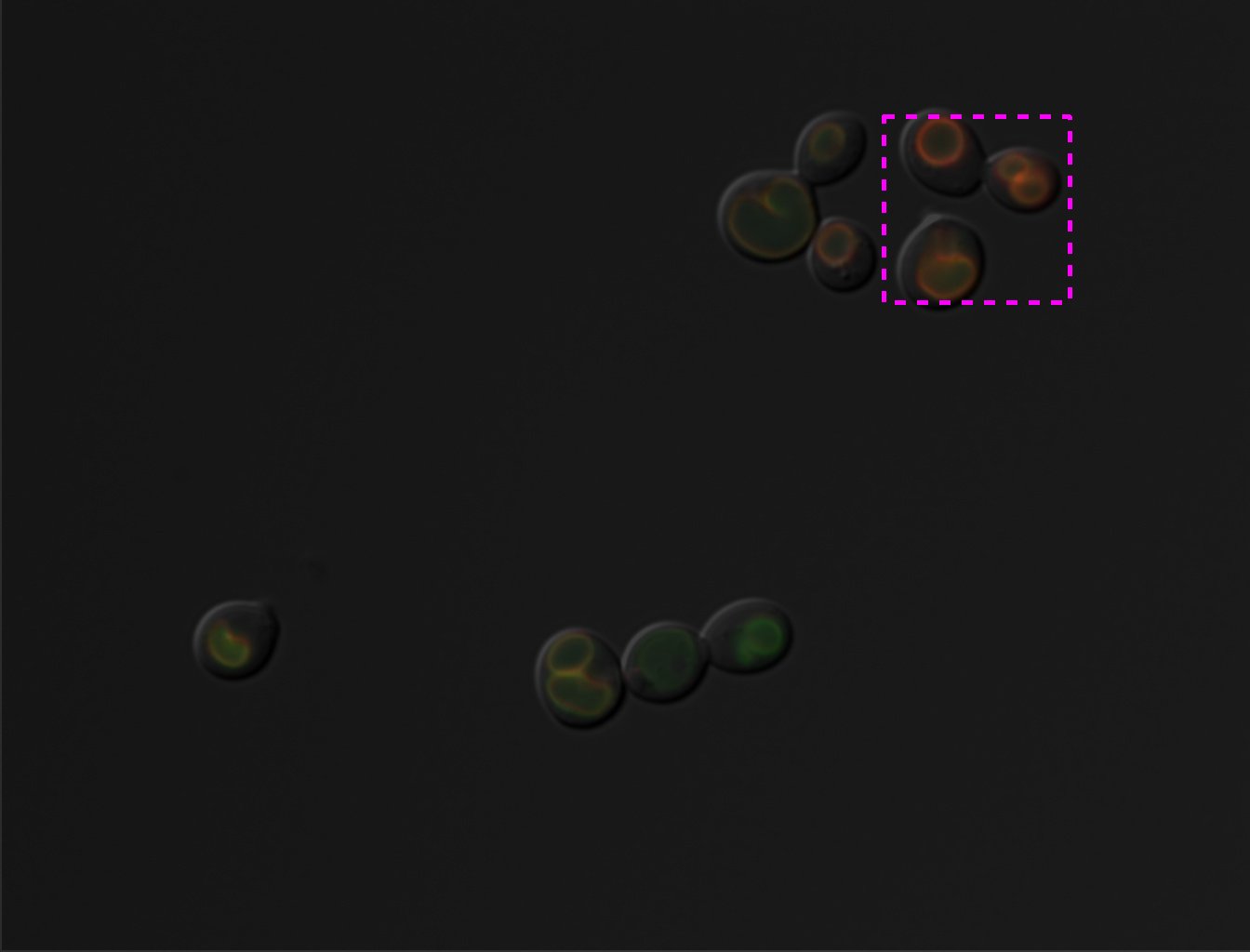

Supplement: Supplementary file 4 — Source data Fig. 3 [file 44319_2024_126_MOESM4_ESM.zip › Figure 3/3C/3C_Image_Glucose_overlay.jpg]

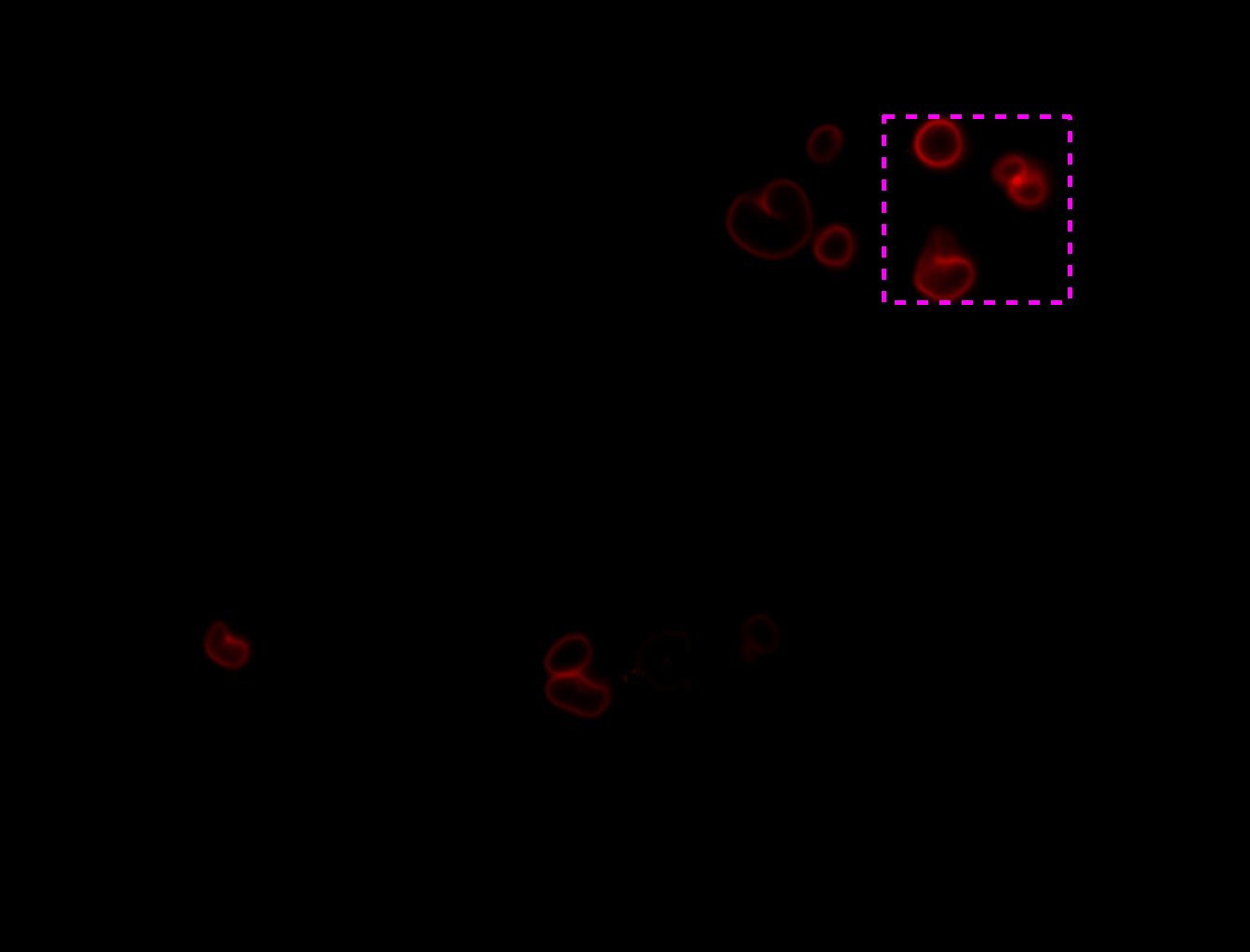

Supplement: Supplementary file 4 — Source data Fig. 3 [file 44319_2024_126_MOESM4_ESM.zip › Figure 3/3C/3C_Image_Glucose_Red.jpg]

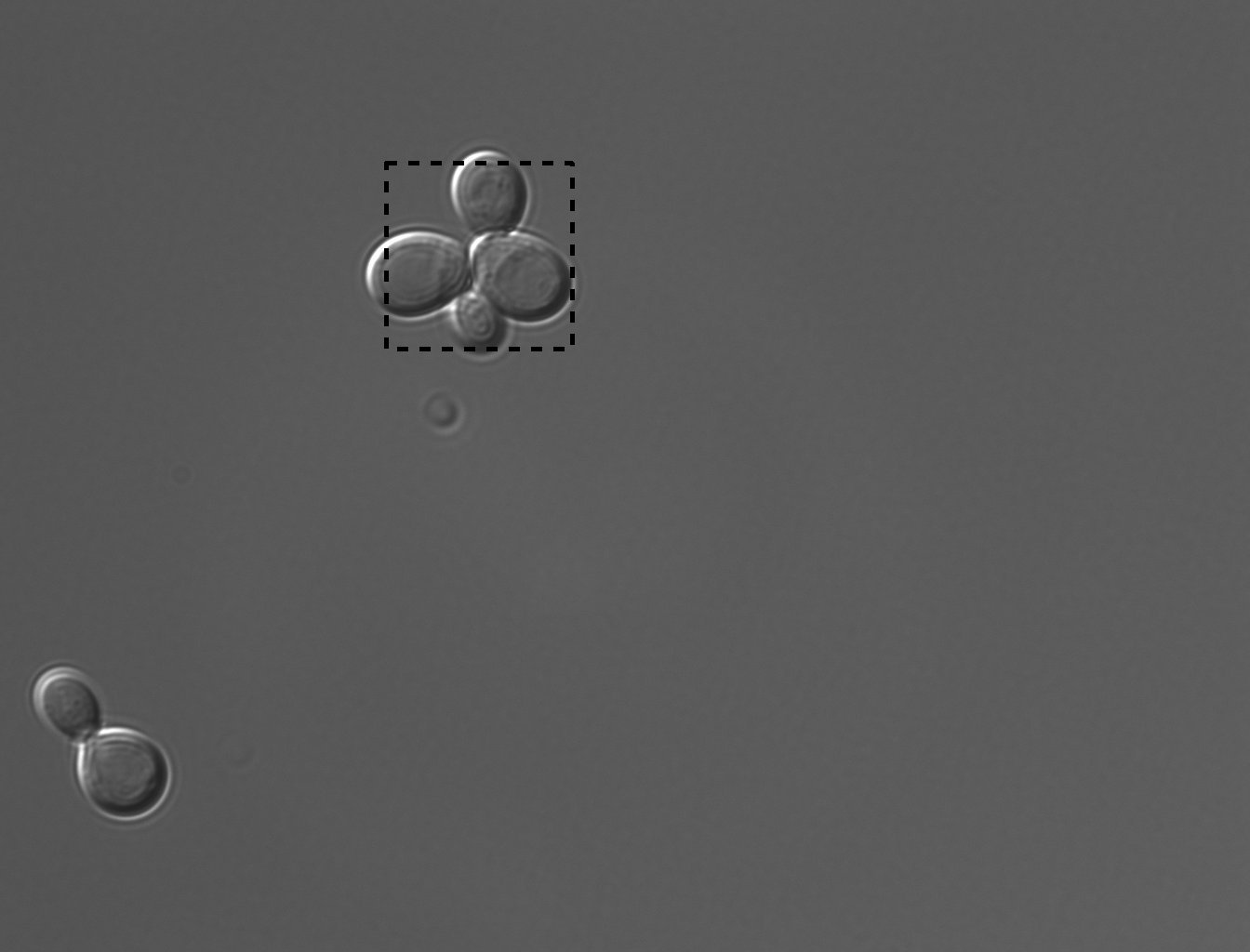

Supplement: Supplementary file 5 — Source data Fig. 4 [file 44319_2024_126_MOESM5_ESM.zip › Figure 4/4A/4A_Image_mNG-Oxr1_DIC.jpg]

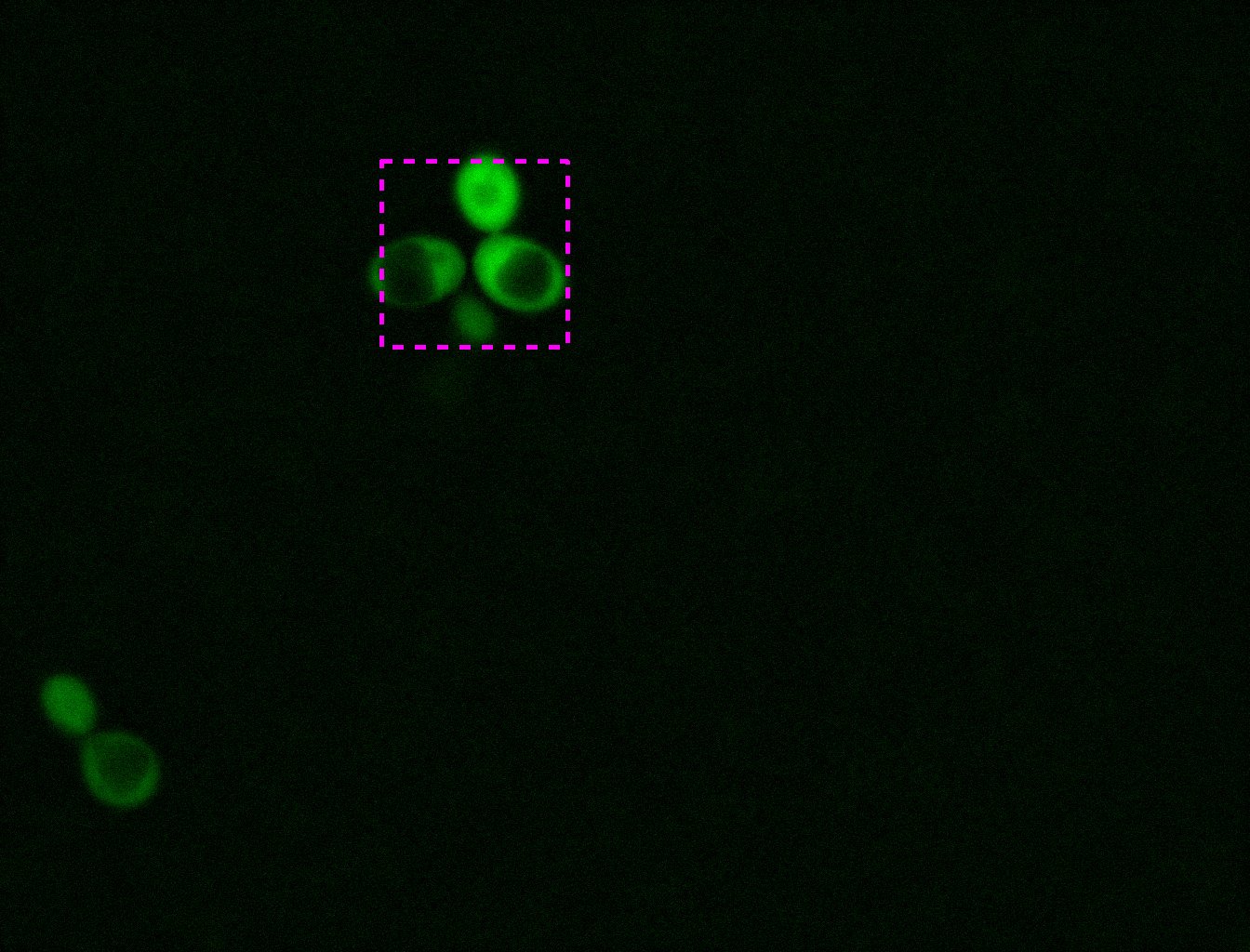

Supplement: Supplementary file 5 — Source data Fig. 4 [file 44319_2024_126_MOESM5_ESM.zip › Figure 4/4A/4A_Image_mNG-Oxr1_Green.jpg]

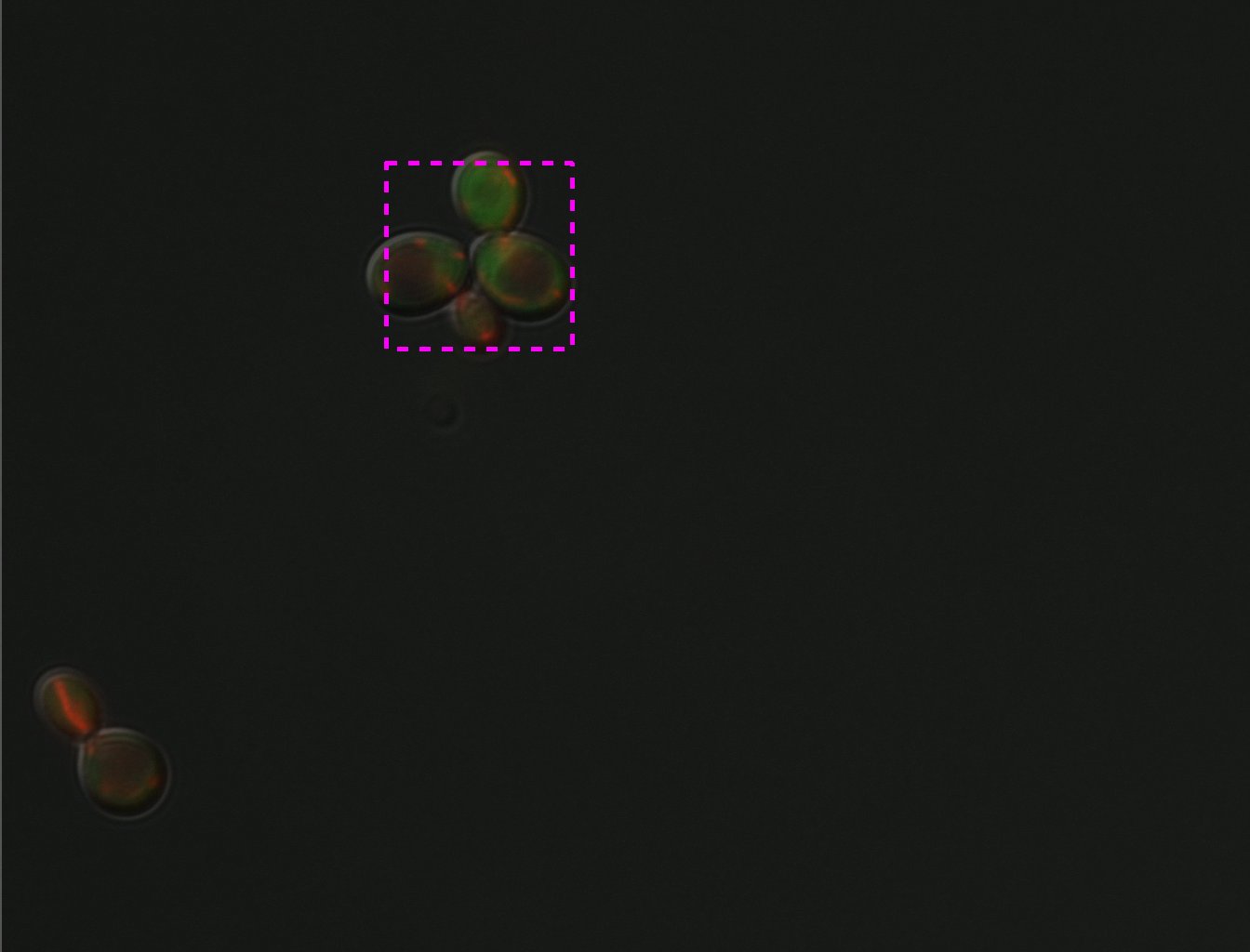

Supplement: Supplementary file 5 — Source data Fig. 4 [file 44319_2024_126_MOESM5_ESM.zip › Figure 4/4A/4A_Image_mNG-Oxr1_overlay.jpg]

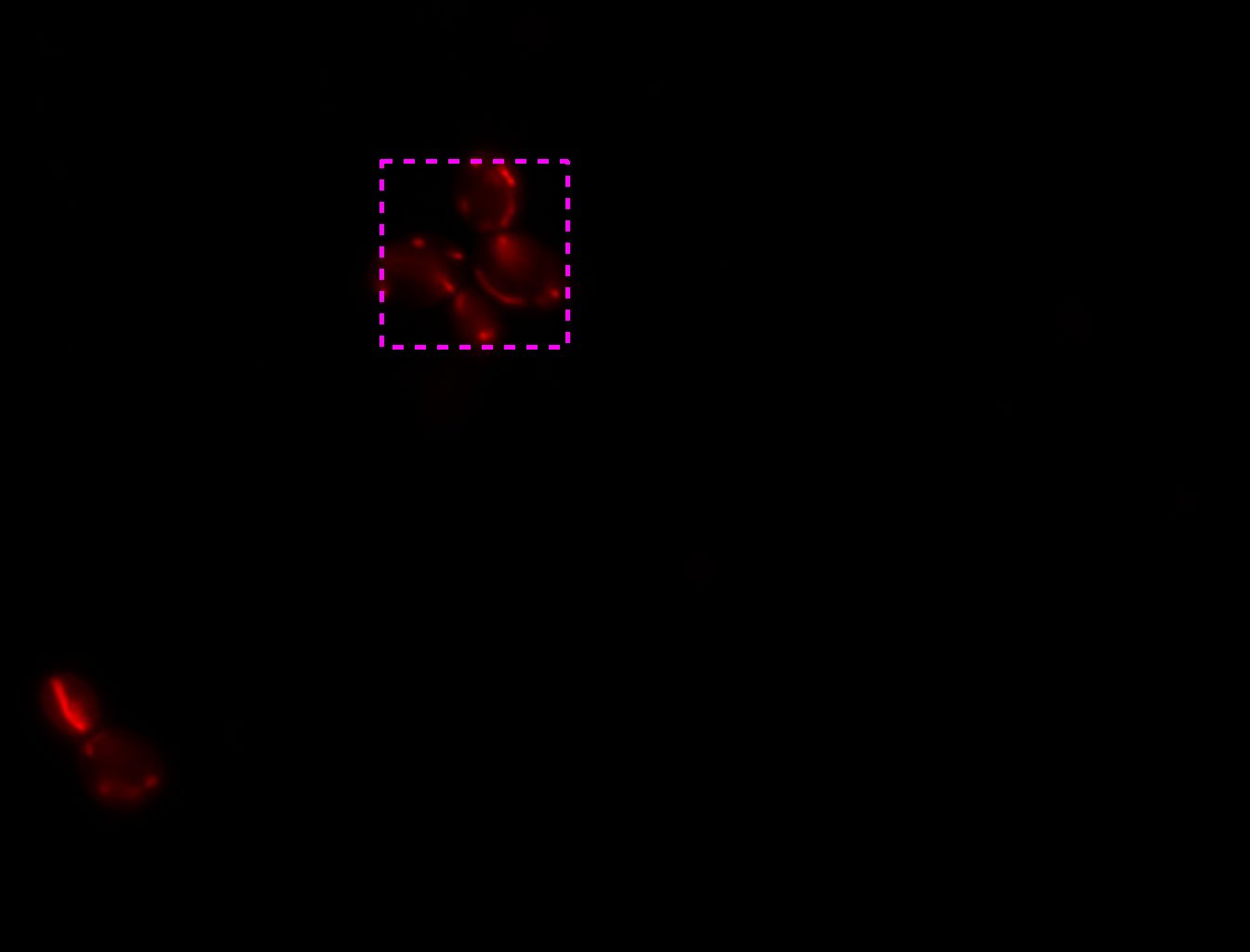

Supplement: Supplementary file 5 — Source data Fig. 4 [file 44319_2024_126_MOESM5_ESM.zip › Figure 4/4A/4A_Image_mNG-Oxr1_Red.jpg]

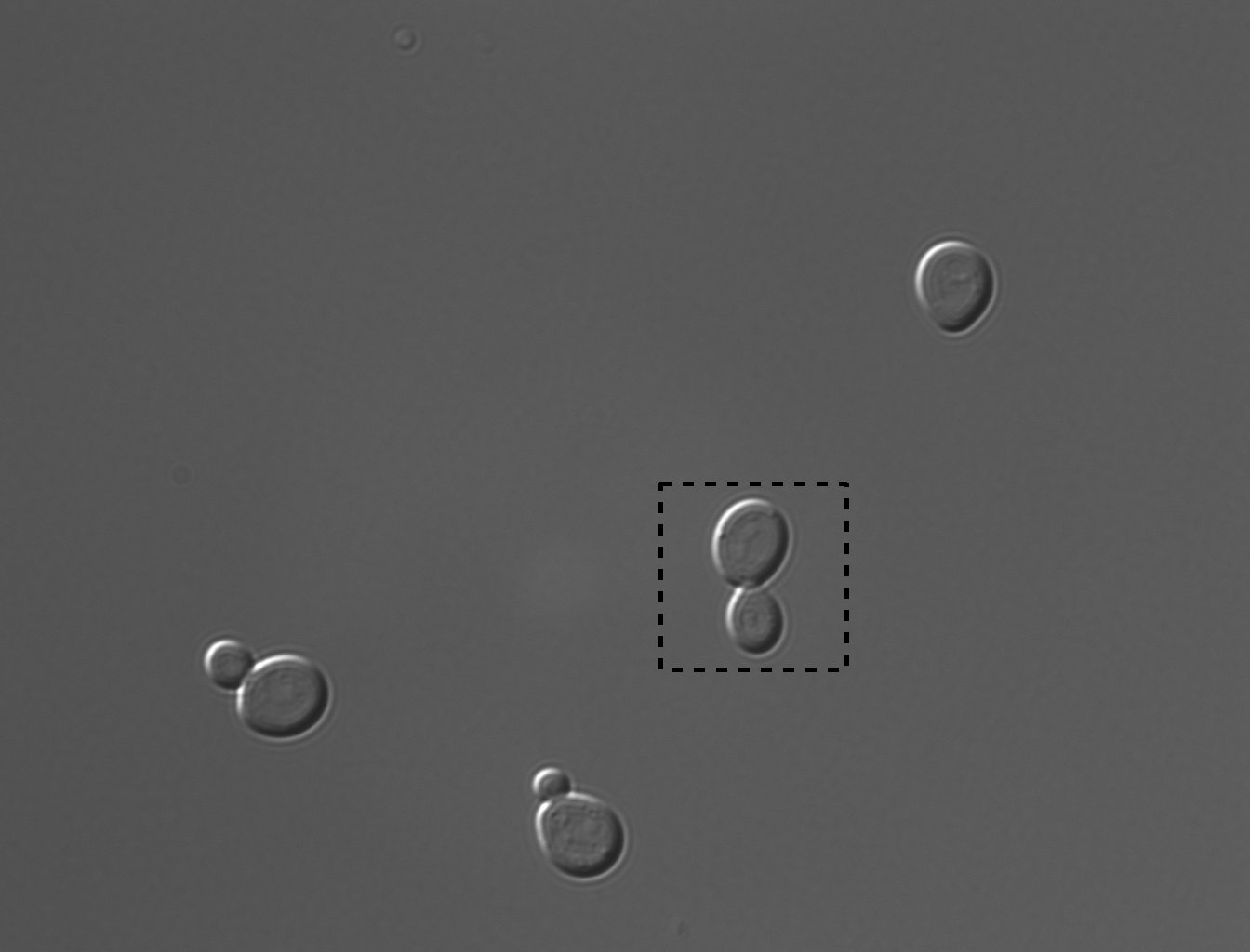

Supplement: Supplementary file 5 — Source data Fig. 4 [file 44319_2024_126_MOESM5_ESM.zip › Figure 4/4A/4A_Image_Oxr1-mNG_DIC.jpg]

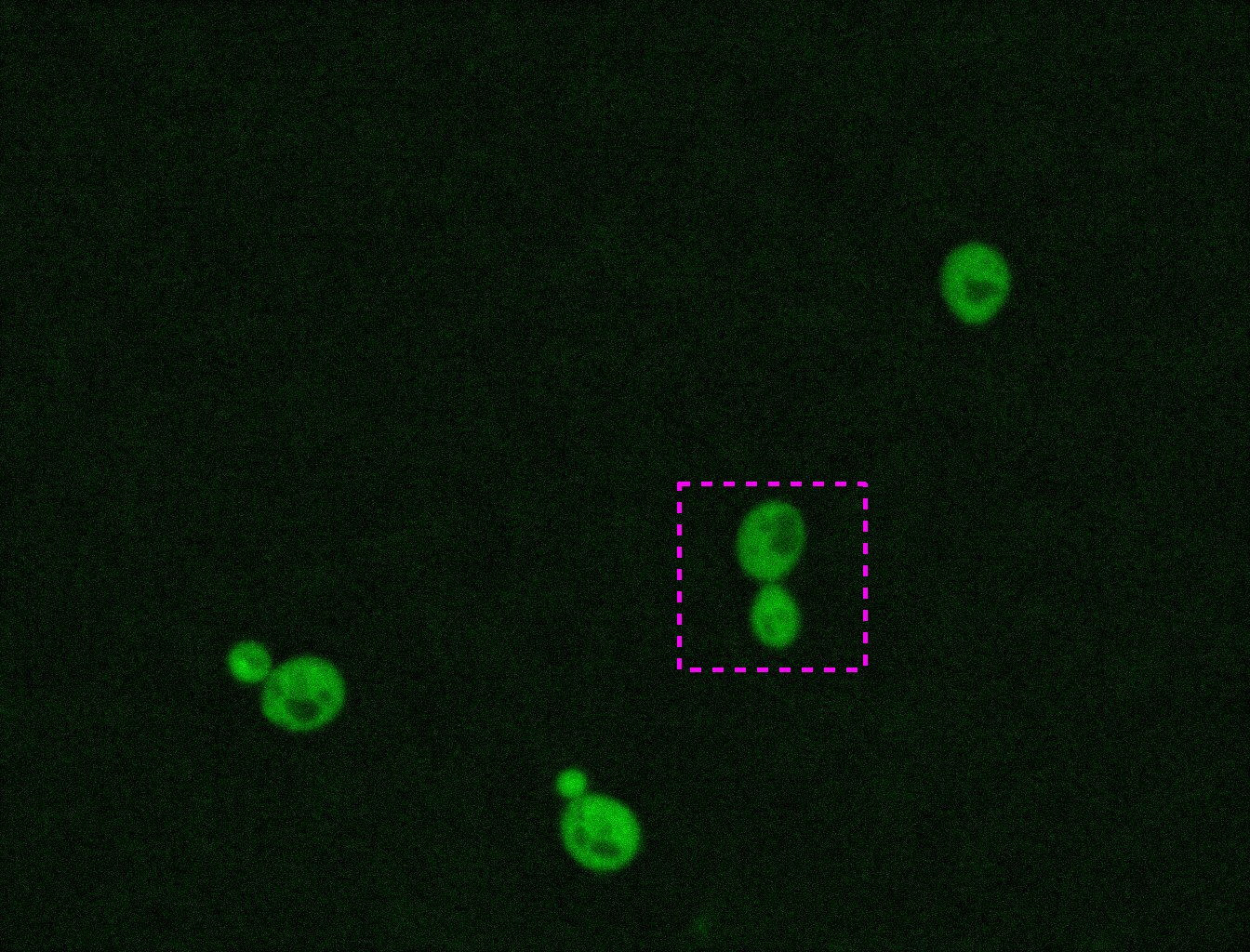

Supplement: Supplementary file 5 — Source data Fig. 4 [file 44319_2024_126_MOESM5_ESM.zip › Figure 4/4A/4A_Image_Oxr1-mNG_Green.jpg]

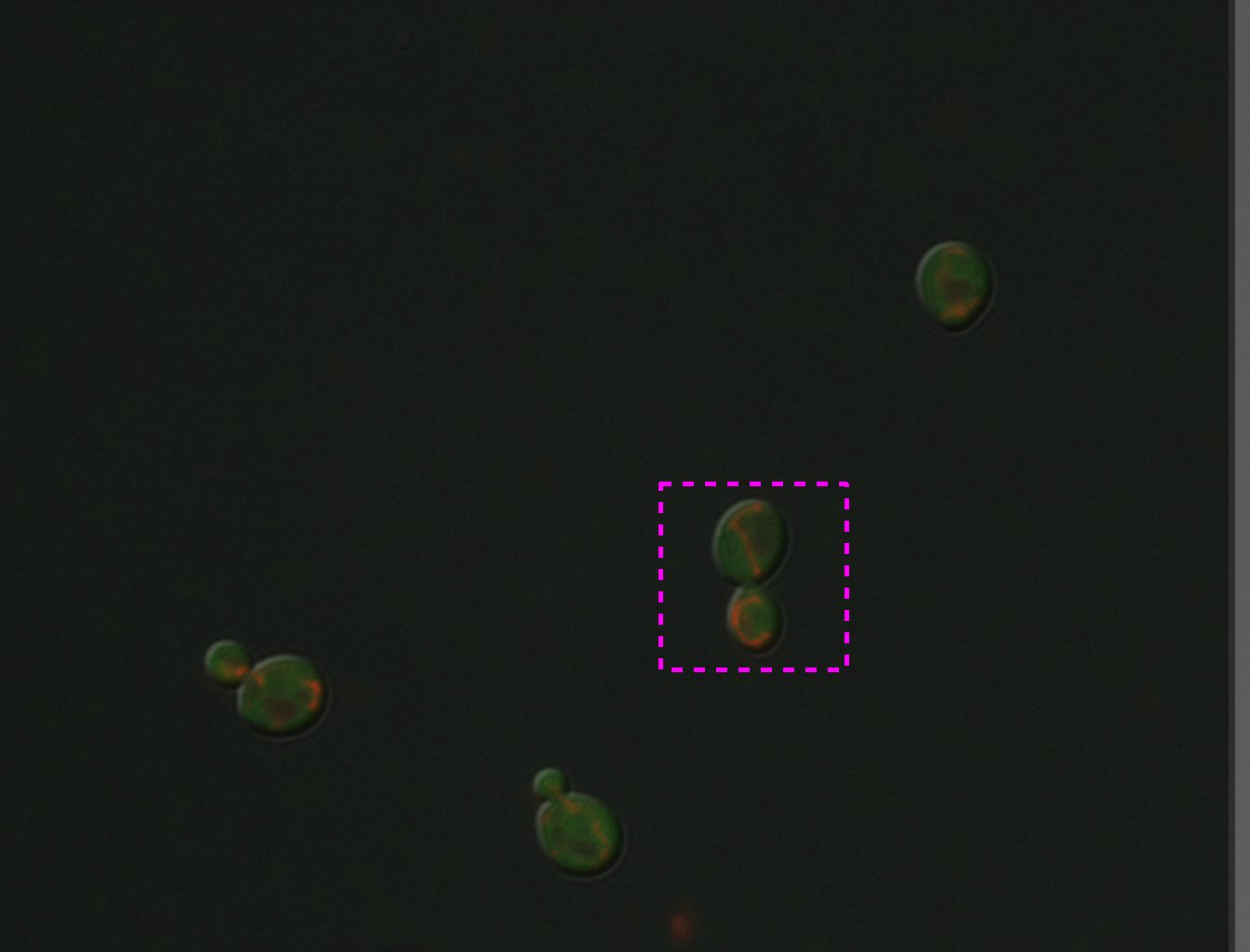

Supplement: Supplementary file 5 — Source data Fig. 4 [file 44319_2024_126_MOESM5_ESM.zip › Figure 4/4A/4A_Image_Oxr1-mNG_overlay.jpg]

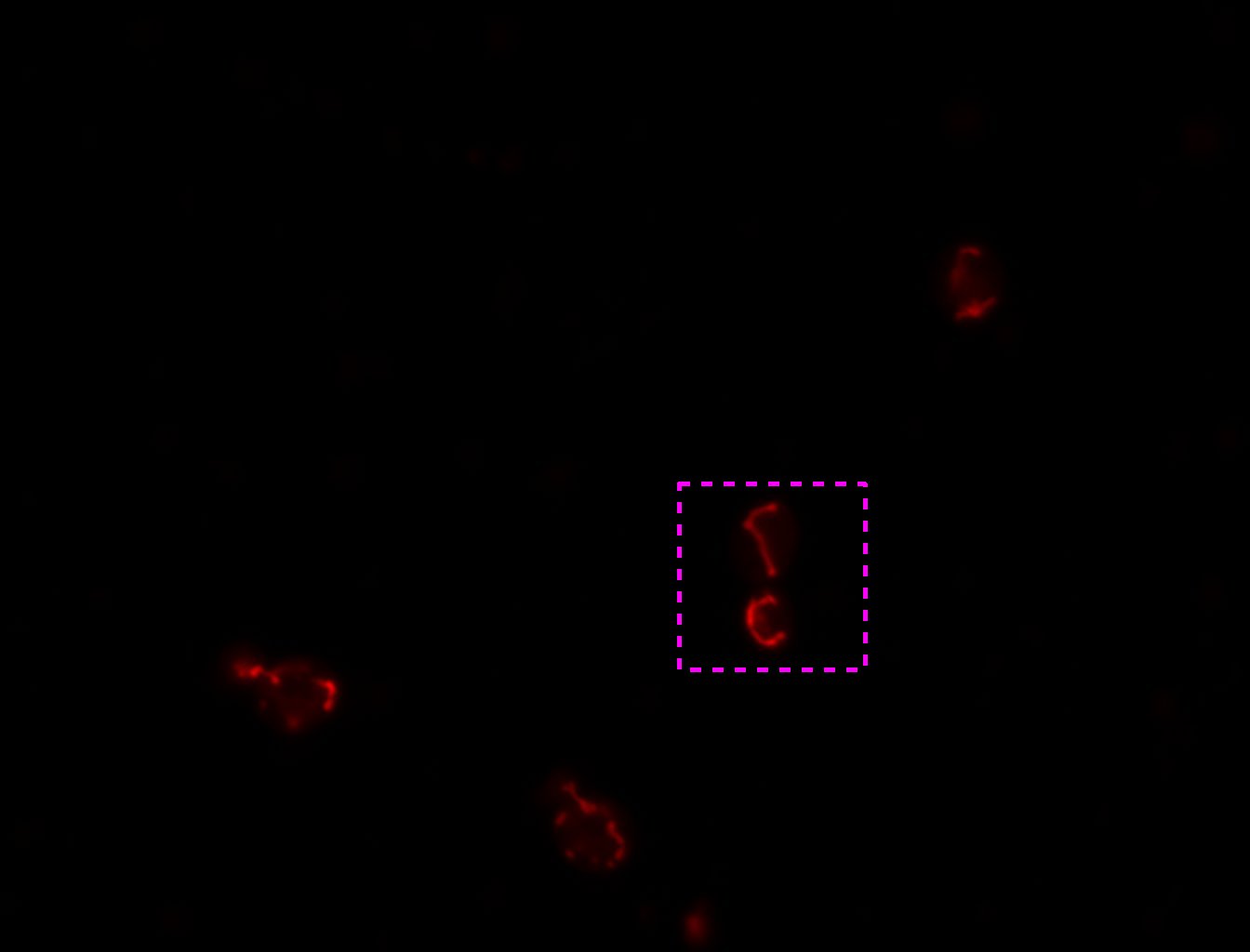

Supplement: Supplementary file 5 — Source data Fig. 4 [file 44319_2024_126_MOESM5_ESM.zip › Figure 4/4A/4A_Image_Oxr1-mNG_Red.jpg]

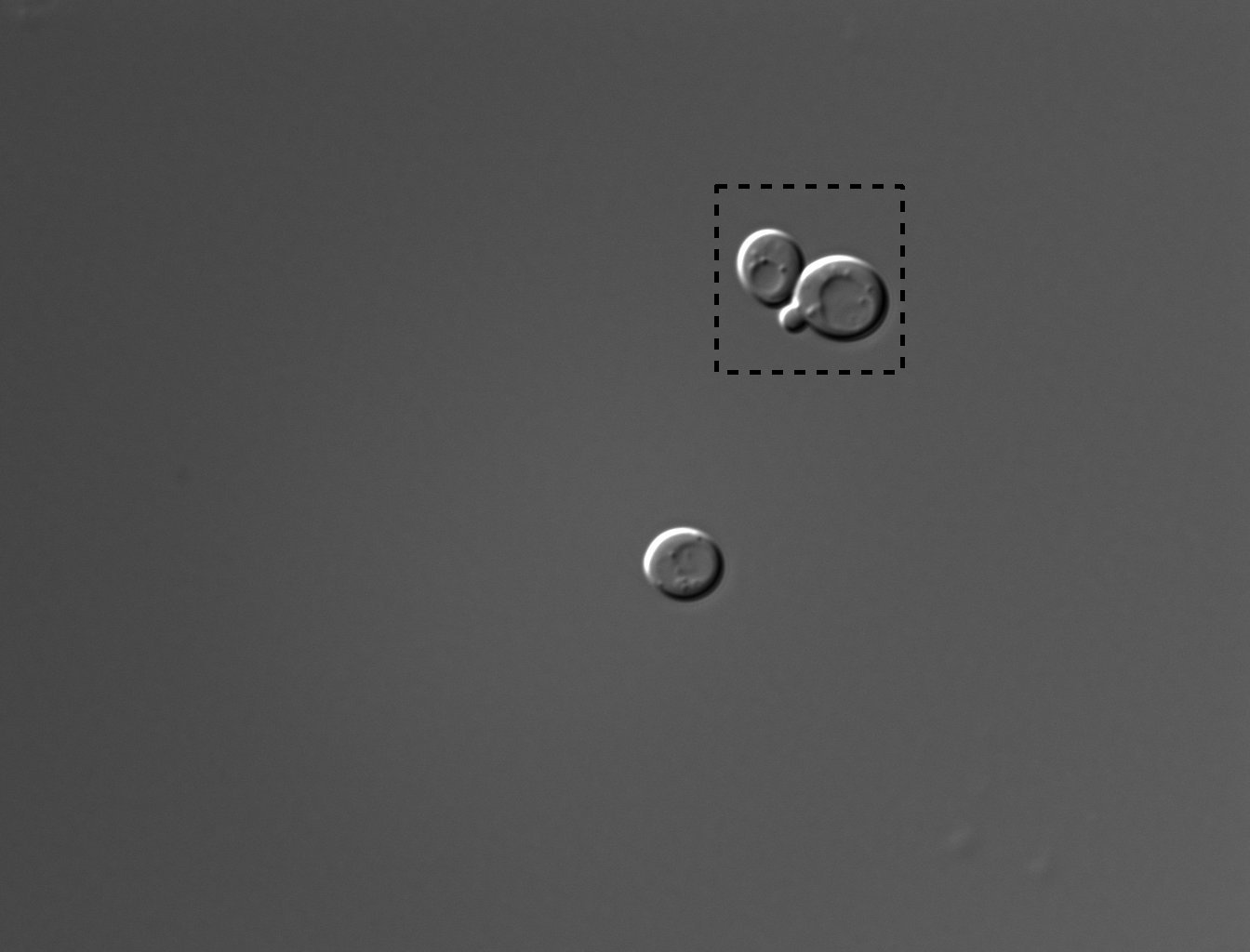

Supplement: Supplementary file 5 — Source data Fig. 4 [file 44319_2024_126_MOESM5_ESM.zip › Figure 4/4B/4B_Image_Oxr1-mNG_glucose deprived_DIC.jpg]

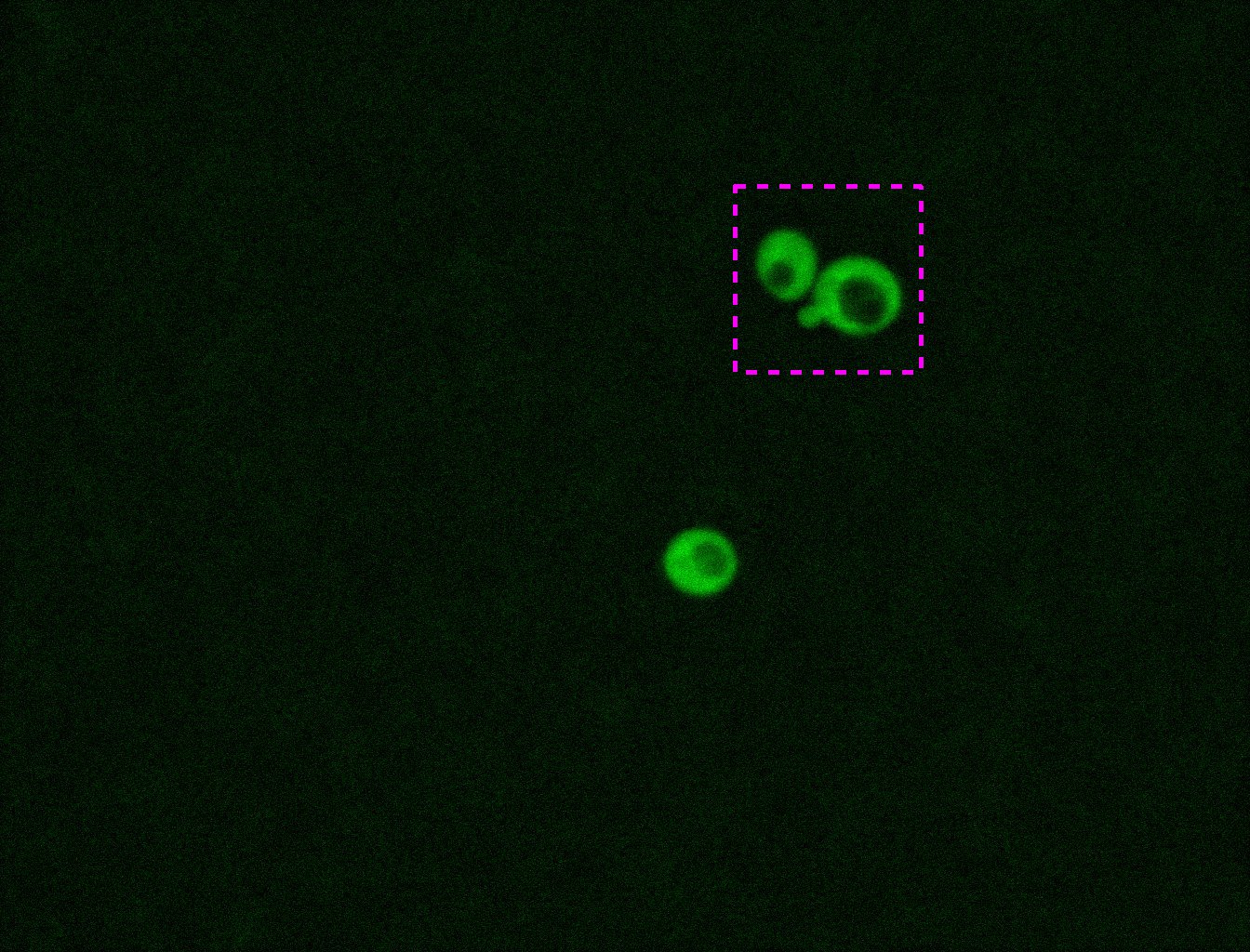

Supplement: Supplementary file 5 — Source data Fig. 4 [file 44319_2024_126_MOESM5_ESM.zip › Figure 4/4B/4B_Image_Oxr1-mNG_glucose deprived_Green.jpg]

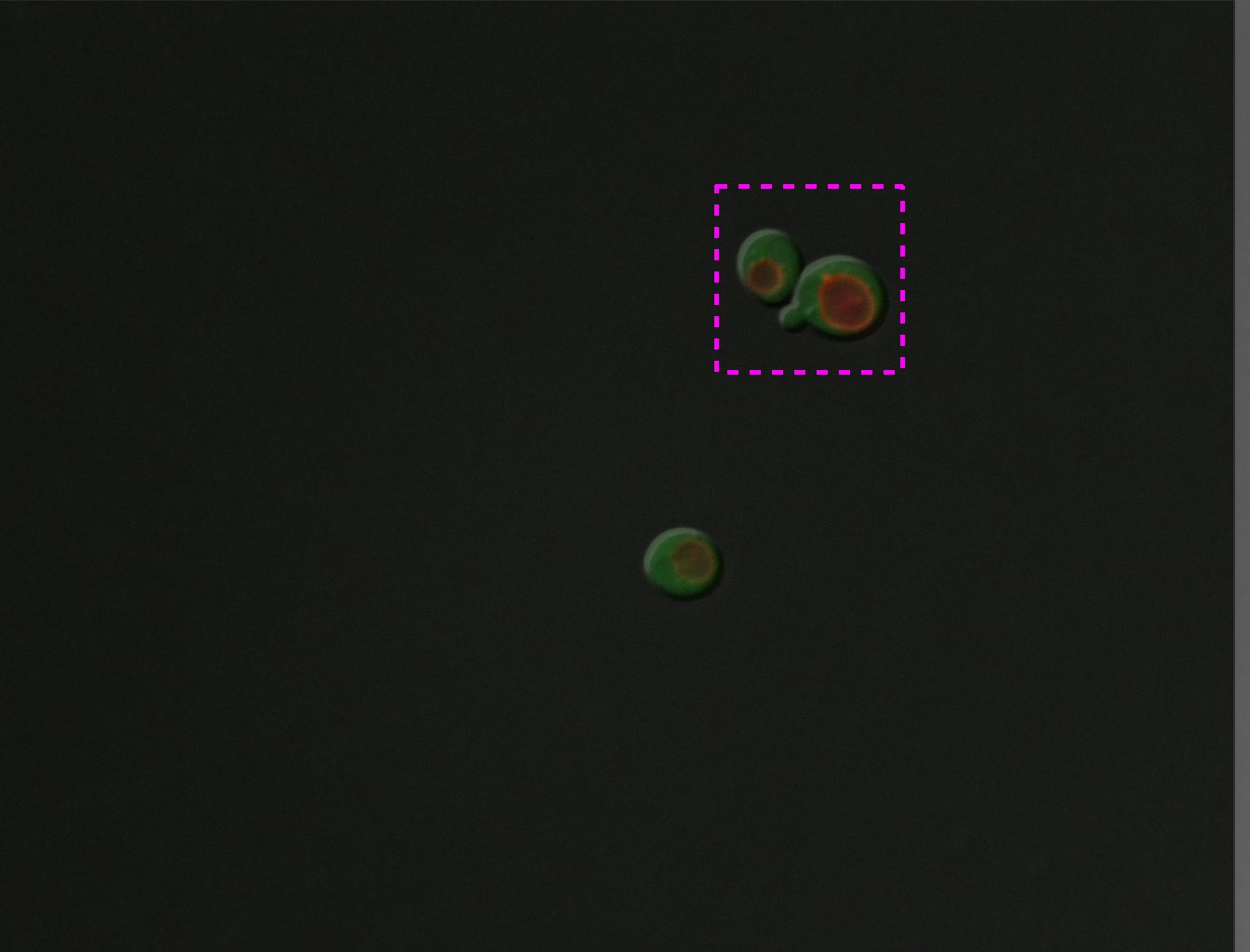

Supplement: Supplementary file 5 — Source data Fig. 4 [file 44319_2024_126_MOESM5_ESM.zip › Figure 4/4B/4B_Image_Oxr1-mNG_glucose deprived_overlay.jpg]

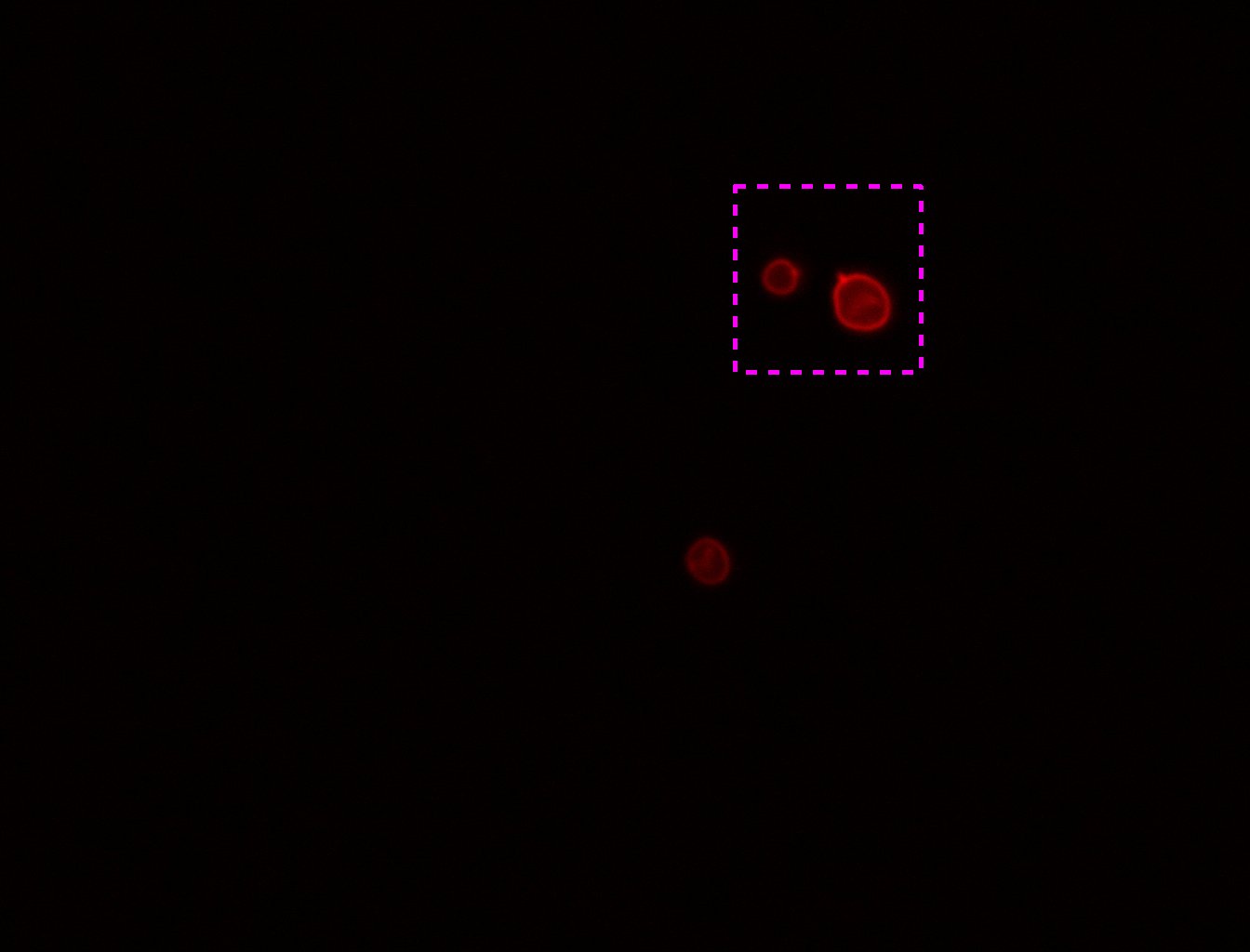

Supplement: Supplementary file 5 — Source data Fig. 4 [file 44319_2024_126_MOESM5_ESM.zip › Figure 4/4B/4B_Image_Oxr1-mNG_glucose deprived_Red.jpg]

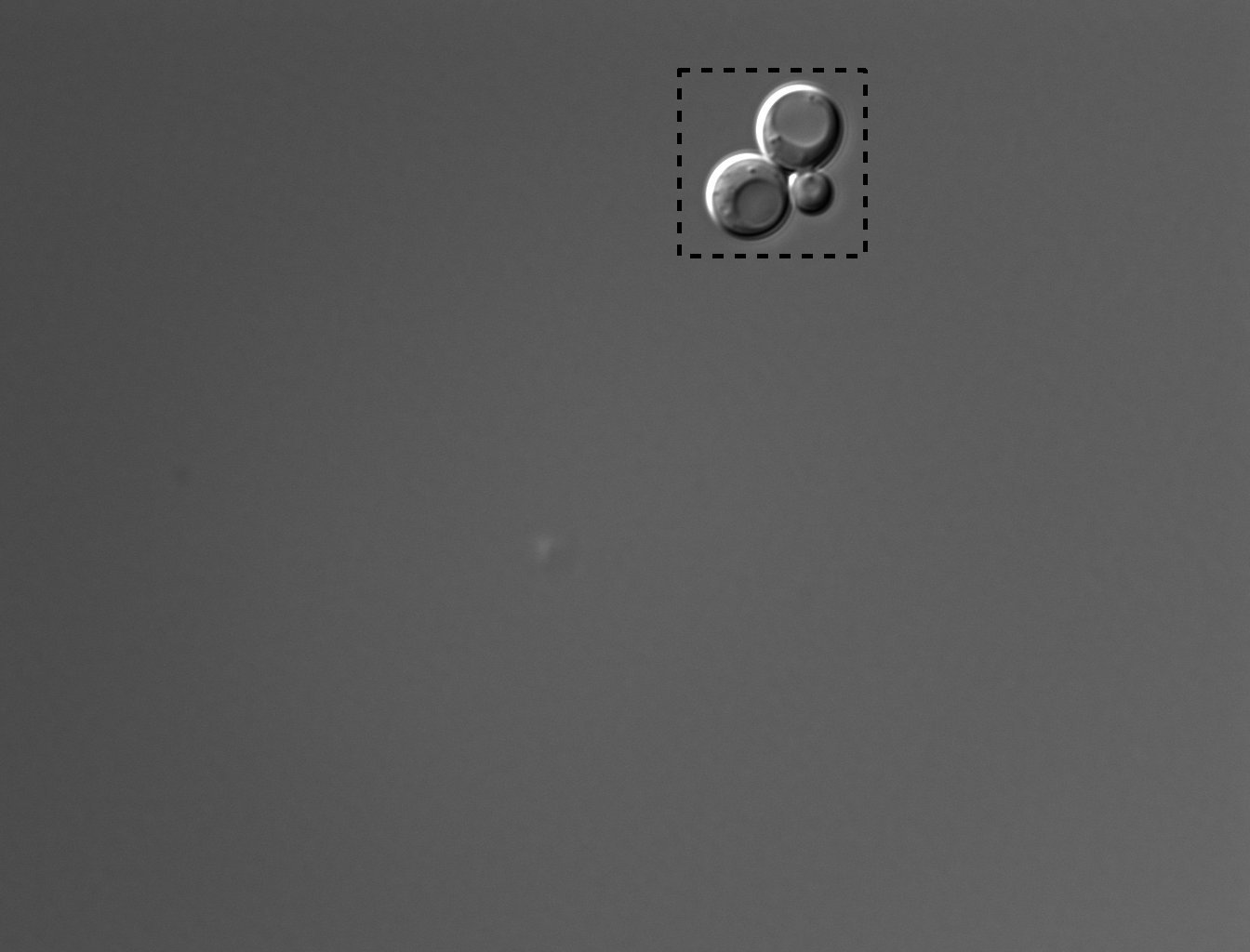

Supplement: Supplementary file 5 — Source data Fig. 4 [file 44319_2024_126_MOESM5_ESM.zip › Figure 4/4B/4B_Image_Oxr1-mNG_glucose readd_DIC.jpg]

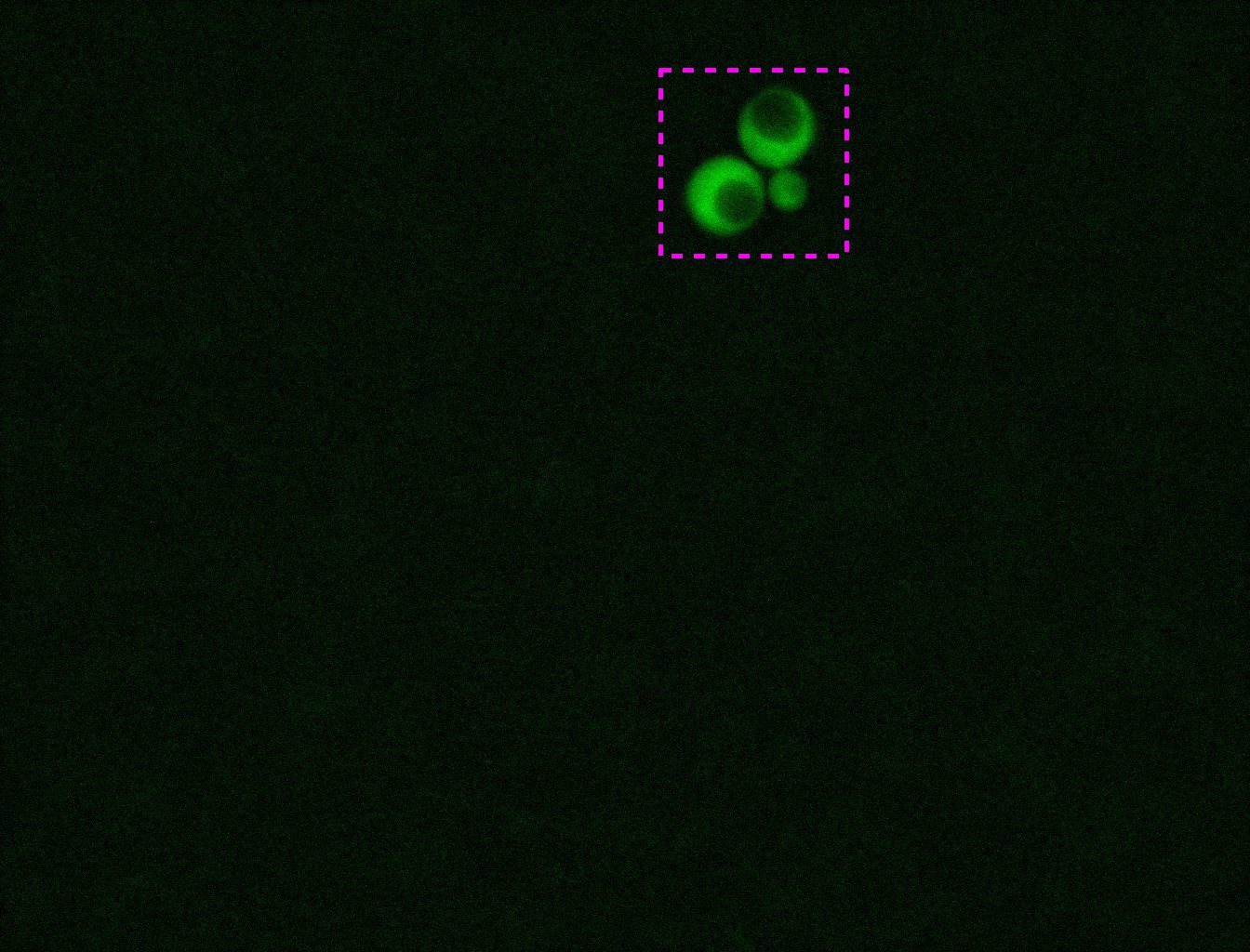

Supplement: Supplementary file 5 — Source data Fig. 4 [file 44319_2024_126_MOESM5_ESM.zip › Figure 4/4B/4B_Image_Oxr1-mNG_glucose readd_Green.jpg]

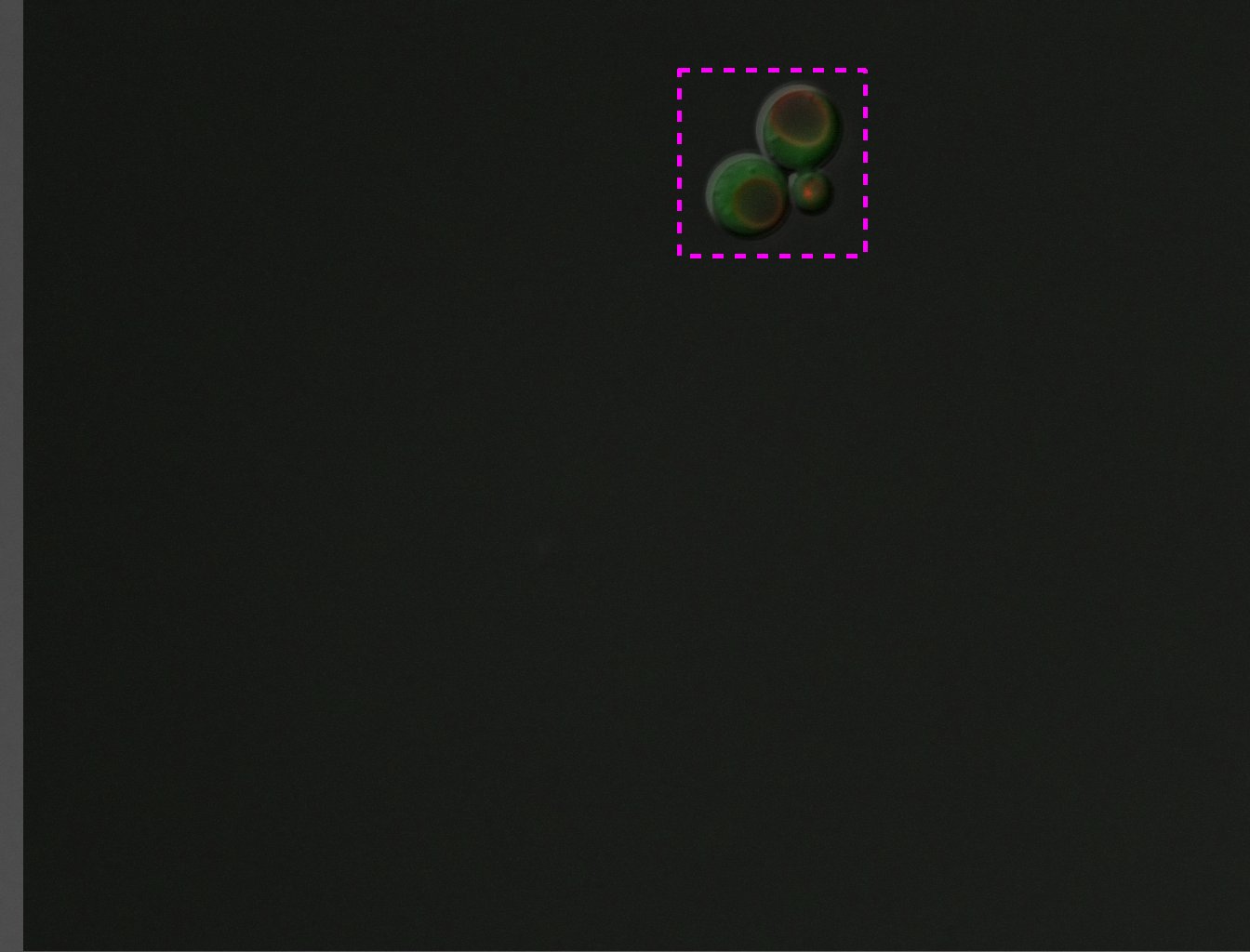

Supplement: Supplementary file 5 — Source data Fig. 4 [file 44319_2024_126_MOESM5_ESM.zip › Figure 4/4B/4B_Image_Oxr1-mNG_glucose readd_overlay.jpg]

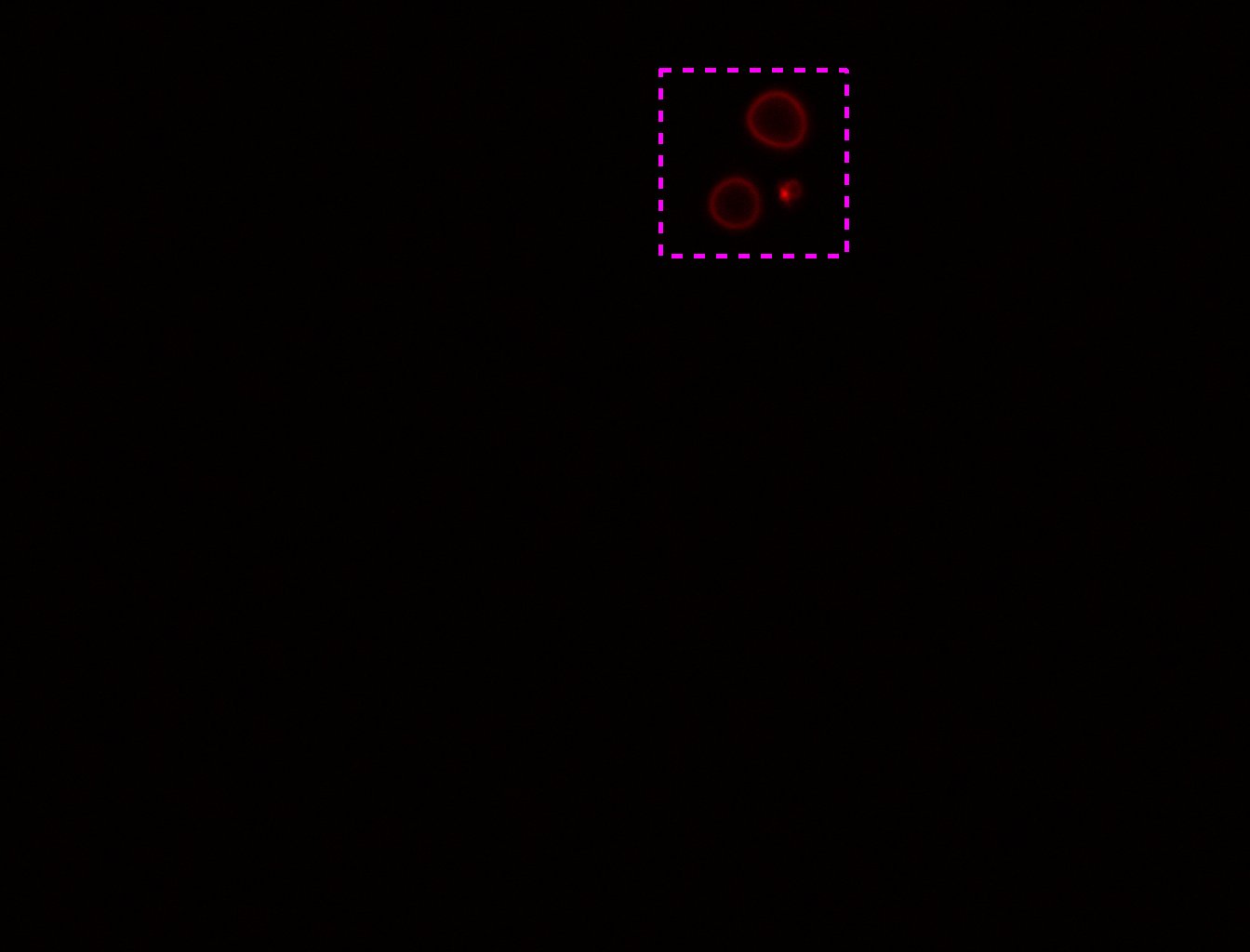

Supplement: Supplementary file 5 — Source data Fig. 4 [file 44319_2024_126_MOESM5_ESM.zip › Figure 4/4B/4B_Image_Oxr1-mNG_glucose readd_Red.jpg]

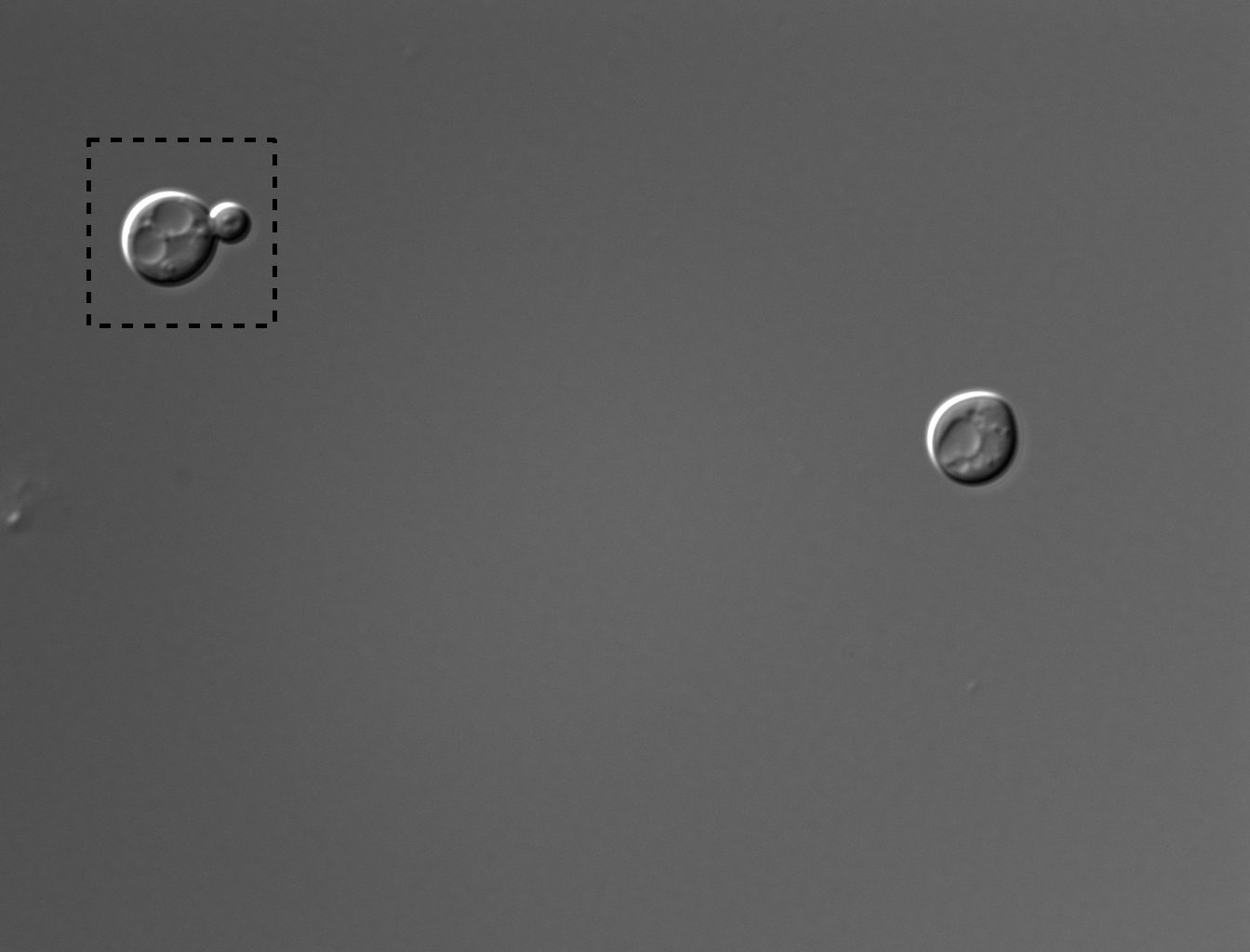

Supplement: Supplementary file 5 — Source data Fig. 4 [file 44319_2024_126_MOESM5_ESM.zip › Figure 4/4B/4B_Image_Oxr1-mNG_glucose_DIC.jpg]

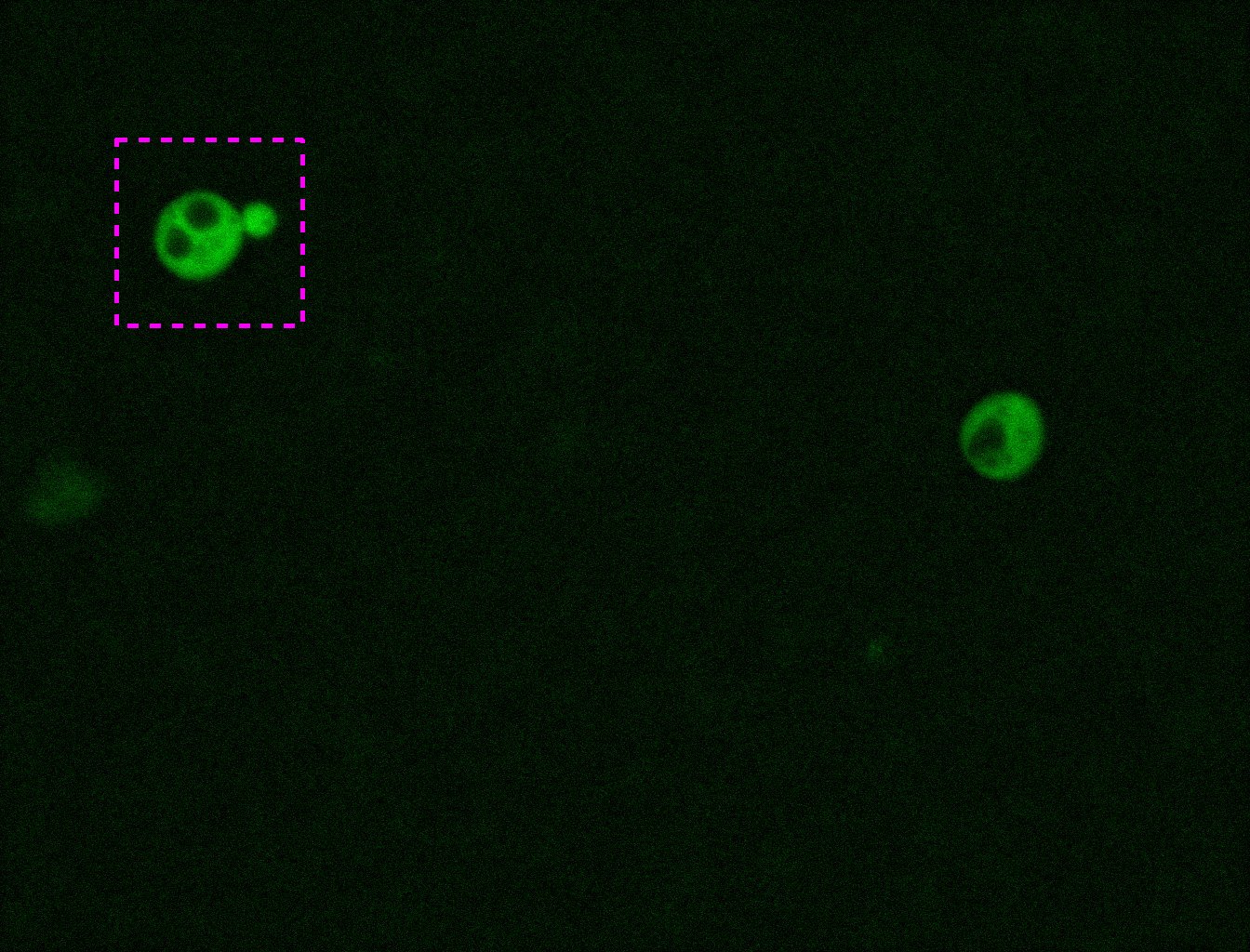

Supplement: Supplementary file 5 — Source data Fig. 4 [file 44319_2024_126_MOESM5_ESM.zip › Figure 4/4B/4B_Image_Oxr1-mNG_glucose_Green.jpg]

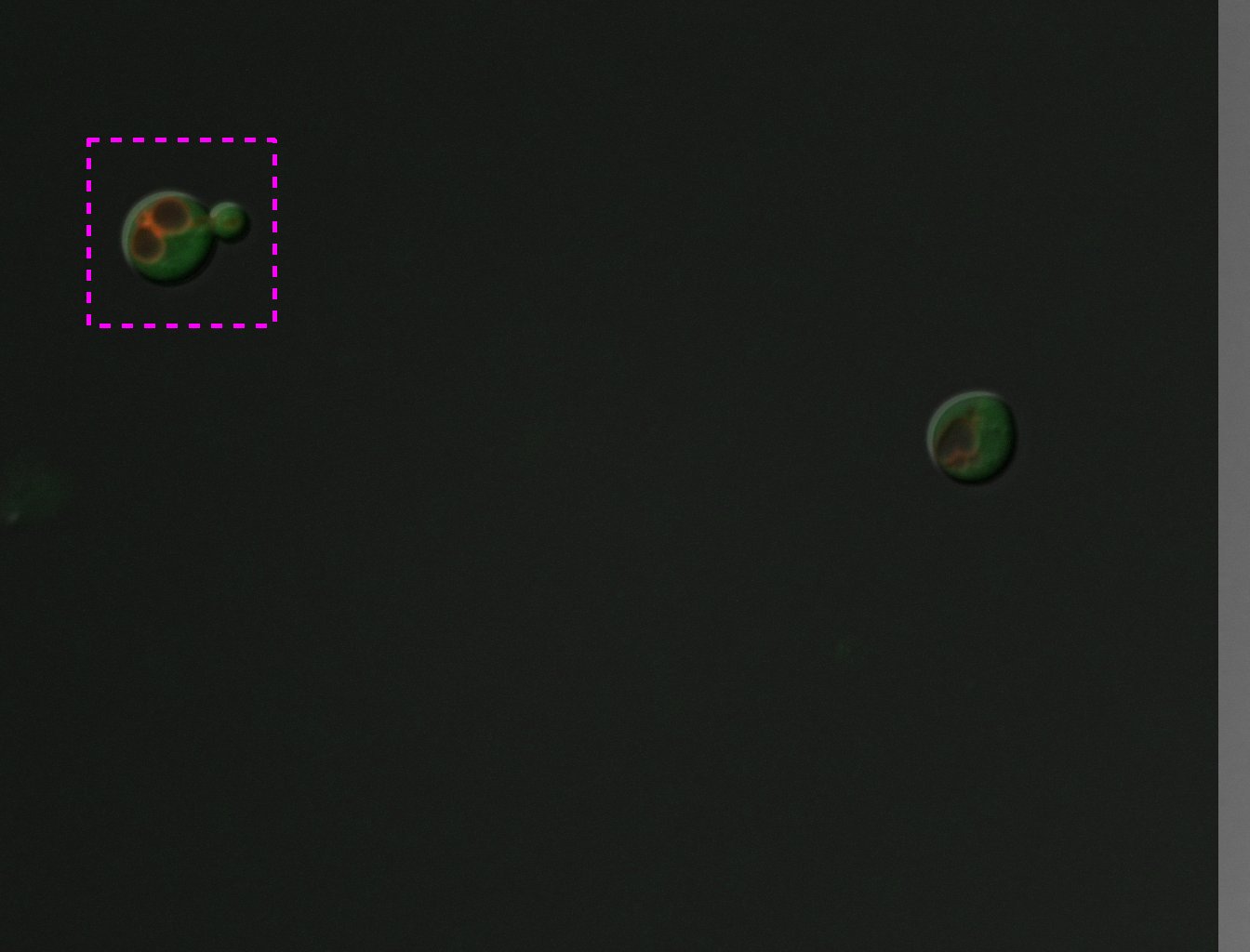

Supplement: Supplementary file 5 — Source data Fig. 4 [file 44319_2024_126_MOESM5_ESM.zip › Figure 4/4B/4B_Image_Oxr1-mNG_glucose_overlay.jpg]

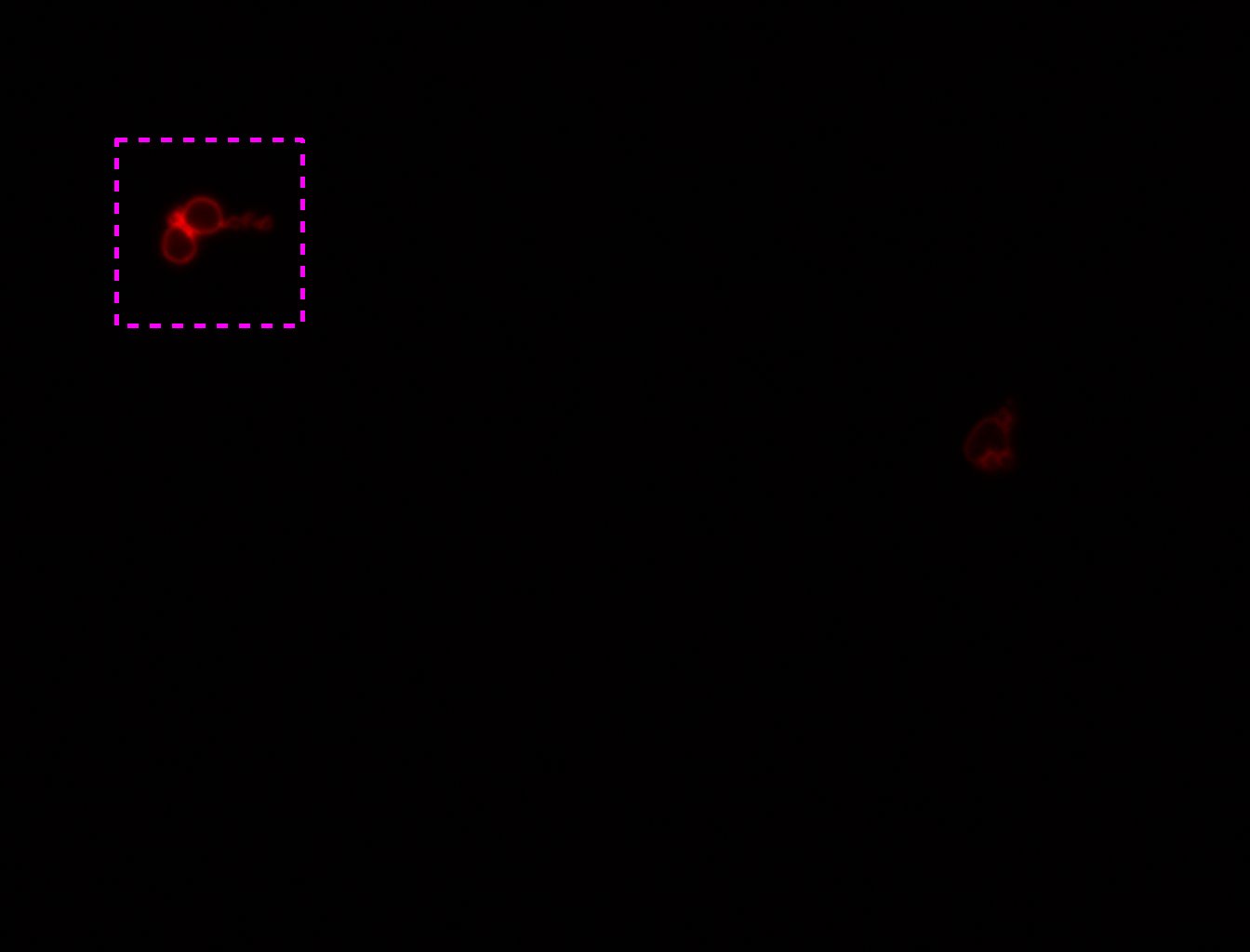

Supplement: Supplementary file 5 — Source data Fig. 4 [file 44319_2024_126_MOESM5_ESM.zip › Figure 4/4B/4B_Image_Oxr1-mNG_glucose_Red.jpg]

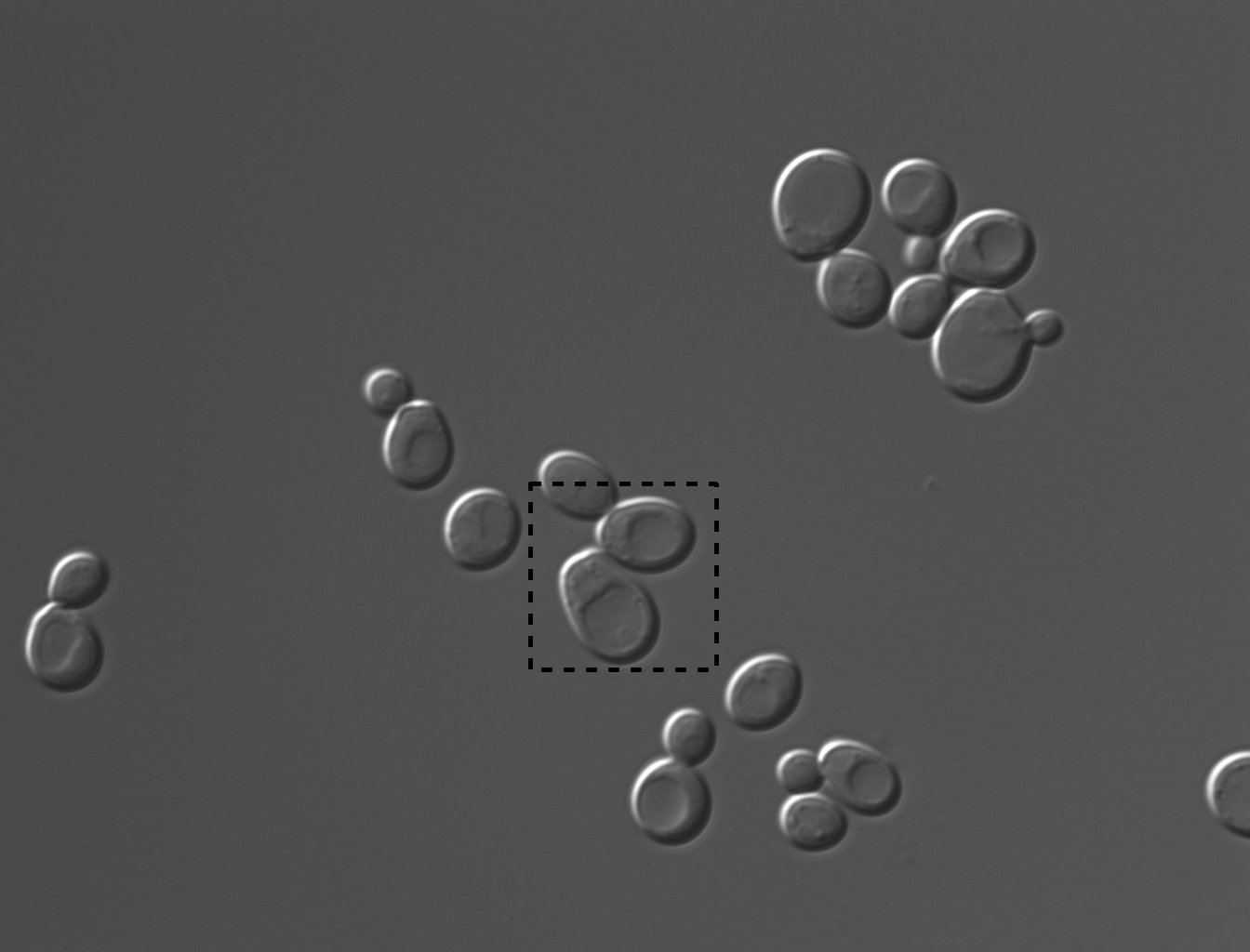

Supplement: Supplementary file 5 — Source data Fig. 4 [file 44319_2024_126_MOESM5_ESM.zip › Figure 4/4C/4C_Image_mNG-Oxr1_glucose deprived_DIC.jpg]

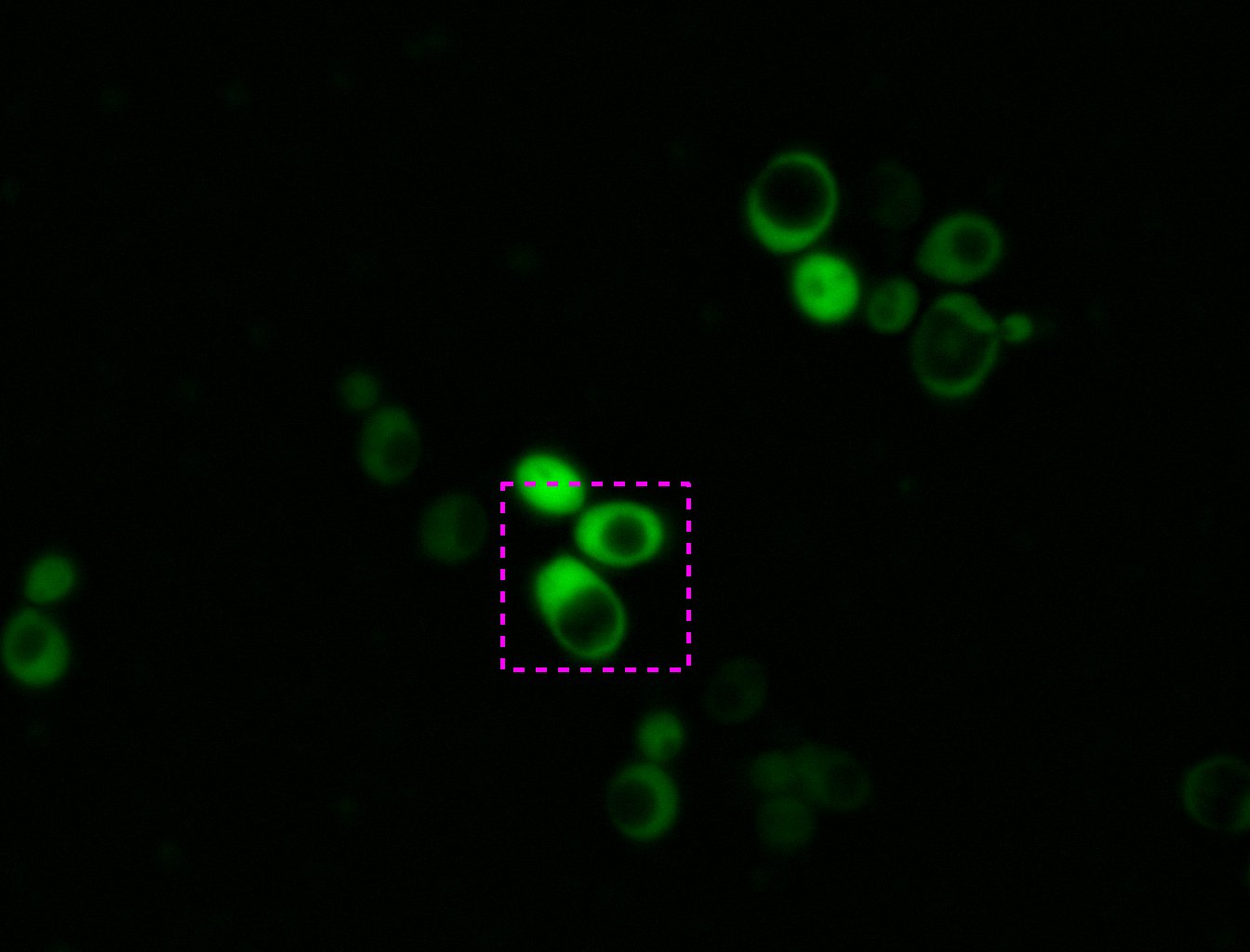

Supplement: Supplementary file 5 — Source data Fig. 4 [file 44319_2024_126_MOESM5_ESM.zip › Figure 4/4C/4C_Image_mNG-Oxr1_glucose deprived_Green.jpg]

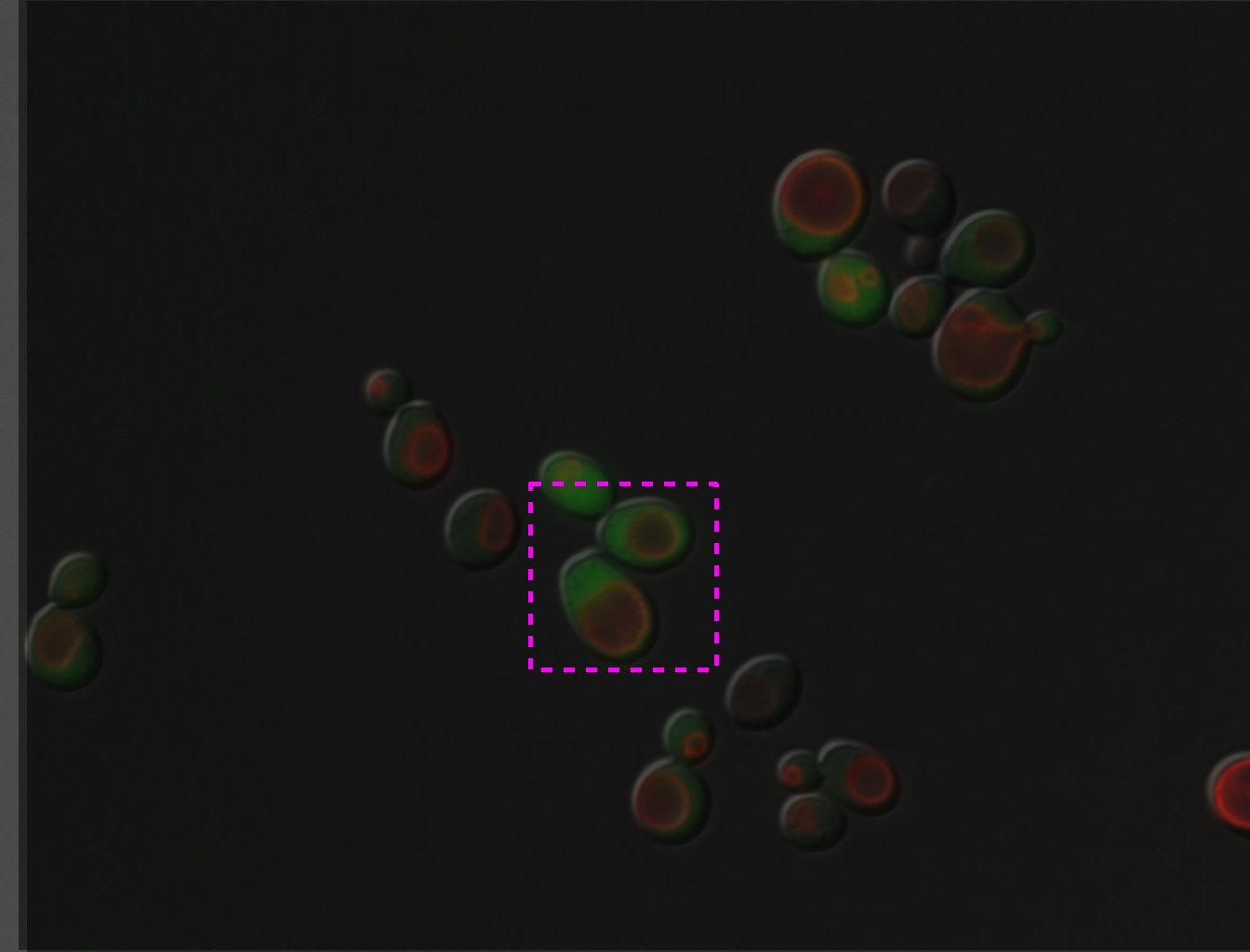

Supplement: Supplementary file 5 — Source data Fig. 4 [file 44319_2024_126_MOESM5_ESM.zip › Figure 4/4C/4C_Image_mNG-Oxr1_glucose deprived_overlay.jpg]

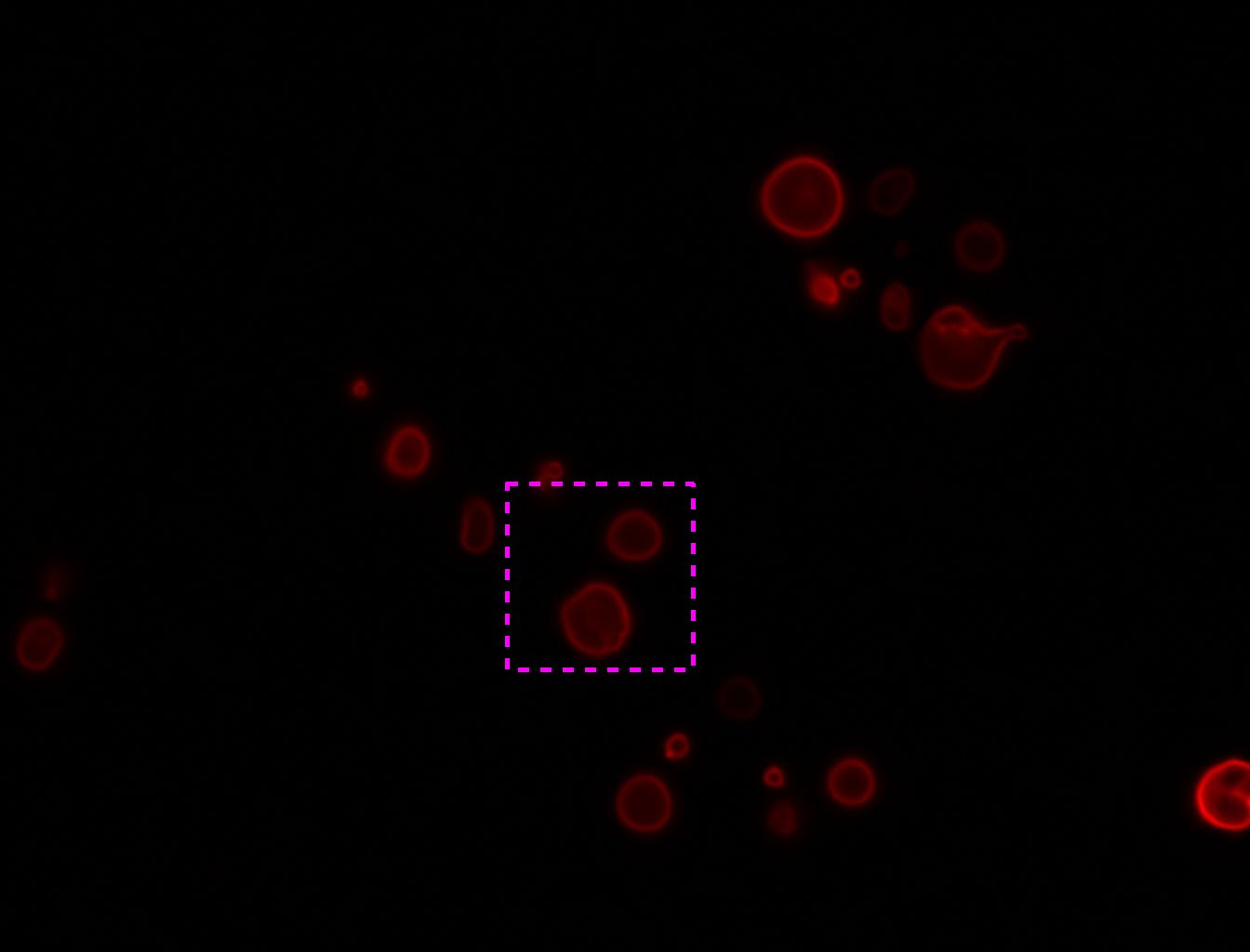

Supplement: Supplementary file 5 — Source data Fig. 4 [file 44319_2024_126_MOESM5_ESM.zip › Figure 4/4C/4C_Image_mNG-Oxr1_glucose deprived_Red.jpg]

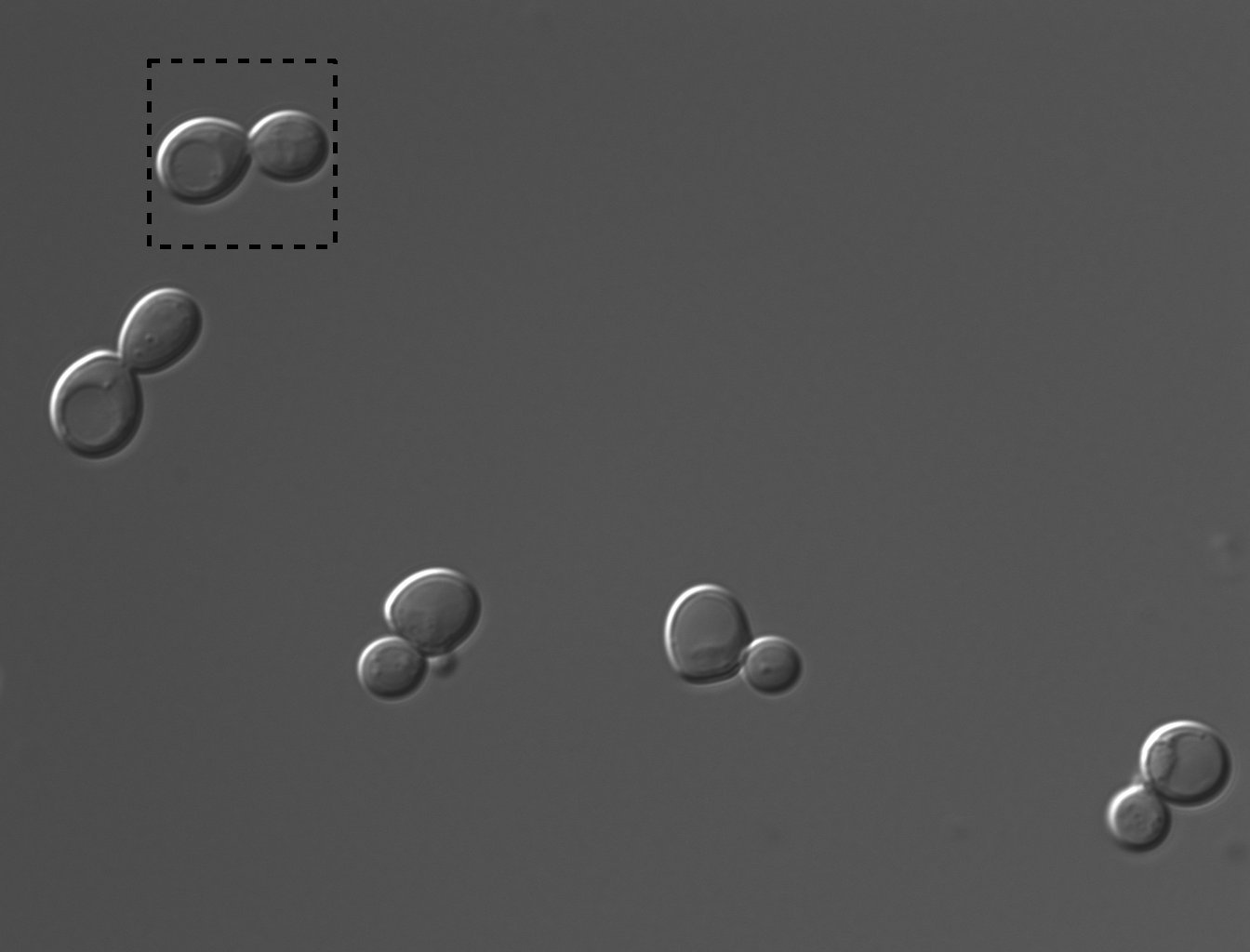

Supplement: Supplementary file 5 — Source data Fig. 4 [file 44319_2024_126_MOESM5_ESM.zip › Figure 4/4C/4C_Image_mNG-Oxr1_glucose readd_DIC.jpg]

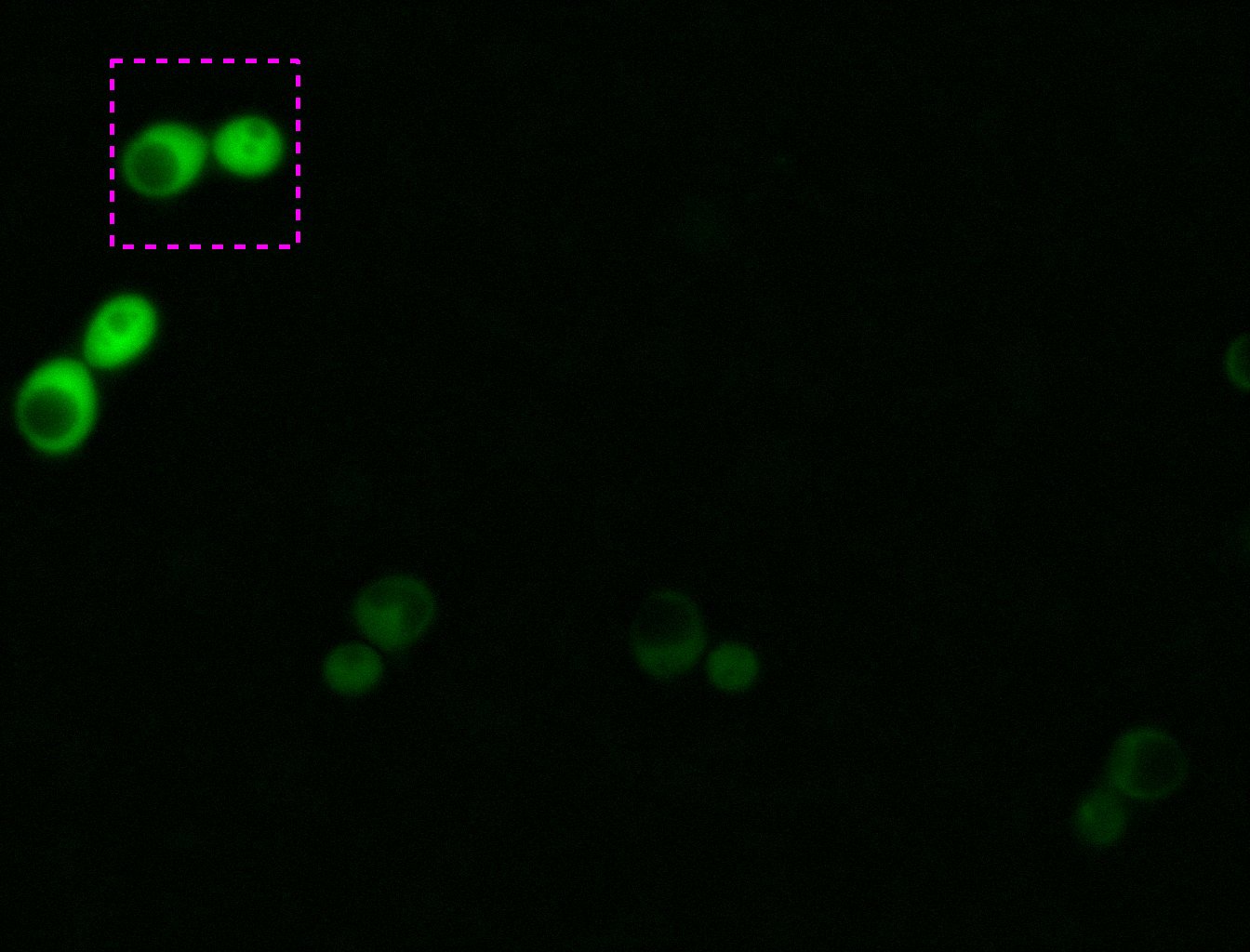

Supplement: Supplementary file 5 — Source data Fig. 4 [file 44319_2024_126_MOESM5_ESM.zip › Figure 4/4C/4C_Image_mNG-Oxr1_glucose readd_Green.jpg]

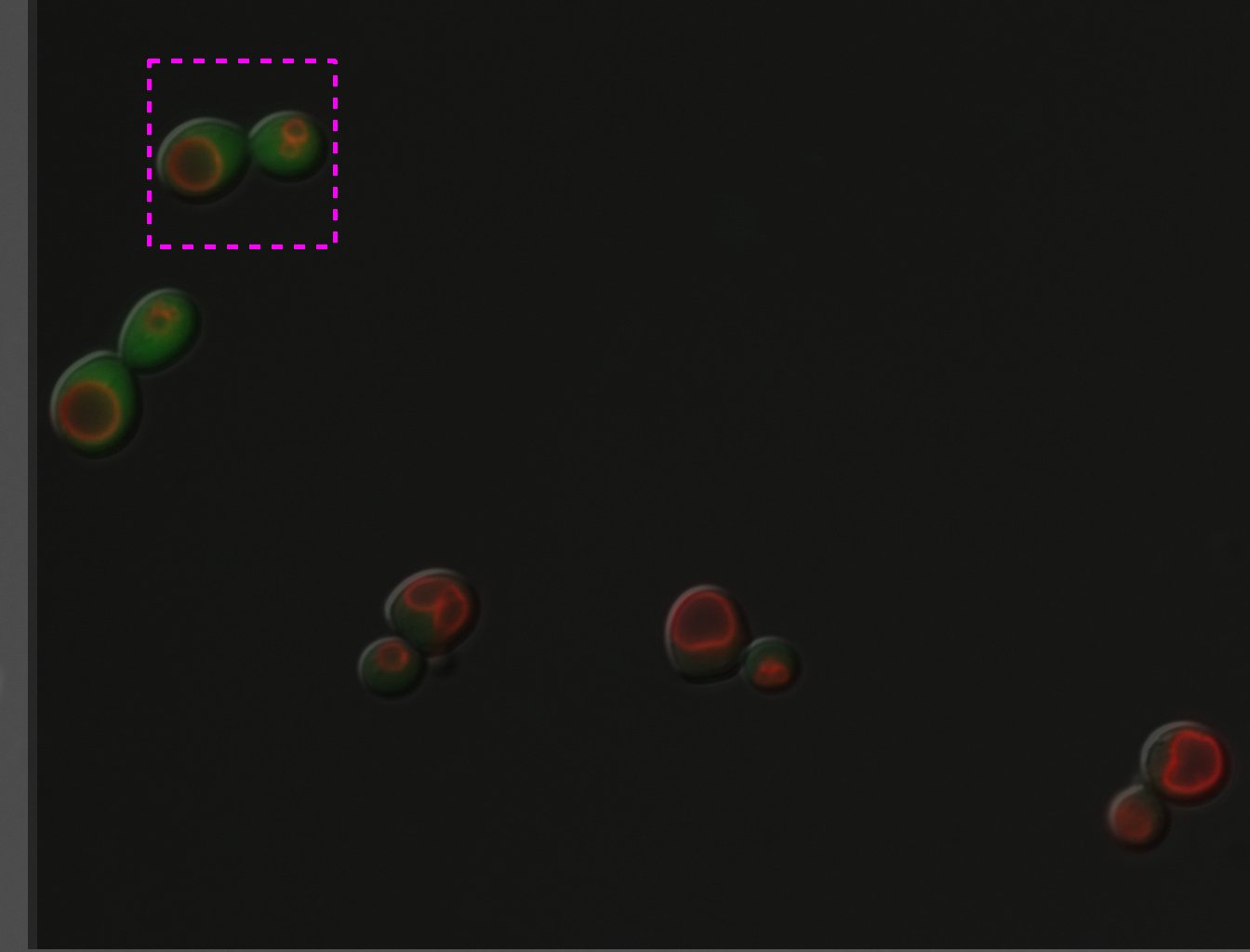

Supplement: Supplementary file 5 — Source data Fig. 4 [file 44319_2024_126_MOESM5_ESM.zip › Figure 4/4C/4C_Image_mNG-Oxr1_glucose readd_overlay.jpg]

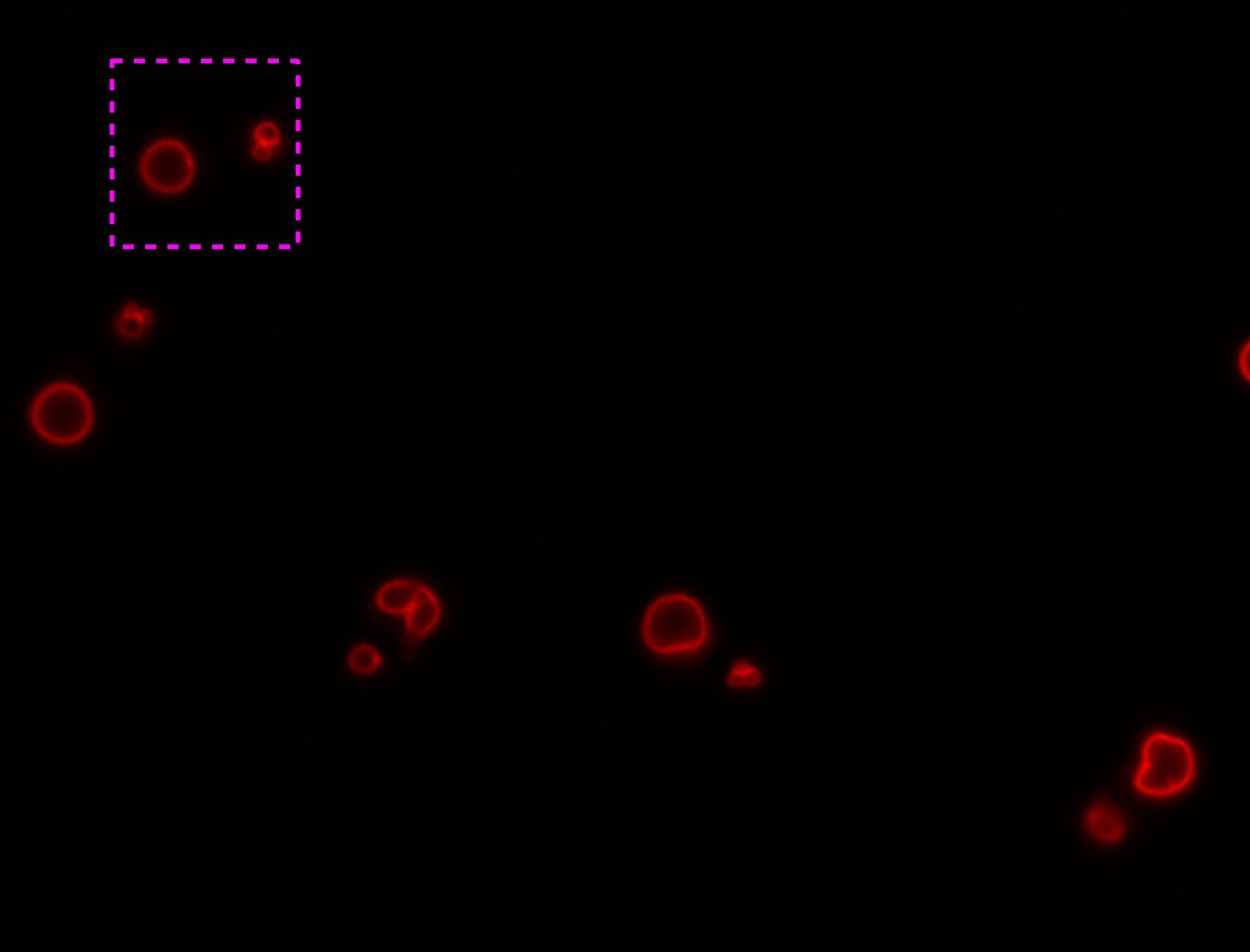

Supplement: Supplementary file 5 — Source data Fig. 4 [file 44319_2024_126_MOESM5_ESM.zip › Figure 4/4C/4C_Image_mNG-Oxr1_glucose readd_Red.jpg]

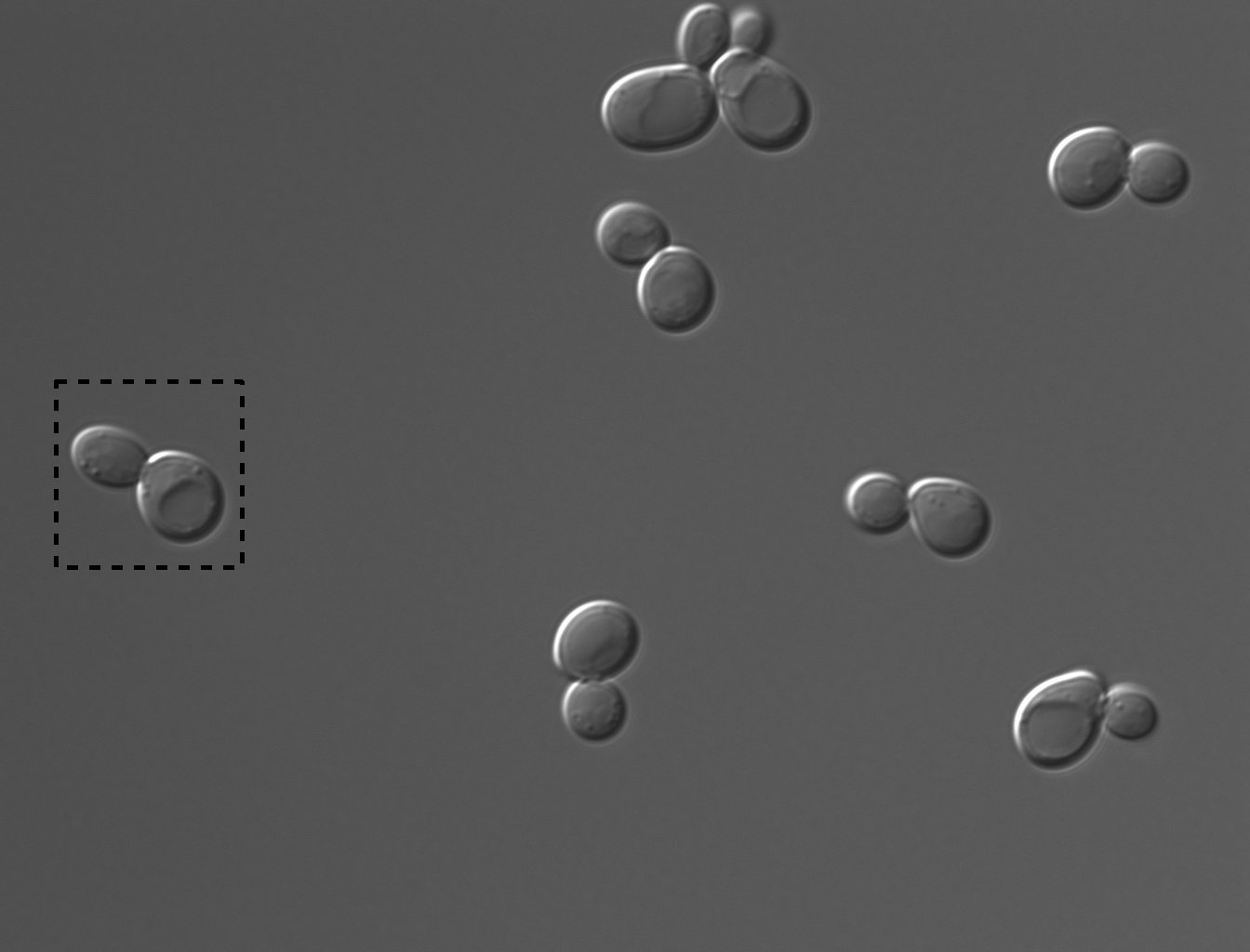

Supplement: Supplementary file 5 — Source data Fig. 4 [file 44319_2024_126_MOESM5_ESM.zip › Figure 4/4C/4C_Image_mNG-Oxr1_glucose_DIC.jpg]

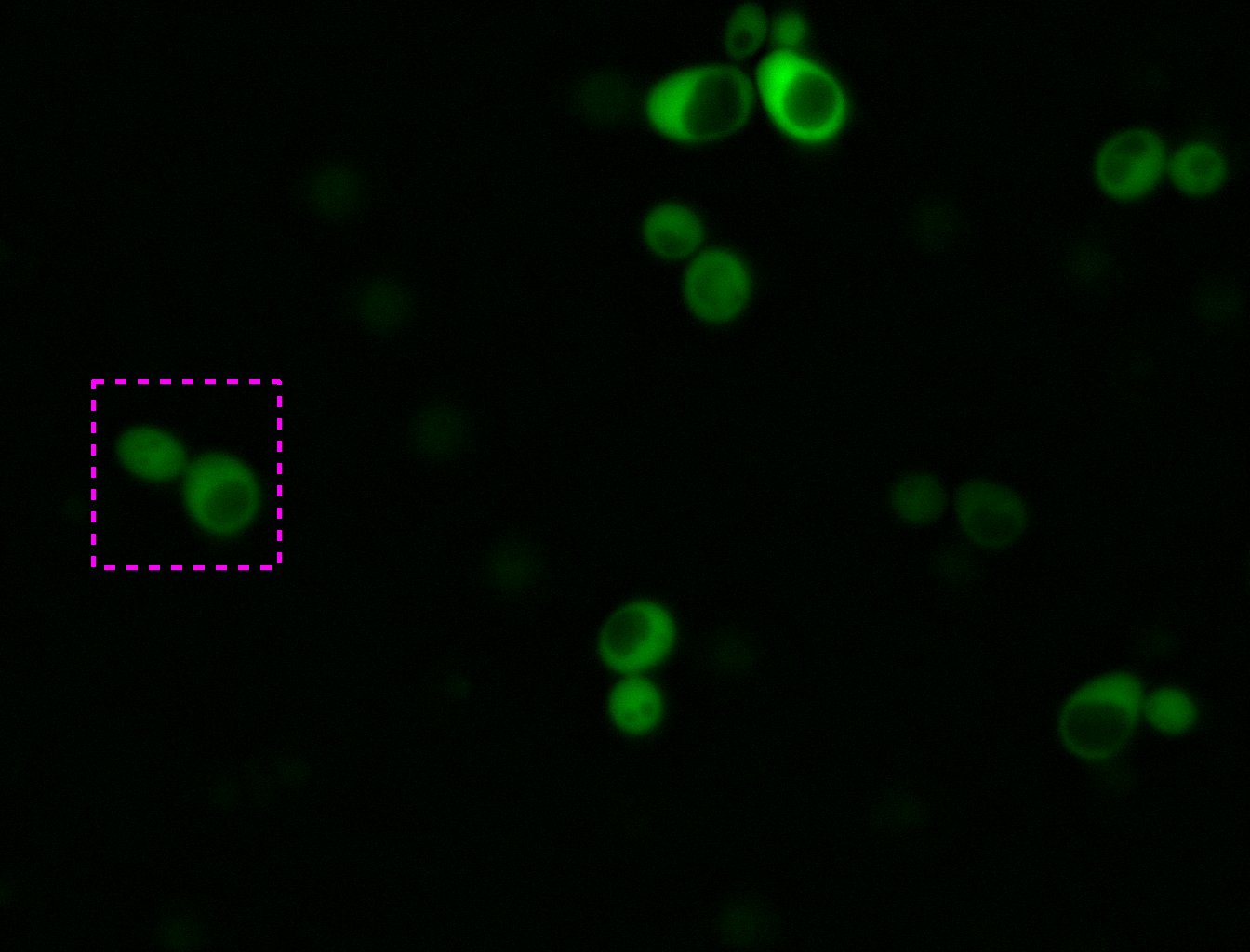

Supplement: Supplementary file 5 — Source data Fig. 4 [file 44319_2024_126_MOESM5_ESM.zip › Figure 4/4C/4C_Image_mNG-Oxr1_glucose_Green.jpg]

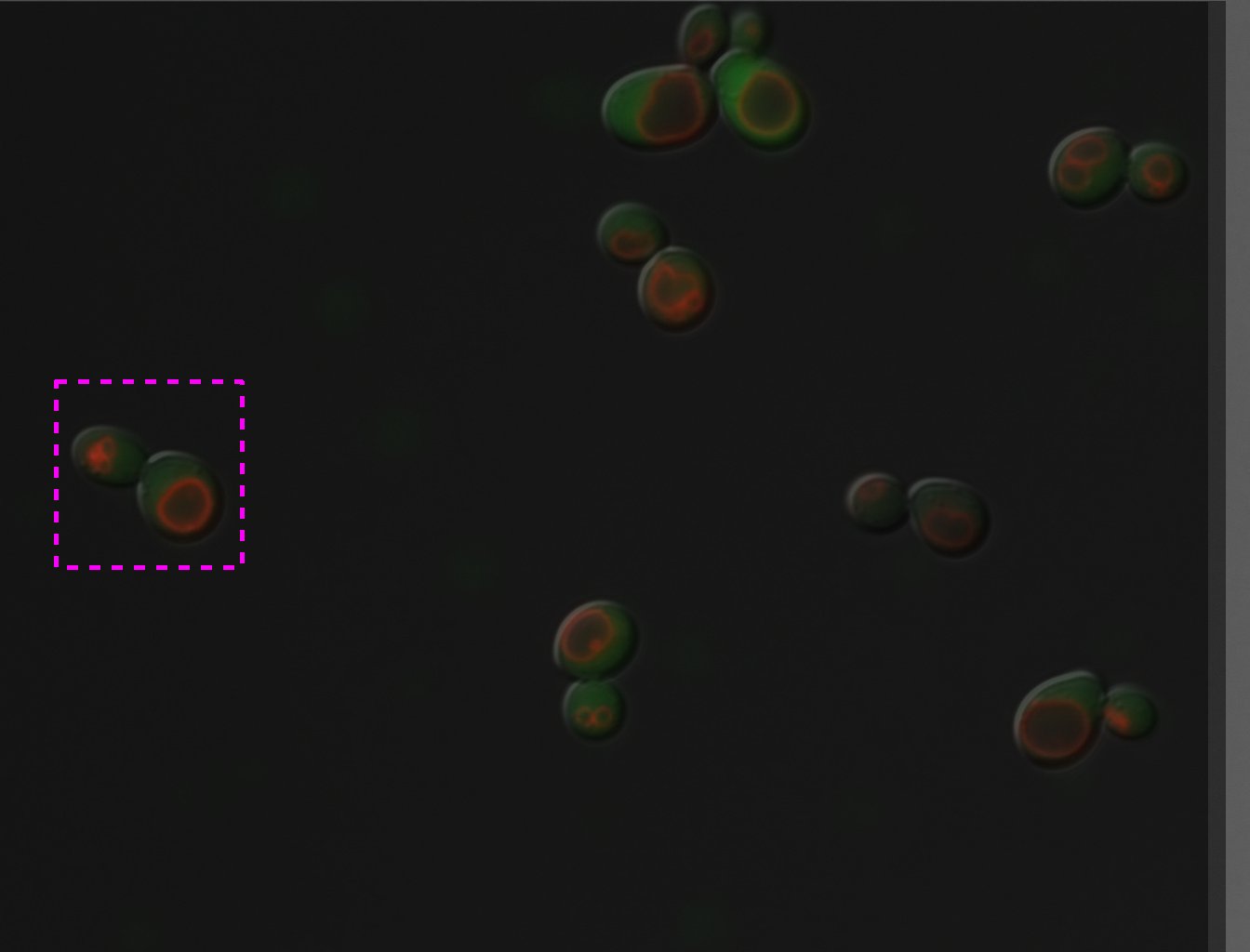

Supplement: Supplementary file 5 — Source data Fig. 4 [file 44319_2024_126_MOESM5_ESM.zip › Figure 4/4C/4C_Image_mNG-Oxr1_glucose_overlay.jpg]

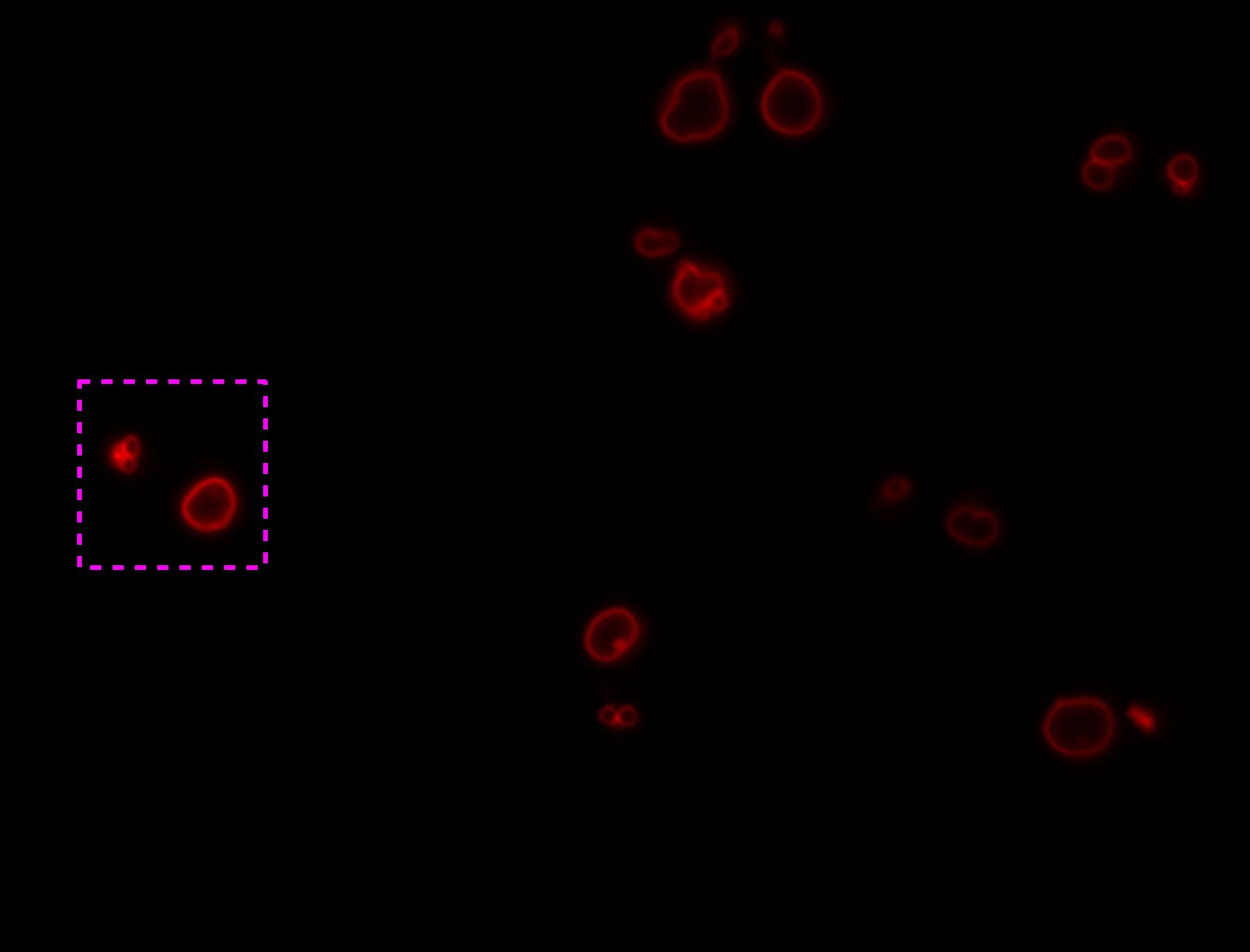

Supplement: Supplementary file 5 — Source data Fig. 4 [file 44319_2024_126_MOESM5_ESM.zip › Figure 4/4C/4C_Image_mNG-Oxr1_glucose_Red.jpg]

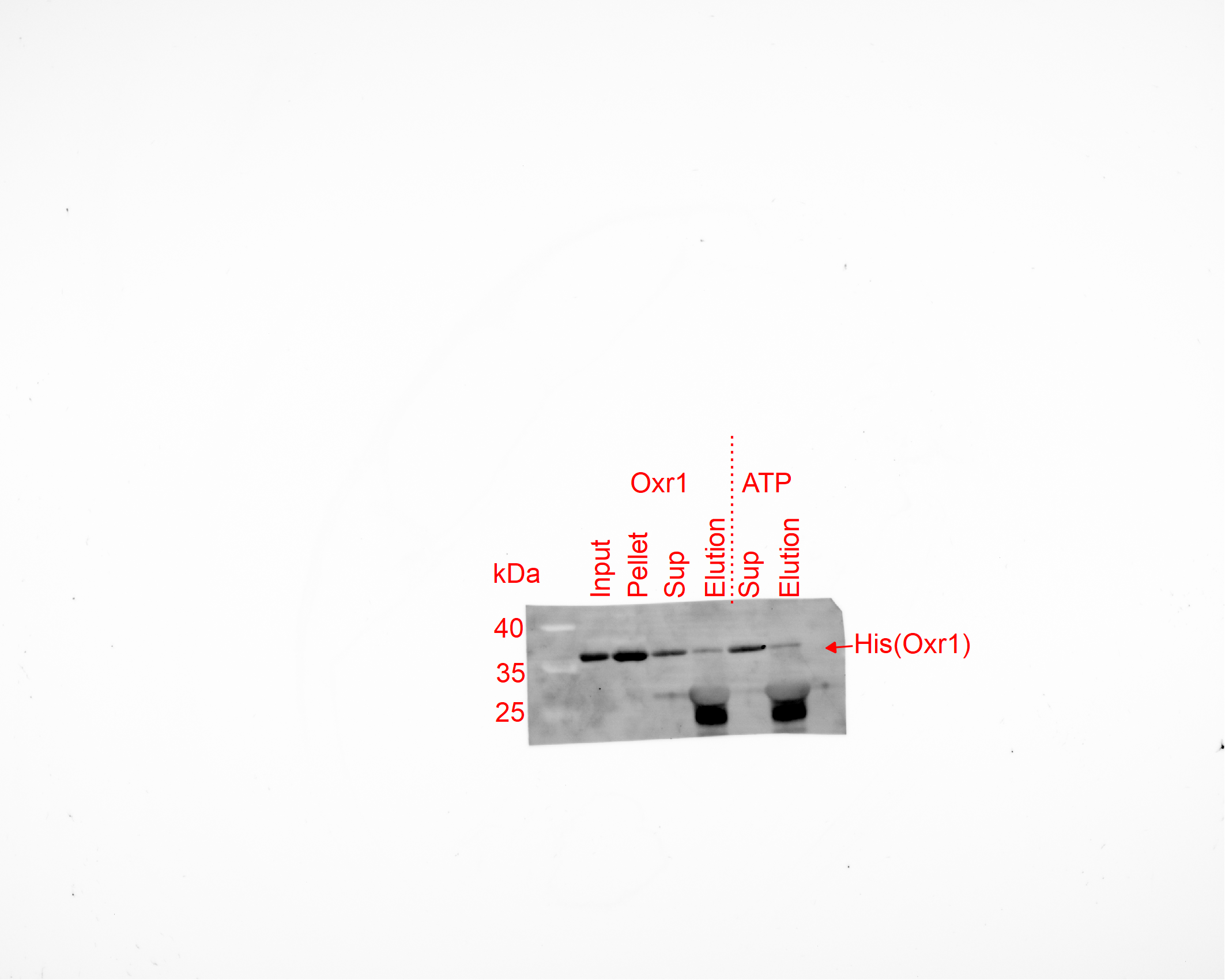

Supplement: Supplementary file 7 — Source data Fig. 6 [file 44319_2024_126_MOESM7_ESM.zip › Figure 6/6F/6F_WB_His(Oxr1).tif]

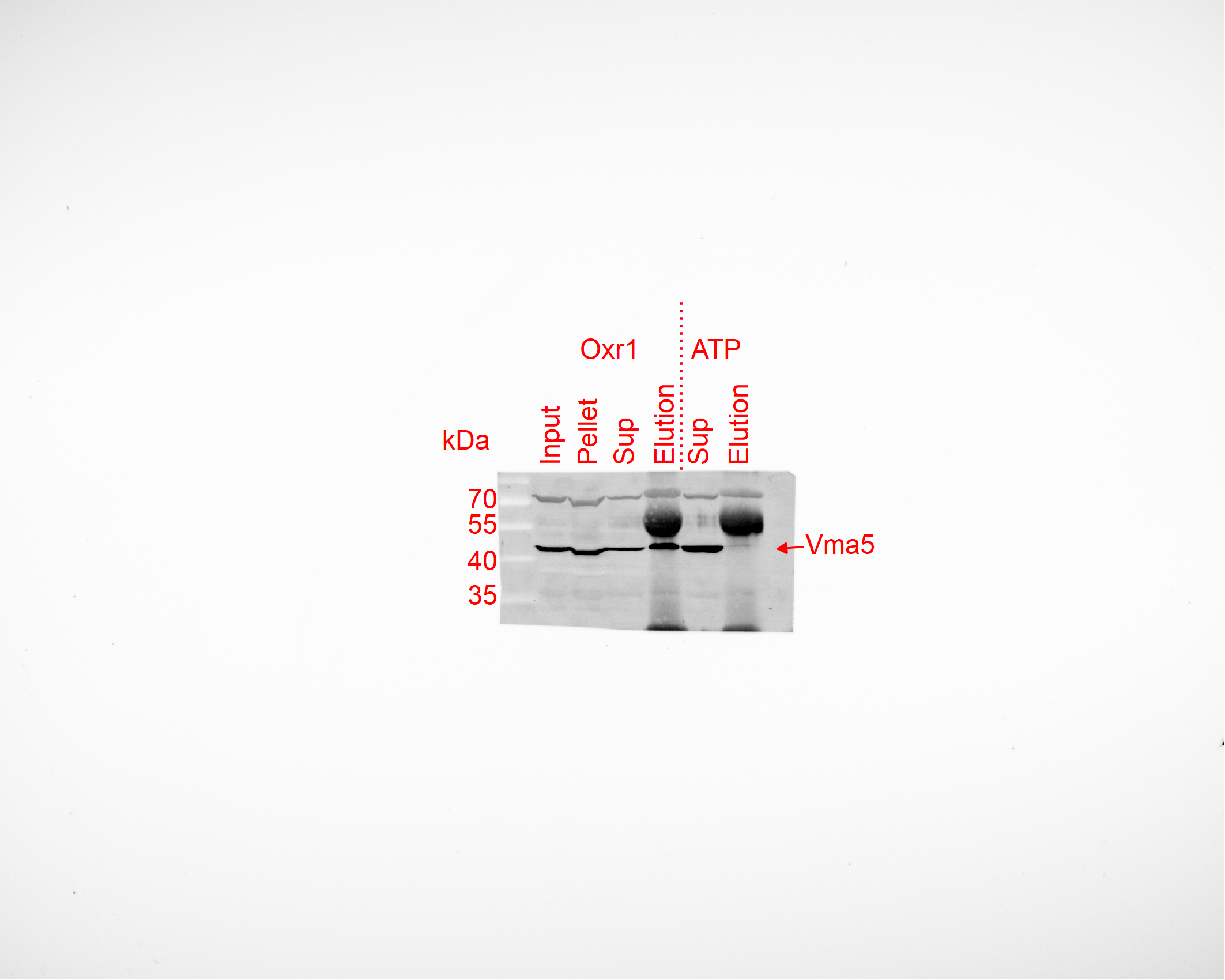

Supplement: Supplementary file 7 — Source data Fig. 6 [file 44319_2024_126_MOESM7_ESM.zip › Figure 6/6F/6F_WB_Vma5.tif]

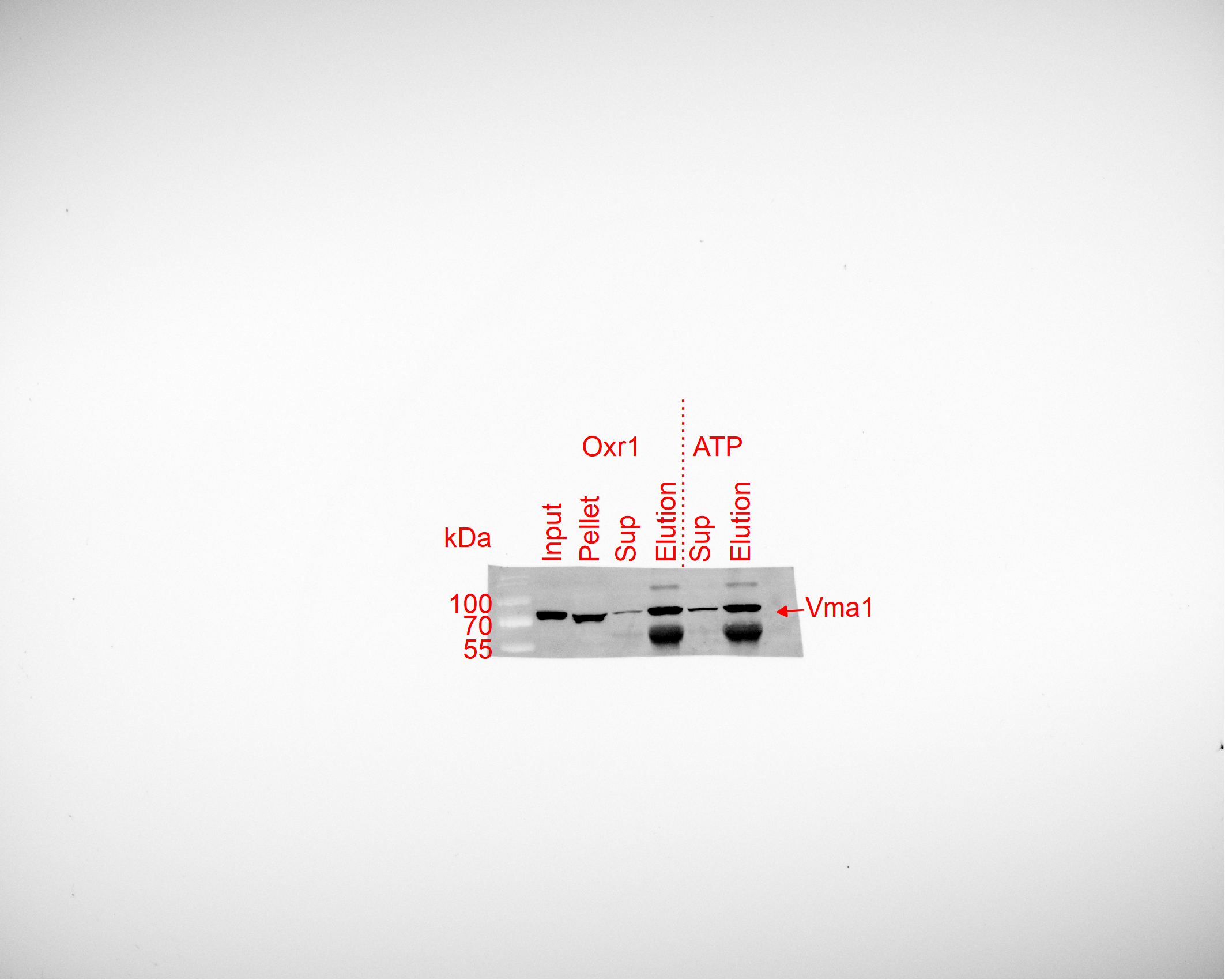

Supplement: Supplementary file 7 — Source data Fig. 6 [file 44319_2024_126_MOESM7_ESM.zip › Figure 6/6F/6F_WB_Vma1.tif]

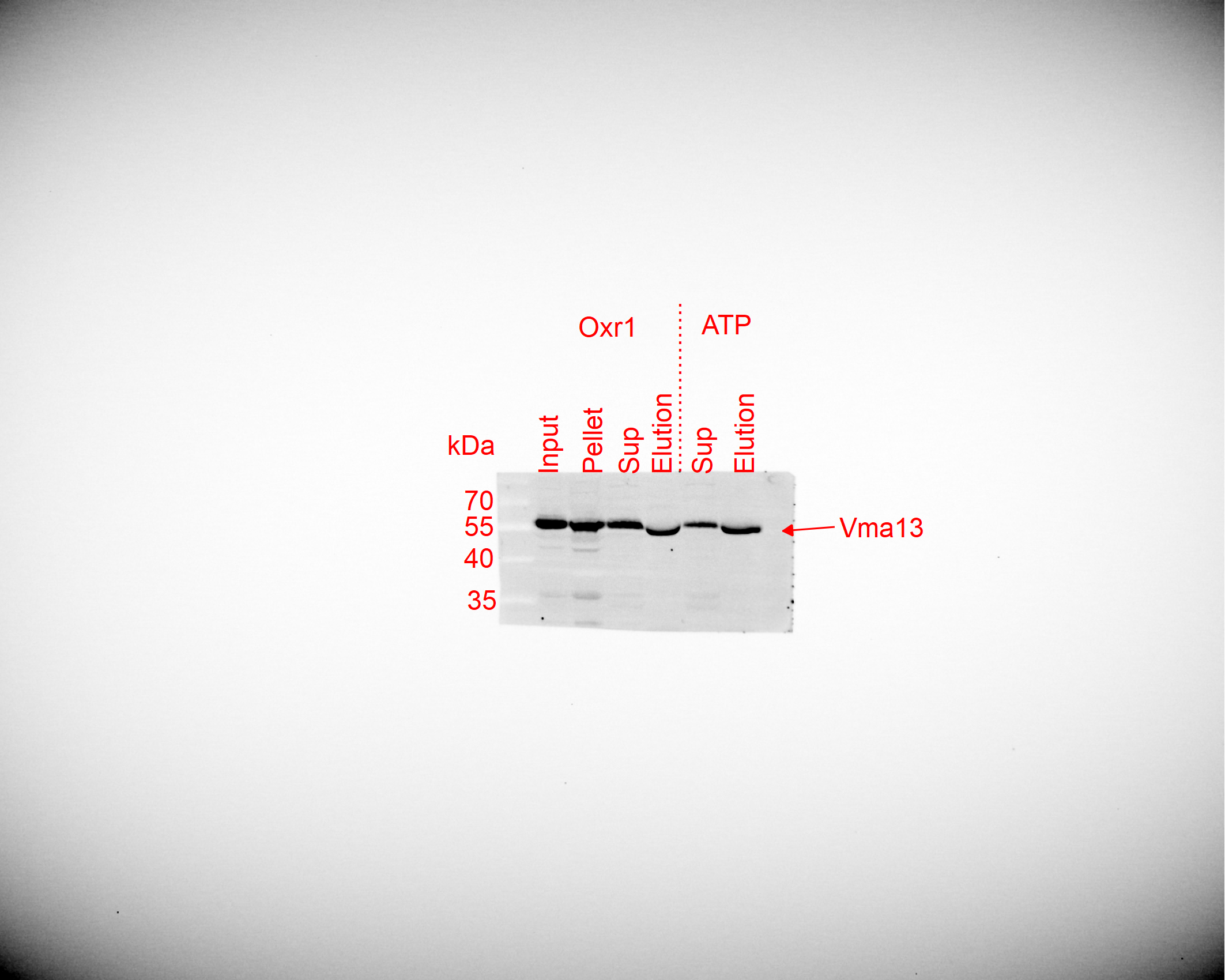

Supplement: Supplementary file 7 — Source data Fig. 6 [file 44319_2024_126_MOESM7_ESM.zip › Figure 6/6F/6F_WB_Vma13.tif]

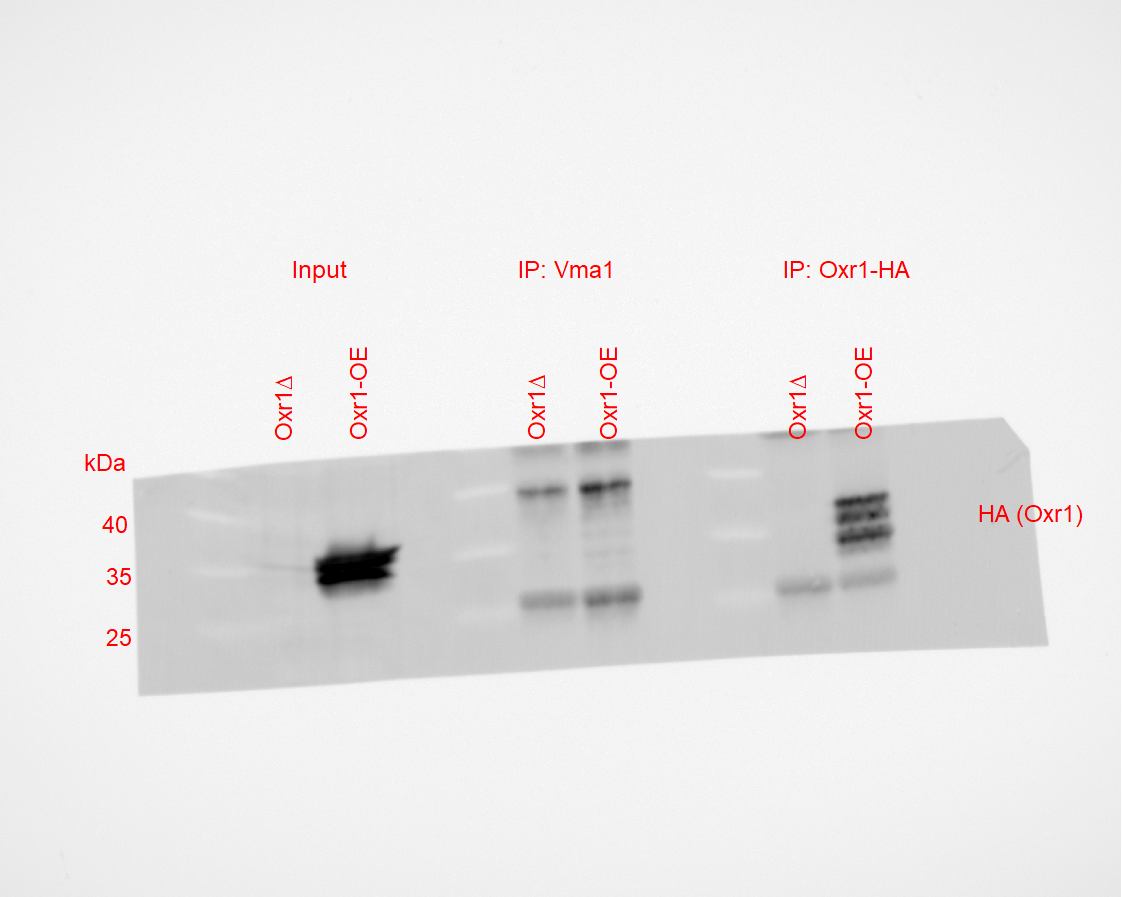

Supplement: Supplementary file 7 — Source data Fig. 6 [file 44319_2024_126_MOESM7_ESM.zip › Figure 6/6B/6B_WB_HA(Oxr1).tif]

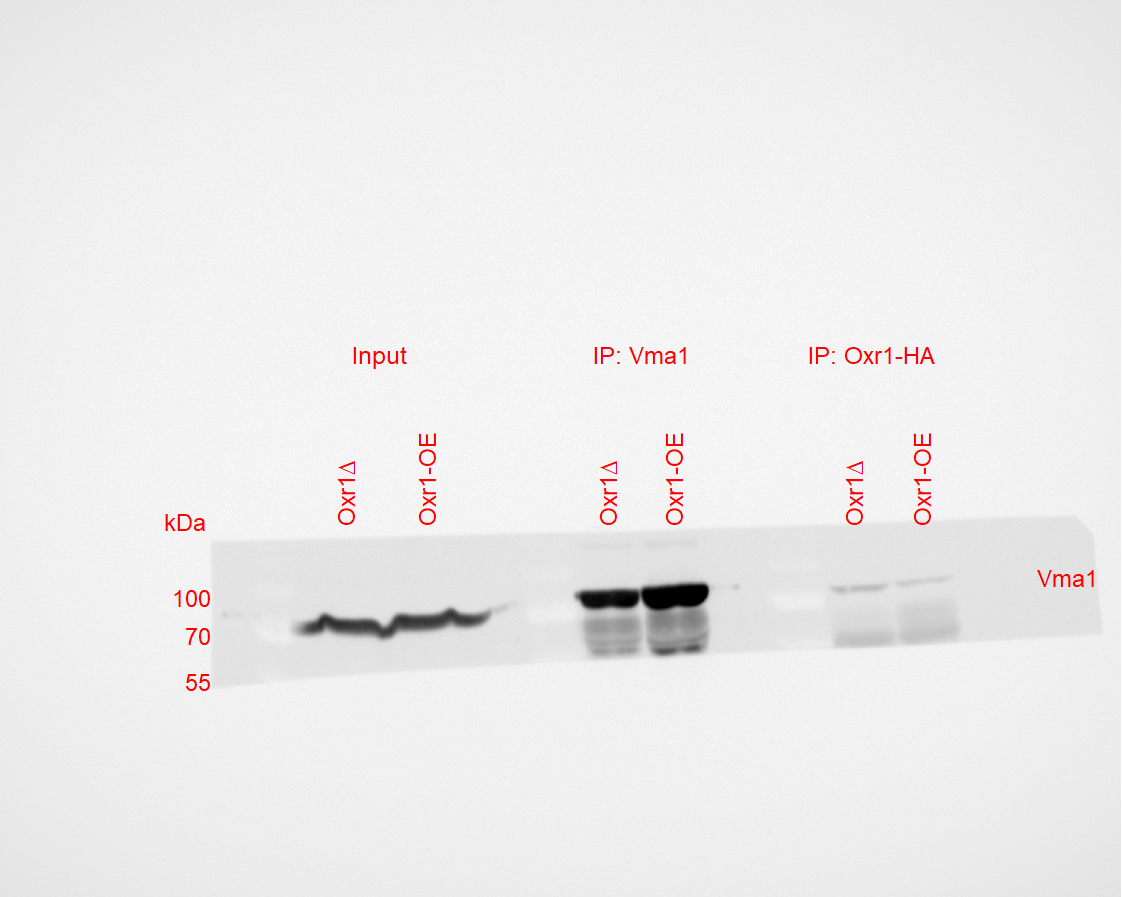

Supplement: Supplementary file 7 — Source data Fig. 6 [file 44319_2024_126_MOESM7_ESM.zip › Figure 6/6B/6B_WB_Vma1.tif]

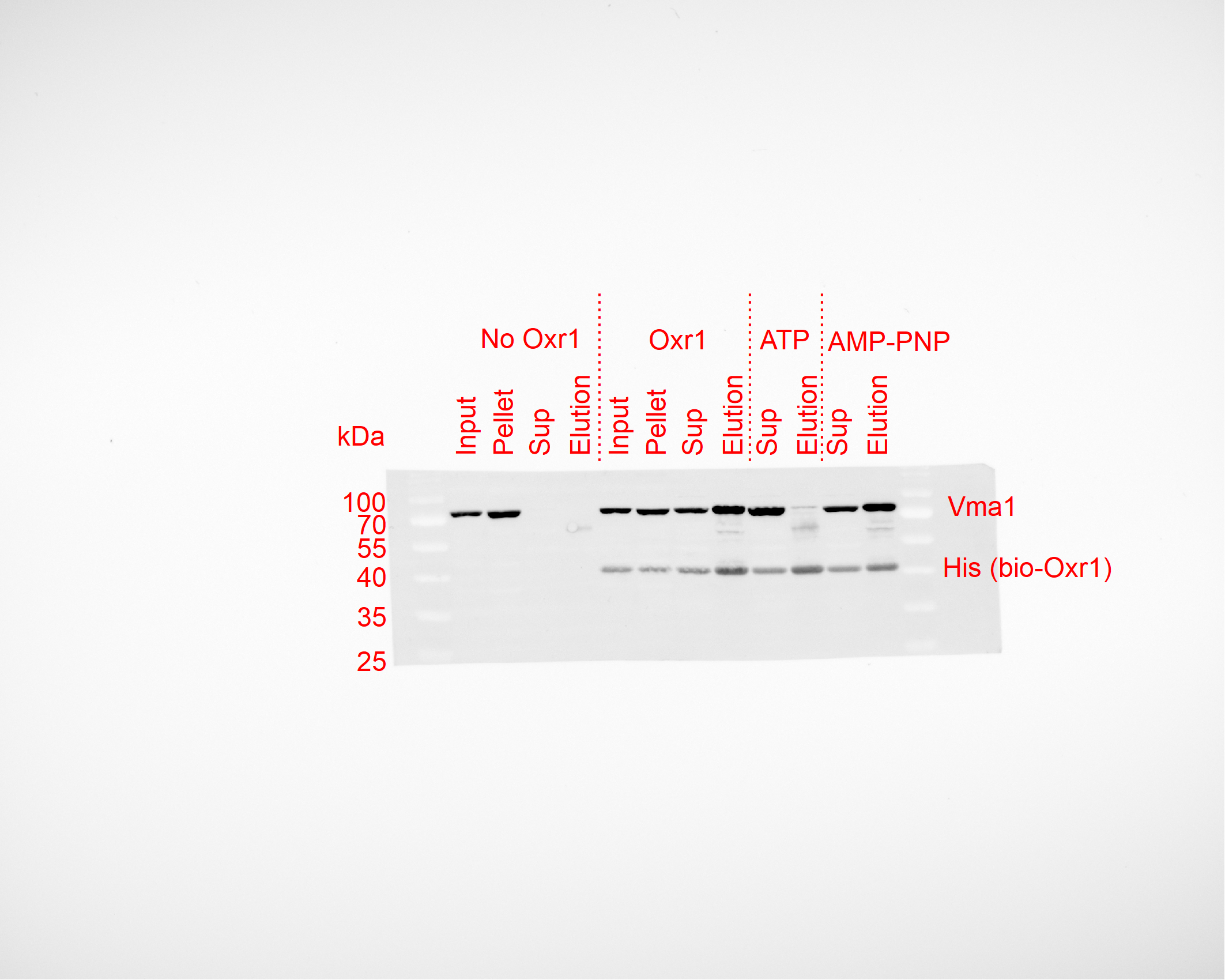

Supplement: Supplementary file 7 — Source data Fig. 6 [file 44319_2024_126_MOESM7_ESM.zip › Figure 6/6E/6E_WB_Vma1 and His(bio_Oxr1).tif]

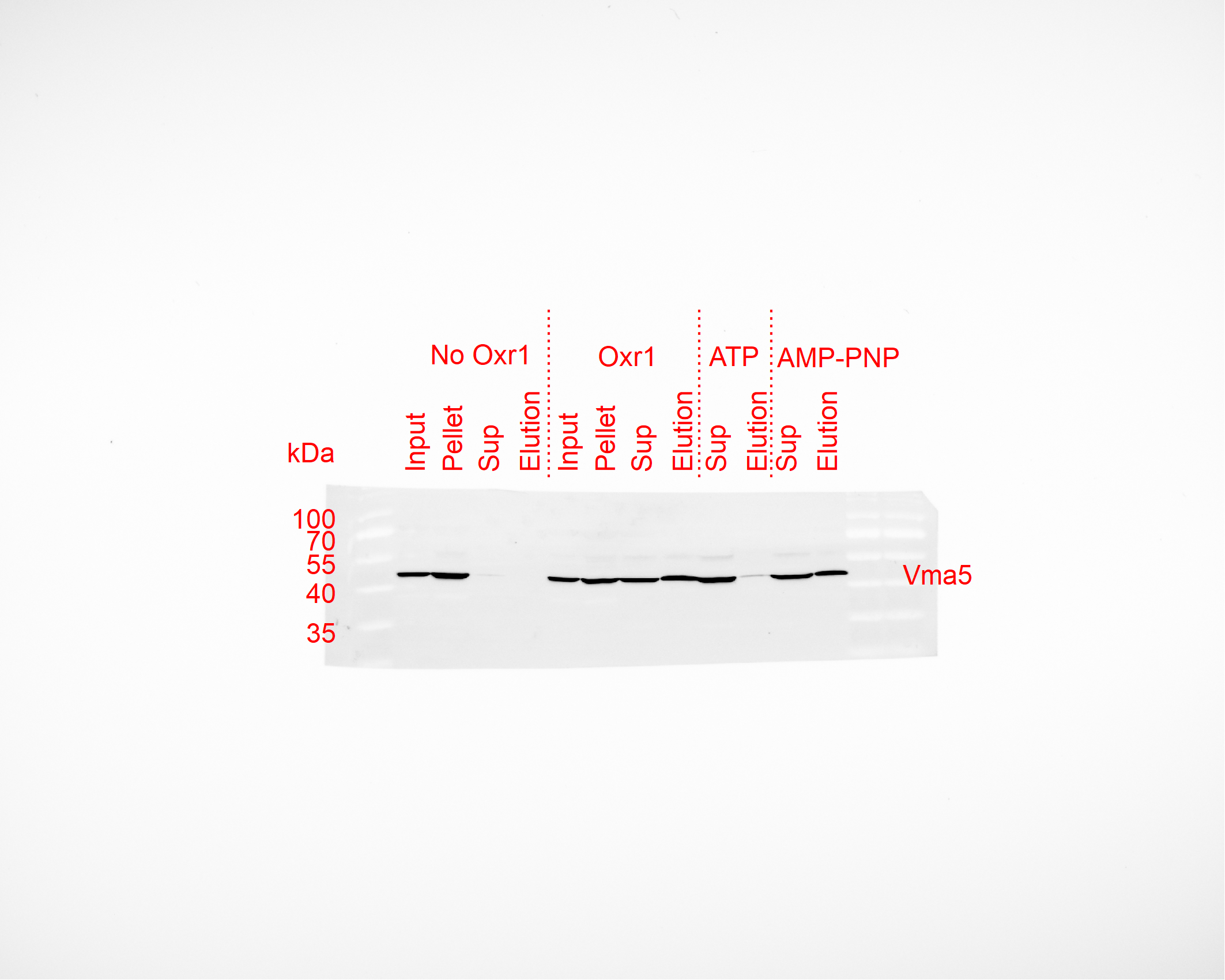

Supplement: Supplementary file 7 — Source data Fig. 6 [file 44319_2024_126_MOESM7_ESM.zip › Figure 6/6E/6E_WB_Vma5.tif]

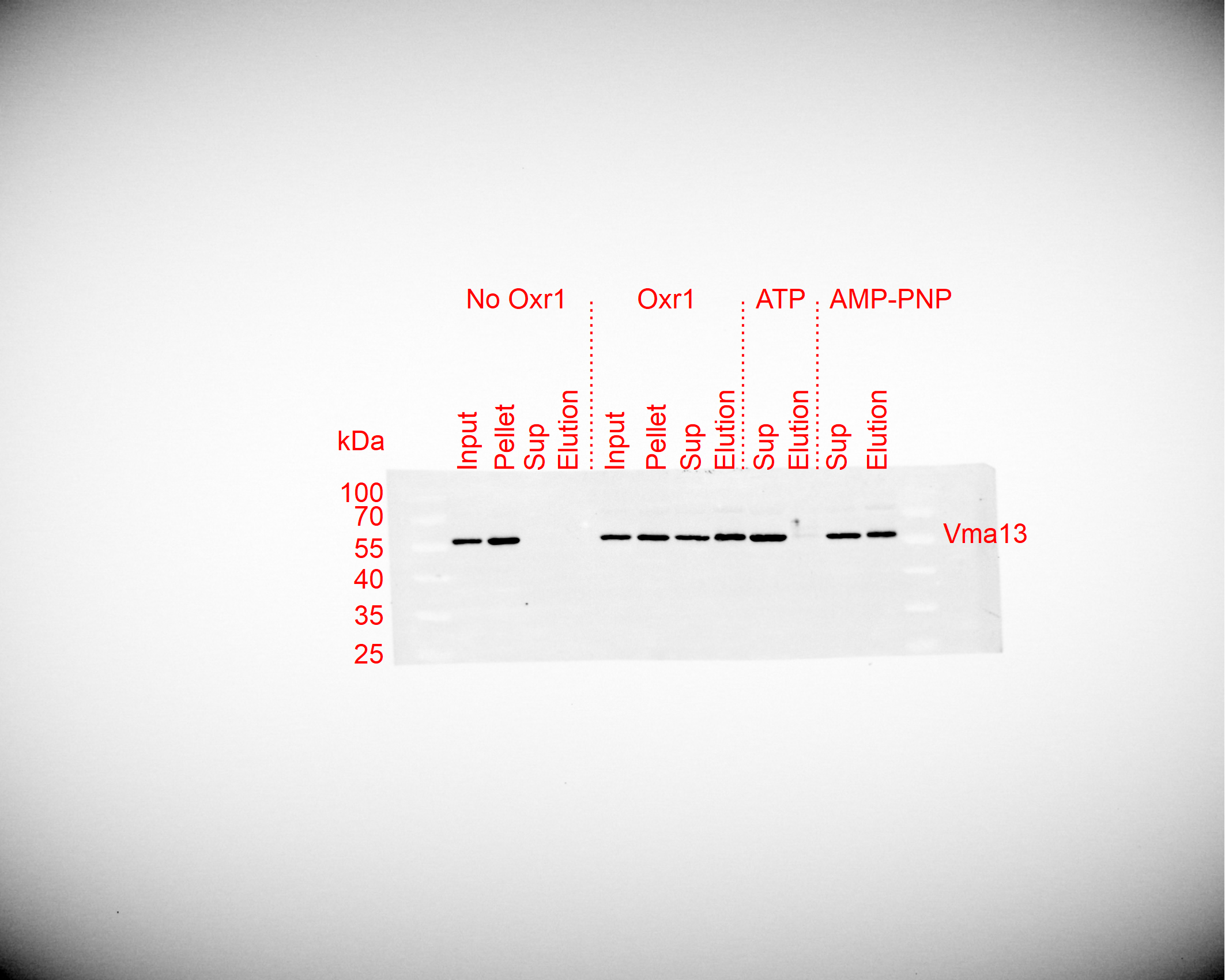

Supplement: Supplementary file 7 — Source data Fig. 6 [file 44319_2024_126_MOESM7_ESM.zip › Figure 6/6E/6E_WB_Vma13.tif]

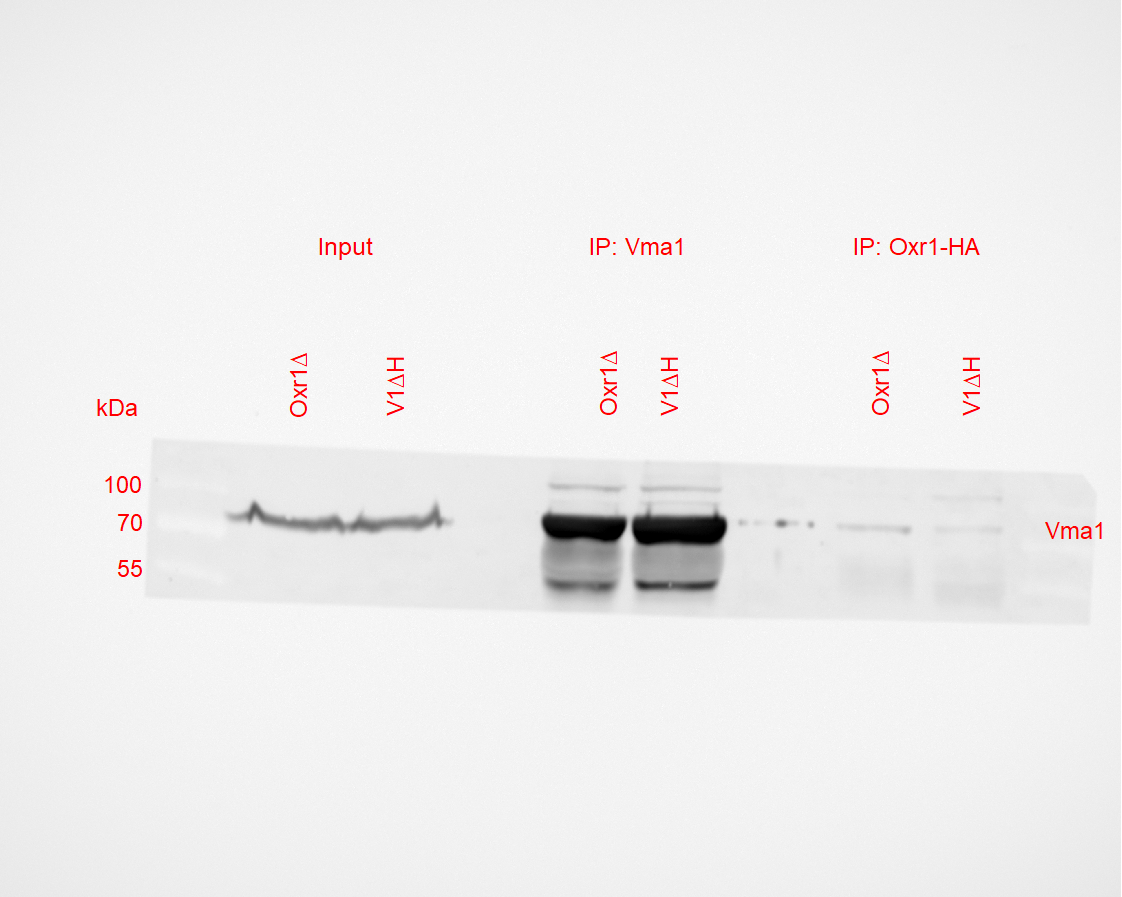

Supplement: Supplementary file 7 — Source data Fig. 6 [file 44319_2024_126_MOESM7_ESM.zip › Figure 6/6C/6C_WB_Vma1.tif]

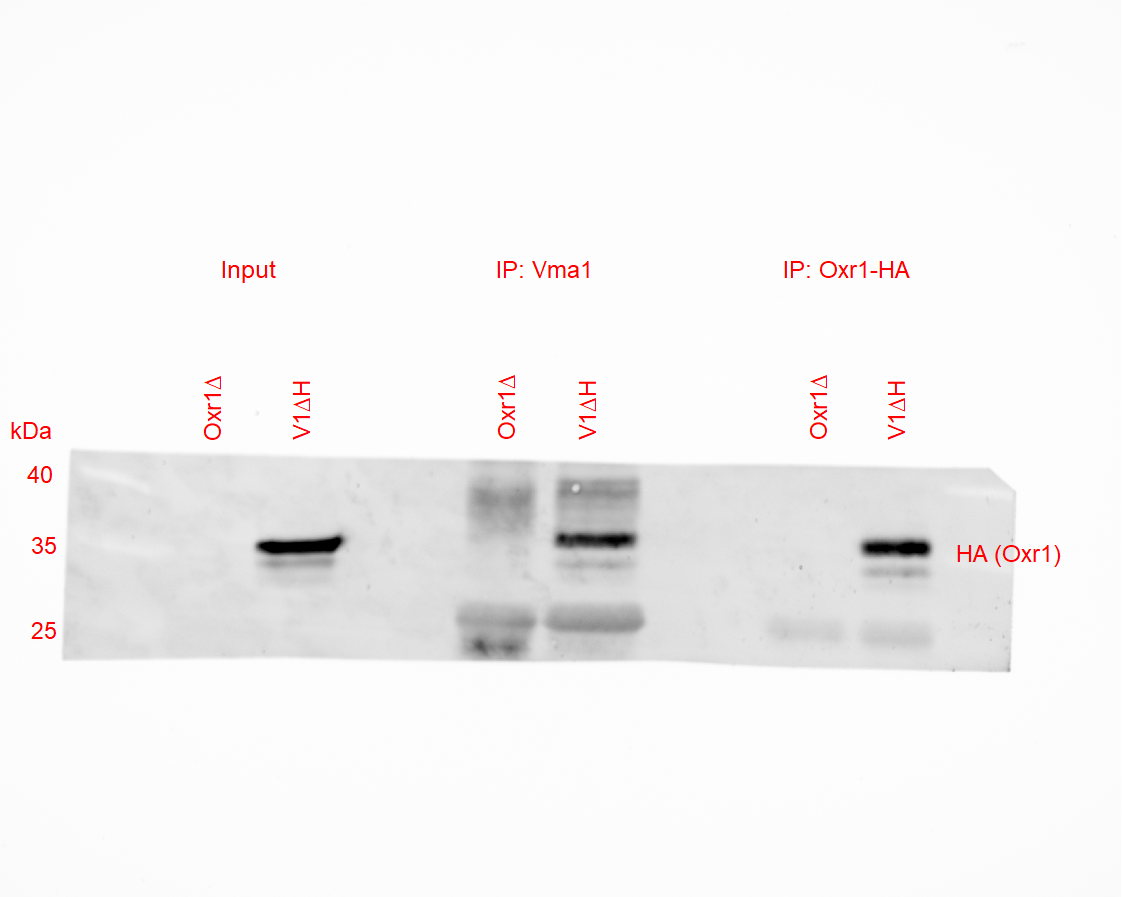

Supplement: Supplementary file 7 — Source data Fig. 6 [file 44319_2024_126_MOESM7_ESM.zip › Figure 6/6C/6C_WB_HA (Oxr1).tif]
