# Supplementary material for: Molecular mechanism of Oxr1p mediated disassembly of yeast V-ATPase
Source: EMBO Rep. 2024 Apr 2;25(5):15. doi: 10.1038/s44319-024-00126-5 (PMC11094088; doi:10.1038/s44319-024-00126-5)
Supplement: Supplementary file 9 — Expanded View Figures [file 44319_2024_126_MOESM9_ESM.pdf]

Expanded View Figures

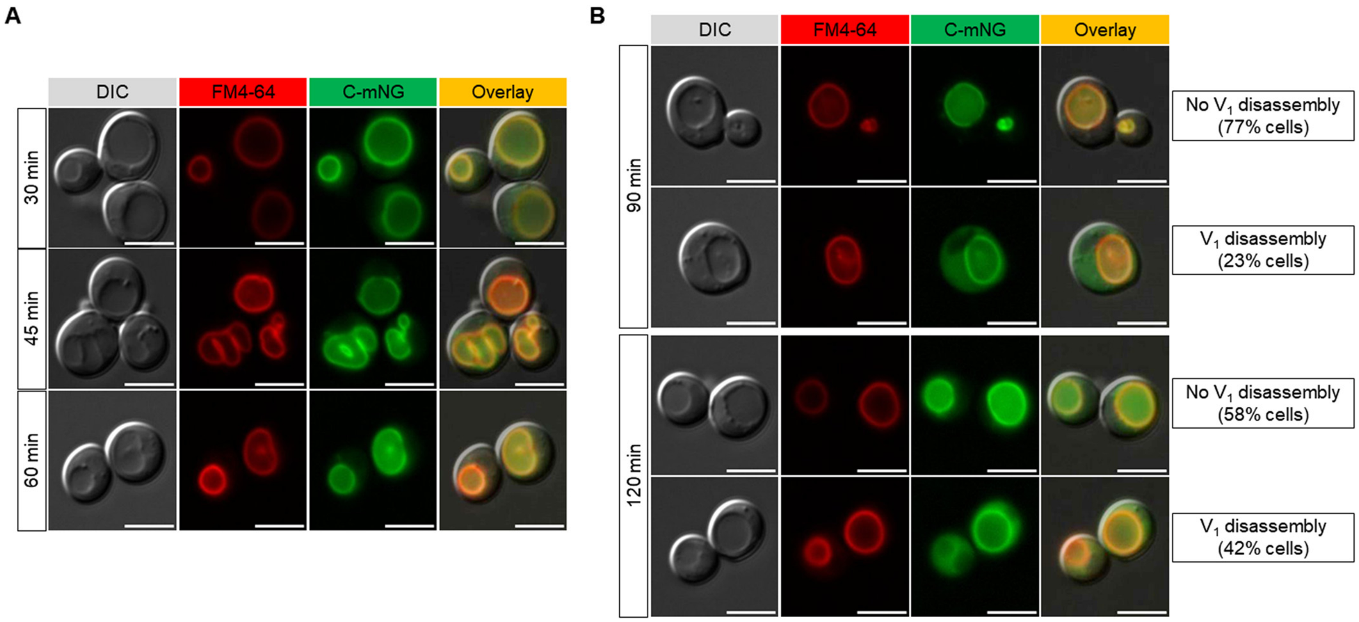

Figure EV1. Time course analysis of V_1 disassembly in *oxr1Δ* yeast strain.

(**A, B**) DIC and fluorescence images of *oxr1Δ* yeast cells expressing C-mNG after prolonged glucose deprivation (up to 2 h). Approximately 100 cells from two experiments were examined and cells showing diffuse staining (possibly indicating some form of V-ATPase disassembly) were expressed as a percentage of total cells at 1.5 and 2 h (**B**). Scale bar: 5 µm.

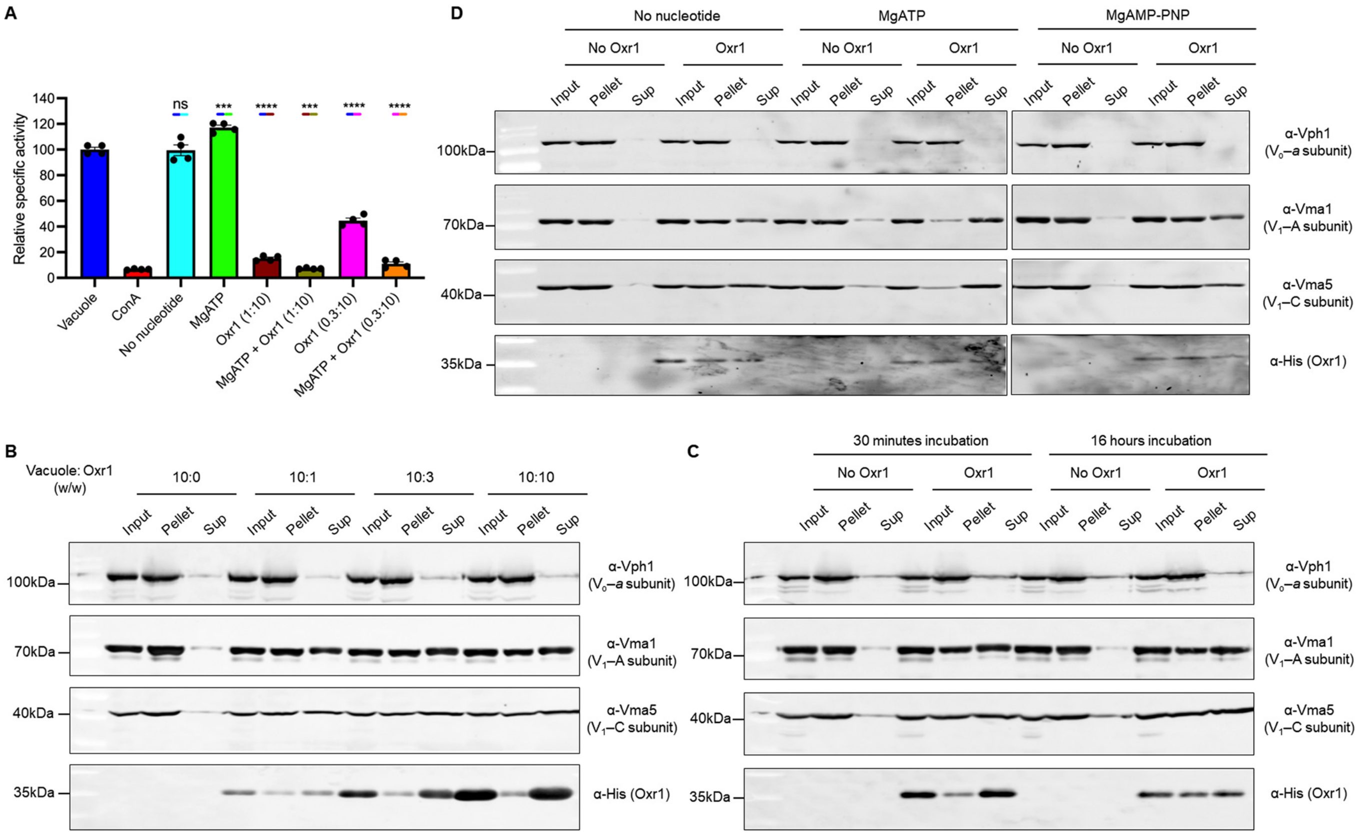

Figure EV2. Increase efficiency of Oxr1p-mediated vacuolar ATPase activity inhibition and disassembly in presence of nucleotides.

(**A**) Bar diagram of ATPase activities of purified vacuoles after incubating at room temperature for 30 min in absence and presence of Oxr1p (at 1:10 or 0.3:10 ratio (w/w) over total vacuolar protein) and 4 mM ATP. Relative activities were normalized against the starting activity of purified vacuoles (blue). ConA is used as an additional control (red). Individual data points of four tests from two biological preparations are shown. Data are presented as mean ± SEM. (**B**) Purified vacuoles were incubated with increasing concentrations of Oxr1p followed by ultracentrifugation. The extent of V_1 release from vacuoles was then determined by probing western blots of *pellet* and *sup* fractions with α-Vma1p (V_1 subunit A) and α-Vma5p (V_1 subunit C) antibodies. Increasing Oxr1p concentration does not lead to more V_1 disassembly from purified vacuoles, see equal amounts of Vma1p and Vma5p in the *sup* fraction in all Oxr1p treated samples. α-Vph1p (V_o subunit *a*) and α-His (Oxr1p) blots were included as controls. A representative of three experiments from two biological preparations is shown. (**C**) The effect of longer incubation times on Oxr1p-mediated V_1 disassembly was determined by western blot analysis as described in (**B**). Increasing the incubation time of Oxr1p treatment does not lead to more V_1 disassembly from purified vacuoles, see the equal amounts of Vma1p and Vma5p in the *sup* fraction after both short and long incubation with a 1:10 ratio (w/w) of Oxr1p over vacuolar protein. α-Vph1p (V_o subunit *a*) and α-His (Oxr1p) blots were included as controls. A representative of three experiments from two biological preparations is shown. (**D**) Purified vacuoles were incubated with Oxr1p (at 0.3:10 ratio (w/w) over vacuolar protein) in presence and absence of 4 mM nucleotides, and the extent of V_1 release from vacuoles was then determined by western blot analysis as described in (**B**). Relatively more intense bands of Vma1p and Vma5p in the ATP containing *sup* fraction indicates increased release of V_1 from V_o. α-Vph1p (V_o subunit *a*) and α-His (Oxr1p) blots were included as controls. A representative of three experiments from two biological preparations is shown. Data information: Statistical significance (**A**) was calculated in GraphPad Prism 9 using unpaired Student's *t* test (^{ns} indicates nonsignificant ($P > 0.05$); *** indicates $P \leq 0.001$; **** indicates $P \leq 0.0001$).

© The Author(s)

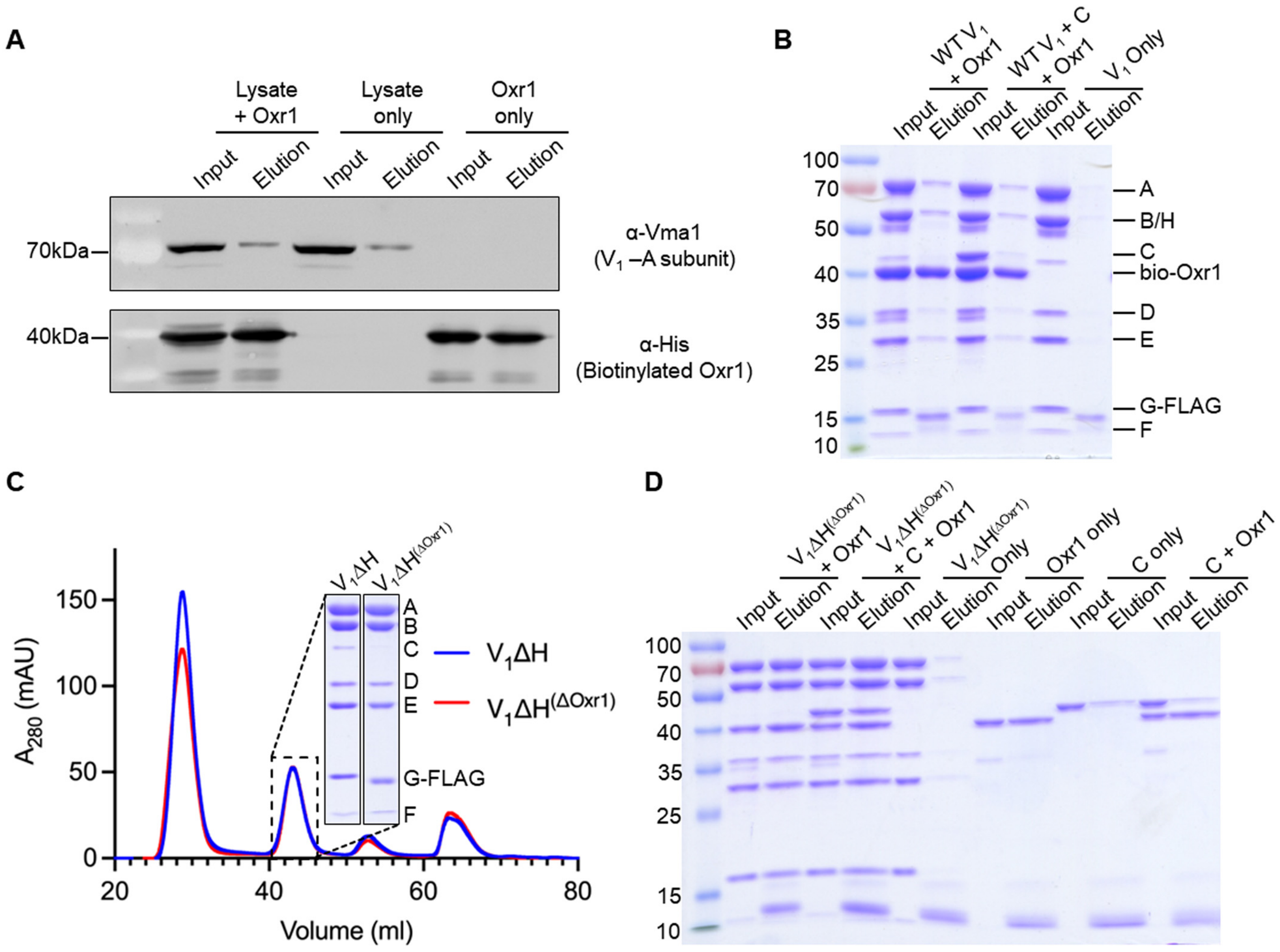

Figure EV3. Oxr1p binding to wild-type and mutant V_1 subcomplexes.

(**A**) Western blot of a pulldown of wild-type V_1 from cleared lysates of *oxr1Δ* cells using recombinant bio-Oxr1p as bait. The pulldown was carried out with lysate plus bio-Oxr1p, lysate plus buffer and bio-Oxr1p plus buffer. A representative of three experiments is shown. (**B**) Pulldown of purified wild-type V_1 in absence and presence of subunit C using recombinant bio-Oxr1p as bait as analyzed by SDS-PAGE. A representative of three experiments from two biological preparations is shown. (**C**) Size-exclusion chromatography (SEC) elution profile of $V_1ΔH$ and $V_1ΔH^{(ΔOxr1)}$ ($V_1ΔH$ purified from *oxr1Δ* background) on a Superose 6 increase HiScale column (16 mm × 400 mm). Inset, Coomassie blue stained SDS-PAGE gel of purified $V_1ΔH$ and $V_1ΔH^{(ΔOxr1)}$. Two independent $V_1ΔH^{(ΔOxr1)}$ preparations gave an average specific activity of ~44 ± 8 μmol × (min × mg)$^{-1}$. (**D**) Pulldown of $V_1ΔH^{(ΔOxr1)}$ by recombinant bio-Oxr1p was performed under the indicated conditions and analyzed by Coomassie blue stained SDS-PAGE gel. The analysis suggests that bio-Oxr1p can efficiently pull down purified $V_1ΔH^{(ΔOxr1)}$ with or without subunit C. Labeling as shown in (**B**). A representative of three experiments from two biological preparations is shown. Note that avidin monomer in the *elution* fractions runs at ~15 kDa in SDS-PAGE gels, in between the bands of FLAG-tagged subunit G and subunit F (**B**, **D**).
